# Dlk2 interacts with Syap1 to activate Akt signaling pathway during osteoclast formation

**DOI:** 10.1038/s41419-023-06107-1

**Published:** 2023-09-05

**Authors:** Xinwei Chen, Xuzhuo Chen, Rui Chao, Yexin Wang, Yi Mao, Baoting Fan, Yaosheng Zhang, Weifeng Xu, An Qin, Shanyong Zhang

**Affiliations:** 1 Department of Oral and Maxillofacial Surgery, Shanghai Ninth People’s Hospital, Shanghai Jiao Tong University School of Medicine; College of Stomatology, Shanghai Jiao Tong University; National Center for Stomatology; National Clinical Research Center for Oral Diseases; Shanghai Key Laboratory of Stomatology, Shanghai, People’s Republic of China; 2 Department of Stomatology, Shanghai Sixth People’s Hospital, Shanghai Jiao Tong University School of Medicine, Shanghai, People’s Republic of China; 3 Department of Orthopaedics, Shanghai Ninth People’s Hospital, Shanghai Jiao Tong University School of Medicine, Shanghai Key Laboratory of Orthopaedic Implant, Shanghai, People’s Republic of China

**Keywords:** Cell signalling, Bone, Mechanisms of disease

## Abstract

Excessive osteoclast formation and bone resorption are related to osteolytic diseases. Delta drosophila homolog-like 2 (Dlk2), a member of the epidermal growth factor (EGF)-like superfamily, reportedly regulates adipocyte differentiation, but its roles in bone homeostasis are unclear. In this study, we demonstrated that Dlk2 deletion in osteoclasts significantly inhibited osteoclast formation in vitro and contributed to a high-bone-mass phenotype in vivo. Importantly, Dlk2 was shown to interact with synapse-associated protein 1 (Syap1), which regulates Akt phosphorylation at Ser473. Dlk2 deletion inhibited Syap1-mediated activation of the Akt^Ser473^, ERK1/2 and p38 signaling cascades. Additionally, Dlk2 deficiency exhibits increased bone mass in ovariectomized mice. Our results reveal the important roles of the Dlk2-Syap1 signaling pathway in osteoclast differentiation and osteoclast-related bone disorders.

## Introduction

Bone remodeling is a dynamic process that requires the cooperation of many kinds of cells, including osteoclasts, osteoblasts, osteocytes and bone lining cells [1–5]. Bone remodeling is principally determined by the extent of osteoclast-mediated bone resorption and osteoblast-mediated bone formation and is critical to maintaining normal bone density and physiological functions. Bone loss is caused by excessive osteoclast formation and continuously degraded bone matrix, giving rise to bone disorders such as osteoporosis [6, 7], osteoarthritis [8, 9], rheumatoid arthritis [10] and periodontitis associated bone loss [11].

The epidermal growth factor (EGF)-like family comprises proteins containing one or more highly conserved EGF-like domains. Many of EGF-like family proteins have been proven to play essential roles in the regulation of bone biology [12]. Delta drosophila homolog-like 2 (Dlk2) is a member of the EGF-like family and is also known as epidermal growth factor-like domain multiple 9 (EGFL9). The Dlk2 gene is located on chromosome 6p21.1 in humans and chromosome 17 B3 in mice [13]. It is widely detected in mouse tissues, including lung, liver, brain, adipose tissues, testicle, ovary, placenta, kidney, cartilage and thymus tissues [13, 14] and in human breast cancer [15]. The protein encoded by Dlk2 in mice is a single-pass transmembrane glycoprotein consisting of 382 amino acids [16]. The Dlk2 protein is composed of three functional regions: an extracellular region which has six EGF-like repeated domains, a single transmembrane region and a short intracellular region [13]. A previous study demonstrated that Dlk2 regulated adipogenesis of 3T3-L1 and C3H10T1/2 cells [13] and inhibited chondrogenic differentiation of ATDC5 cells [17]. In addition, Dlk2 was involved in the regulation of breast cancer metastasis and melanoma cell proliferation [15, 18]. Interestingly, a high level of Dlk2 has been detected in mesoderm tissues such as the vertebral body in 16.5-day-old mouse embryos [14]. Moreover, in our previous study, we confirmed that Dlk2 is expressed in both osteoclasts and osteoblasts [16], suggesting that Dlk2 might contribute to skeletal development and bone remodeling. However, no information on the functional role of Dlk2 in these processes has been reported.

In this study, we generated three kinds of Dlk2 conditional knockout mice (Ctsk-Cre^+^;Dlk2^fl/fl^, LysM-Cre^+^;Dlk2^fl/fl^ and Prx1-Cre^+^;Dlk2^fl/fl^ mice) to elucidate the potential role of Dlk2 in the regulation of bone homeostasis. Dlk2 deletion in an osteoclast line significantly inhibited osteoclastogenesis and bone resorption activity, which led to the acquisition of a high-bone-mass phenotype in male mice and ovariectomized female mice. Deletion of Dlk2 in bone marrow mesenchymal stem cells (BMSCs) inhibited osteogenesis in vitro and resulted in a low-bone-mass phenotype in vivo. Importantly, we revealed that Dlk2 interacted with synapse-associated protein 1 (Syap1). Dlk2 deletion in osteoclasts suppressed osteoclastogenesis mainly by inhibiting Syap1 downstream cascades including the Akt^Ser473^, ERK1/2 and p38 phosphorylation. Our findings provide new insights into the prevention of osteoclast-related bone disorders.

## Materials and methods

### Mice

To generate Dlk2 conditional knockout mice, Dlk2^flox/wt^ mice (denoted as Dlk2^fl/wt^, strain No. T003770) were generated using CRISPR-Cas9 [19–21] technology under a background of C57BL/6 J by GemPharmatech Co., Ltd. (Nanjing, China). Briefly, sgRNAs were designed to specifically target the upstream and downstream regions of exon 4 (131 bp) of the mouse Dlk2 gene and were tested for on-target activity. A donor vector that carried a LoxP site and flanking target region was designed and coinjected with Cas9 mRNA and sgRNAs into zygotes. F0 founders were generated after zygotes were transferred into pseudopregnant females. F1 founders were generated by crossing positive F0 mice with C57BL6/J mice. The genotypes of the mice were identified with tail genome samples subjected to DNA PCR and agarose gel electrophoresis assay. To generate mice with Dlk2-deficient osteoclasts, Ctsk-Cre^+^;Dlk2^fl/wt^ mice were crossed with Dlk2^fl/fl^ mice (denoted as Ctsk-Cre^+^;Dlk2^fl/fl^, strain No. B004832) [22]. To generate mice with Dlk2-deficient macrophages, LysM-Cre^+^;Dlk2^fl/wt^ mice were crossed with LysM-Cre^+^;Dlk2^fl/wt^ mice (denoted as LysM-Cre^+^;Dlk2^fl/fl^). To generate mice with Dlk2-deficient osteoblasts, Prx1-Cre^+^;Dlk2^fl/wt^ mice were crossed with Dlk2^fl/fl^ mice (denoted as Prx1-Cre^+^;Dlk2^fl/fl^, strain No. B004833). Ctsk-Cre^-^;Dlk2^fl/fl^, LysM-Cre^-^;Dlk2^fl/fl^ and Prx1-Cre^-^;Dlk2^fl/fl^ mice were used as WT controls. Ctsk-Cre, LysM-Cre and Prx1-Cre transgenic mice were provided by GemPharmatech Co., Ltd. (Nanjing, China). Syap1-knockout mice (denoted as Syap1-KO, strain No. T038030) were generated using CRISPR-Cas9 technology under a background of C57BL/6 J by GemPharmatech Co., Ltd. (Nanjing, China). All the mice were raised under SPF conditions in the Department of Laboratory Animal Science at Shanghai Ninth People’s Hospital. All the animal experiments were carried out in randomized settings. All experimental procedures were carried out in strict accordance with the guidelines for the Ethical Conduct in the Care and Use of Nonhuman Animals in Research by the American Psychological Association.

### Micro-CT

Mouse femurs were separated and fixed in 4% paraformaldehyde (PFA) for 72 h and stored in 70% ethanol at 4 °C. A Scanco micro-CT scanner (Scanco medical μCT-100; Bassersdorf, Switzerland; resolution of 10 μm; X-ray source of 70 kV/200 μA; applied exposure time of 300 ms) was used. A region of interest (ROI) was defined from the distal growth plate extending proximally along the femur and consisted of 200 slices (4-μm slice) and lumber 3 vertebrae. All the trabecular bone images of the ROI were reconstructed and analyzed by CT Evaluation software (Scanco Medical).

### Bone histomorphometry

The mice were intraperitoneally injected with 8 mg/kg calcein solution at 6 weeks of age and 20 mg/kg alizarin red at 7 weeks of age. Then, the mice were sacrificed at 8 weeks of age, and their tibias were separated as described and embedded without decalcification in methyl methacrylate (MMA) resin [23, 24]. The sections were sliced with a Leica RM2255 microtome (Leica, Germany) to a thickness of 5 μm and then used for Von Kossa and TRAP staining. Blank sections were used for analysis of double fluorescence labeling. Bone histomorphometric analysis was performed with BIOQUANT OSTEO software (BIOQUANT, USA). All the trabecular bones under the growth plates of the proximal tibias were analyzed.

### ELISAs

Blood was obtained by retro-orbital collection, and after 30 min of centrifugation at 4 °C, serum was collected from the blood. The concentrations of CTX-I and PINP were detected by serum ELISA kits (Lengton, Shanghai, China).

### In vitro osteoclast differentiation assay

Bone marrow-derived macrophages (BMMs) were isolated from the tibias and femurs of 8-week-old mice and prepared as previously described [25–27]. Briefly, BMMs were cultured for 5 days in minimum essential medium (MEM)-α media containing 10% fetal bovine serum (FBS), 100 U/ml penicillin/streptomycin and 30 ng/ml M-CSF. For osteoclast differentiation, BMMs were seeded in 96-well plates (1 × 10^4^ cells/well) and stimulated with 30 ng/ml M-CSF and 50 ng/ml RANKL for 5 days. Mature osteoclasts were fixed in 4% PFA for 20 min and stained with TRAP solution for 30 min. TRAP^+^ multinucleated osteoclasts were counted with ImageJ software (NIH, Bethesda, MD).

### In vitro resorptive function assay

BMM-derived osteoclasts were seeded in Stripwell osteo assay plates (Corning, NY), stimulated with M-CSF and RANKL for 9 days and removed with 2% sodium hypochlorite solution for 2 min. The bone resorption pits were counted with ImageJ software.

### In vitro osteoblast differentiation assay

BMSCs were flushed from the tibias and femurs of 8-week-old mice and cultured in MEM-α containing 10% FBS and 100 U/ml penicillin/streptomycin. For osteoblast differentiation, BMSCs were seeded in 24-well plates (8 × 10^4^ cells/well) and stimulated with 10 mM β-glycerophosphate, 50 μg/ml ascorbic acid and 10^−7^ mM dexamethasone over a 14-day period [28–30]. Osteoblasts were fixed in 4% PFA for 20 min and stained with an ALP staining kit and alizarin red solution. For quantitation, alizarin red was dissolved in 10% cetylpyridinium chloride, and the absorbance was determined at 490 nm with a spectrophotometer (ThermoFisher Scientific, USA).

### Coimmunoprecipitation (co-IP) combined with liquid chromatography-tandem mass spectrometry (LC-MS/MS) assay

HEK-293T cells (ATCC) were cultured in high-glucose DMEM containing 10% FBS and 100 U/ml penicillin/streptomycin. For the coimmunoprecipitation assay, the cells were transfected with 10 μg of the indicated plasmids using polyethyleneimine solution for 48 h, lysed with IP lysis buffer for 30 min and incubated with anti-Flag M2 magnetic beads at 4 °C for 8 h. Then, the protein-bead complexes were washed 3 times with ice-cold IP lysis buffer and prepared for LC-MS/MS analysis. The LC-MS/MS analysis was provided by Shanghai Bioprofile Technology Company Ltd. (Shanghai, China). Plasmids carrying pLVX-CMV-3flag, pLVX-CMV-Dlk2-3flag, pLVX-CMV-Dlk2-myc, and pLVX-CMV-Syap1-3flag were purchased from Genomeditech (Shanghai, China).

### Western blot

Total cellular proteins were collected at the indicated time points using SDS lysis buffer supplemented with protease and phosphatase inhibitor cocktail. Western blots were performed according to the description in our previous study [25]. The antibodies and primary reagents were listed in Table S1.

### Quantitative real-time PCR (qPCR) assay

Total RNA was extracted from cultured cells or bone tissues using TRIzol reagent and was reverse-transcribed into cDNA with a PrimeScript RT reagent kit. The qPCRs were performed on a StepOnePlus Real-Time PCR system (Life Technologies, Applied Biosystems, USA) using a TB Green Premix Ex Taq kit. The sequences of the primers used in this study are listed in Table S2.

### Cell transfection

The siRNAs used in this study were designed and synthesized by RiboBio (Guangzhou, China). The siRNA transfection was performed with riboFECT transfection buffer (RiboBio). The sequences of the siRNAs are listed in Table S3. The Syap1-shRNA, Dlk2-shRNA, Syap1-overexpressing and Dlk2-overexpressing lentiviruses used for transfection were purchased from Genomeditech (Shanghai, China) (Table S4). The Cre adenovirus used for transfection was purchased from GeneChem (Shanghai, China).

### Ovariectomy (OVX) model

OVX was performed on 12-week-old Ctsk-Cre^−^;Dlk2^fl/fl^ (WT) and Ctsk-Cre^+^;Dlk2^fl/fl^ (CKO) female mice as briefly described [31–33]. After 2 months, the mice were sacrificed, and the femurs were collected and fixed in 4% PFA. The femurs were scanned by micro-CT and analyzed as described herein.

### Histological staining and immunofluorescence staining

The PFA-fixed femurs were embedded in paraffin after 2 weeks of decalcification in 10% EDTA and sliced for TRAP, hematoxylin and eosin (H&E) and immunofluorescence staining. For immunofluorescence staining, the sections were permeabilized with 0.1% Triton X-100, blocked with 5% goat serum and incubated overnight with primary antibodies at 4 °C and incubated with Alexa Fluor 555-conjugated secondary antibody for 1 h. DAPI was used to detect nuclei. The sections were imaged under a fluorescence microscope (Leica, Germany) or a light microscope (ZEISS, Germany) and analyzed with ImageJ software.

### Statistics

The results are analyzed in blind settings and presented as mean ± SD as determined with GraphPad Prism 8 software. Significant differences were determined by two-tailed Student’s tests using SPSS 13.0 software (Chicago), with *p* < 0.05 considered to be significant.

## Results

### Ctsk-Cre^+^;Dlk2^fl/fl^ mice developed a high-bone-mass phenotype

The qPCR results showed that Dlk2 was expressed in a wide range of tissues and prevalently in bone and spleen (Fig. S1A). Western blot analysis revealed that Dlk2 expression was continuously increased during osteoclast formation and decreased during osteoblast differentiation (Fig. S1B, C).

**Fig. 1:**
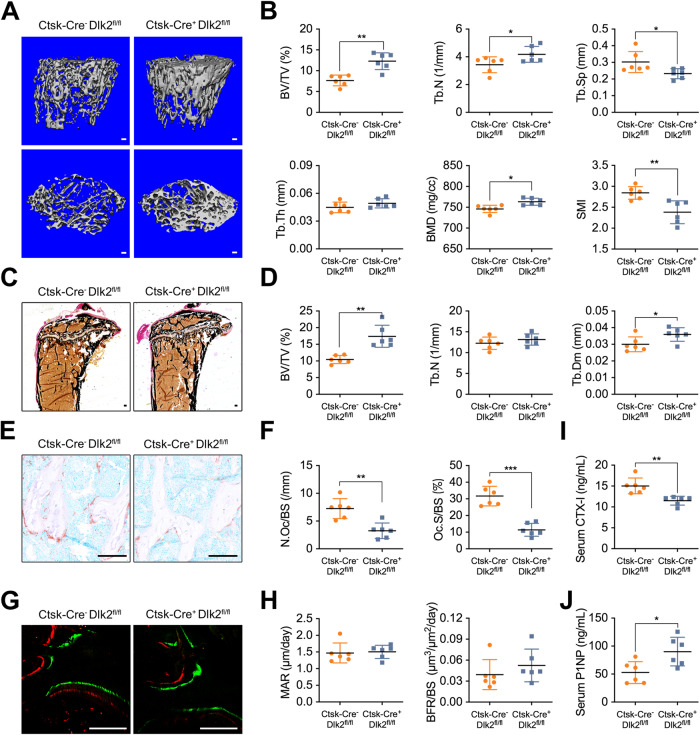
Ctsk-Cre^+^;Dlk2^fl/fl^ mice acquire a high-bone-mass phenotype. **A** Representative 3D micro-CT reconstruction images of distal femurs from 8-week-old Ctsk-Cre^-^;Dlk2^fl/fl^ (WT) and Ctsk-Cre^+^;Dlk2^fl/fl^ male mice. **B** Quantitative micro-CT analysis of the images shown in (A); BT/TV, bone volume percentage/tissue volume ratio; Tb.N, trabecular bone number; Tb.Sp, trabecular bone separation; Tb.Th, trabecular bone thickness; BMD, bone mineral density; SMI, structure model index. **C** Von Kossa staining of undecalcified tibial sections from 8-week-old WT and Ctsk-Cre^+^;Dlk2^fl/fl^ male mice. **D** Bone histomorphometric analysis of the Von Kossa-stained sections shown in **C**; BT/TV, bone volume percentage/tissue volume ratio; Tb.N, trabecular bone number; Tb.Dm, trabecular bone diameter. **E** TRAP staining of undecalcified tibial sections from 8-week-old WT and Ctsk-Cre^+^;Dlk2^fl/fl^ male mice. **F** Bone histomorphometric analysis of the TRAP-stained sections shown in **E**; N.Oc/BS, osteoclast number/bone surface ratio; Oc.S/BS, osteoclast surface/bone surface ratio. **G** Double label staining with calcein and alizarin red of undecalcified tibial sections from 8-week-old WT and Ctsk-Cre^+^;Dlk2^fl/fl^ male mice. **H** Bone histomorphometric analysis of the double label stained sections shown in **G**; MAR, mineral apposition rate; BFR/BS, bone formation rate. **I** Serum levels of CTX-I. **J** Serum levels of P1NP. (Scale bar, 100 μm, mean ± SD, Student’s tests, *p < 0.05, **p < 0.01, ***p < 0.001, *n* = 6 for each genotype).

To investigate the potential role of Dlk2 in osteoclasts and bone diseases, we bred osteoclast-specific Dlk2-deficient mice, Ctsk-Cre^+^;Dlk2^fl/fl^ mice (Fig. S1D). Generally, the weight, body size and survival rate of 8-week-old Ctsk-Cre^+^;Dlk2^fl/fl^ male mice were not significantly different from those of their WT littermates. The Ctsk-Cre^+^;Dlk2^fl/fl^ mice (8 weeks old, *n* = 6) exhibited a higher bone volume/tissue volume ratio (BV/TV) and bone mineral density (BMD) than the WT mice (Fig. 1A, B). Moreover, the trabecular bone number (Tb.N) of Ctsk-Cre^+^;Dlk2^fl/fl^ group was significantly increased, while trabecular bone separation (Tb.Sp) was diminished (Fig. 1B). The lumber 3 vertebrae in Ctsk-Cre^+^;Dlk2^fl/fl^ mice also exhibited an increase in bone mass (Fig. S1F, G). Von Kossa staining of tibias confirmed the BV/TV ratio was significantly increased in Ctsk-Cre^+^;Dlk2^fl/fl^ mice (Fig. 1C, D). Furthermore, TRAP staining revealed that the osteoclast number/bone surface ratio (N.Oc/BS) and osteoclast surface/bone surface ratio (Oc.S/BS) of Ctsk-Cre^+^;Dlk2^fl/fl^ mice were significantly reduced (Fig. 1E, F). Similarly, the serum CTX-I level in Ctsk-Cre^+^;Dlk2^fl/fl^ mice was also significantly reduced (Fig. 1I). We also observed a significant decrease in osteoclast-specific markers, including *Trap*, *Ctsk*, *Nfatc1* and *Atp6v0d2*, in bone tissues of Ctsk-Cre^+^;Dlk2^fl/fl^ mice (Fig. S2A), suggesting lower osteoclast activity when Dlk2 was deleted in osteoclasts. Interestingly, although the serum P1NP level in Ctsk-Cre^+^;Dlk2^fl/fl^ mice was higher than that of WT littermates (Fig. 1J), the Ctsk-Cre^+^;Dlk2^fl/fl^ mice and WT mice exhibited a similar mineral apposition rate (MAR) and bone formation rate (BFR/BS) (Fig. 1G, H). In addition, no differences in the osteoblast-specific markers *Alp*, *Runx2* and *Col1a1* or the osteocyte markers *Sost* and *Dmp1* were observed in Ctsk-Cre^+^;Dlk2^fl/fl^ and WT mice (Fig. S2A), implying that the increase in bone mass was not caused by osteoblast-related bone formation. Overall, these results indicated that the Ctsk-Cre^+^;Dlk2^fl/fl^ mice developed a high-bone-mass phenotype, mainly due to decreased osteoclast formation and osteoclastic bone resorption.

### LysM-Cre^+^;Dlk2^fl/fl^ mice developed a high-bone-mass phenotype

**Fig. 2:**
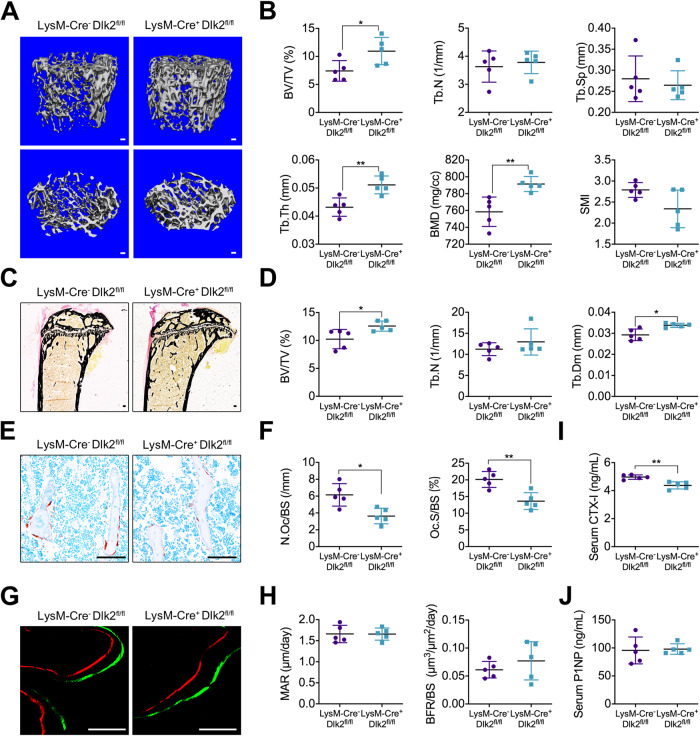
LysM-Cre^+^;Dlk2^fl/fl^ mice acquire a high-bone-mass phenotype. **A** Representative 3D micro-CT reconstruction images of distal femurs from 14-week-old LysM-Cre^-^;Dlk2^fl/fl^ (WT) and LysM-Cre^+^;Dlk2^fl/fl^ male mice. **B** Quantitative micro-CT analysis of the images shown in **A**. **C** Von Kossa staining of undecalcified tibial sections from 14-week-old WT and LysM-Cre^+^;Dlk2^fl/fl^ male mice. **D** Bone histomorphometric analysis of the Von Kossa-stained sections shown in **C**. **E** TRAP staining of undecalcified tibial sections from 14-week-old WT and LysM-Cre^+^;Dlk2^fl/fl^ male mice. **F** Bone histomorphometric analysis of the TRAP-stained sections shown in **E**. **G** Double label staining with calcein and alizarin red of undecalcified tibial sections from 14-week-old WT and LysM-Cre^+^;Dlk2^fl/fl^ male mice. **H** Bone histomorphometric analysis of double label stained sections shown in **G**. **I** Serum levels of CTX-I. **J** Serum levels of P1NP. (Scale bar, 100 μm, mean ± SD, Student’s tests, *p < 0.05, **p < 0.01, *n* = 5 for each genotype).

To further investigate the role of Dlk2 in osteoclast precursors, we generate LysM-Cre^+^;Dlk2^fl/fl^ mice. Fourteen-week-old LysM-Cre^+^;Dlk2^fl/fl^ male mice and WT mice showed no obvious differences in gross morphological changes. In a finding similar to that for Ctsk-Cre^+^;Dlk2^fl/fl^ mice, micro-CT analysis revealed that the skeletal mass of distal femurs of LysM-Cre^+^;Dlk2^fl/fl^ mice increased at 14 weeks of age (*n* = 5) (Fig. 2A). The BV/TV ratio, BMD and trabecular bone thickness (Tb.Th) of LysM-Cre^+^;Dlk2^fl/fl^ mice were significantly higher than those of WT mice (Fig. 2B). Bone histomorphometric analysis confirmed that LysM-Cre^+^;Dlk2^fl/fl^ mice exhibited a higher BV/TV ratio and greater trabecular bone diameter (Tb.Dm) than WT mice (Fig. 2C, D), implying high-bone-mass phenotype acquisition by LysM-Cre^+^;Dlk2^fl/fl^ mice. The lumber 3 vertebrae in LysM-Cre^+^;Dlk2^fl/fl^ mice also exhibited an increase in bone mass (Fig. S1H, I) Furthermore, osteoclast activity was significantly reduced in LysM-Cre^+^;Dlk2^fl/fl^ mice, which exhibited lower N.Oc/BS and Oc.S/BS ratios in TRAP-stained tibias (Fig. 2E, F) and lower expression of *Trap* and *Nfatc1* in bone tissues (Fig. S2B). The serum CTX-I level in LysM-Cre^+^;Dlk2^fl/fl^ mice was significantly reduced (Fig. 2I). In addition, the LysM-Cre^+^;Dlk2^fl/fl^ mice and WT mice exhibited similar MAR, BFR/BS ratios and serum P1NP levels (Fig. 2G, H and J). Hence, the results indicated that the LysM-Cre^+^;Dlk2^fl/fl^ mice developed a high-bone-mass phenotype, and Dlk2 deletion suppressed osteoclastic differentiation and led to higher bone density.

### Conditional deletion of Dlk2 exhibited high bone mass in OVX condition

**Fig. 3:**
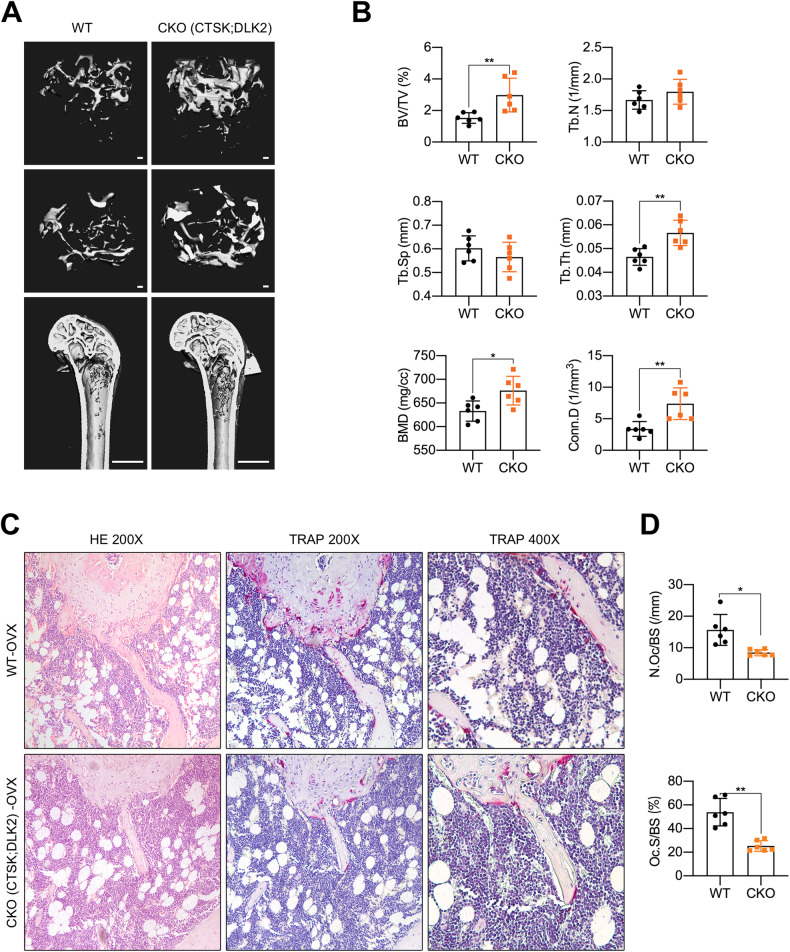
Conditional deletion of Dlk2 exhibits high bone mass in OVX condition. **A** Representative 3D micro-CT reconstruction images of distal femurs from 20-week-old Ctsk-Cre^-^;Dlk2^fl/fl^ (WT) and Ctsk-Cre^+^;Dlk2^fl/fl^ (CKO) female mice. Twelve-week-old WT and CKO female mice underwent ovariectomy (OVX) and were sacrificed after 2 months. **B** Quantitative micro-CT analysis of images shown in **A**; Conn.D connectivity density. **C** Representative images of H&E- and TRAP-stained sections from WT and CKO ovariectomized mice. The femurs were decalcified, embedded in paraffin and stained with H&E and TRAP. **D** Bone histomorphometric analysis of the TRAP-stained sections shown in **C**. (Scale bar, 100 μm, mean ± SD, Student’s tests, *p < 0.05, **p < 0.01, *n* = 6 for each genotype).

Deficient Dlk2 levels in osteoclasts resulted in impaired osteoclastogenesis and bone resorption. Therefore, we sought to determine whether Dlk2 deficiency could prevent OVX-induced osteoporosis. Ctsk-Cre^+^;Dlk2^fl/fl^ female mice (CKO) and WT littermates were ovariectomized at 12 weeks of age and sacrificed at 20 weeks of age (*n* = 6). The femurs were isolated and analyzed by micro-CT as previously described. As expected, the reconstructed 3D images revealed that OVX-CKO mice exhibited higher bone mass than WT mice (Fig. 3A), as indicated by a higher BV/TV ratio, BMD and connectivity density (Conn.D) in the femurs of OVX-CKO mice (Fig. 3B). The Tb.N of OVX-CKO mice showed an increasing trend, but it was not significantly different from that of OVX-WT mice. The Tb.Th was significantly increased in OVX-CKO mice, while the Tb.Sp was similar (Fig. 3B). Moreover, we observed that OVX-CKO mice exhibited lower N.Oc/BS and Oc.S/BS ratios than WT littermates (Fig. 3C, D), suggesting attenuated osteoclast activity in OVX-CKO mice. Together, these findings indicated that Dlk2 deficiency in osteoclasts exhibited increased bone mass in ovariectomized mice.

### Prx1-Cre^+^;Dlk2^fl/fl^ mice developed a low-bone-mass phenotype

Given the data that osteoclast-lineage Dlk2-specific knockout mice exhibited both high bone mass and decreased bone resorption, we next generated Prx1-Cre^+^;Dlk2^fl/fl^ mice to study the potential effect of Dlk2 in BMSCs. The Prx1-Cre^+^;Dlk2^fl/fl^ male mice and WT littermates shared similar body weights and sizes at 8 weeks of age. As shown in Fig. S3A, B, the 3D micro-CT reconstructions of distal femurs revealed lower bone mass in the 8-week-old Prx1-Cre^+^;Dlk2^fl/fl^ mice (*n* = 5), which exhibiting reduced BV/TV ratio, Tb.Th and increased SMI. Bone histomorphometric analysis also revealed a decrease in the Tb. N and BV/TV ratio in Prx1-Cre^+^;Dlk2^fl/fl^ mice (Fig. S3C, D). Furthermore, TRAP staining of tibias in Prx1-Cre^+^;Dlk2^fl/fl^ mice revealed an increasing trend in N.Oc/BS and Oc.S/BS ratios (Fig. S3E, F). We also observed a significant increase in osteoclast-specific genes in the bone tissues of Prx1-Cre^+^;Dlk2^fl/fl^ mice, while a reduction in osteoblast genes, such as *Alp* and *Runx2* (Fig. S2C). Moreover, we observed that Prx1-Cre^+^;Dlk2^fl/fl^ mice showed a slower MAR (Fig. S3G, H) and lower serum P1NP levels (Fig. S3J), suggesting that deletion of Dlk2 in BMSCs contributed to the robust osteoclastic bone resorption and impaired bone formation. Together, these results showed that the Prx1-Cre^+^;Dlk2^fl/fl^ mice developed a low-bone-mass phenotype because of suppressed bone formation and enhanced osteoclastic bone resorption.

### Deletion of Dlk2 inhibited both osteoclastogenesis and osteogenesis in vitro

**Fig. 4:**
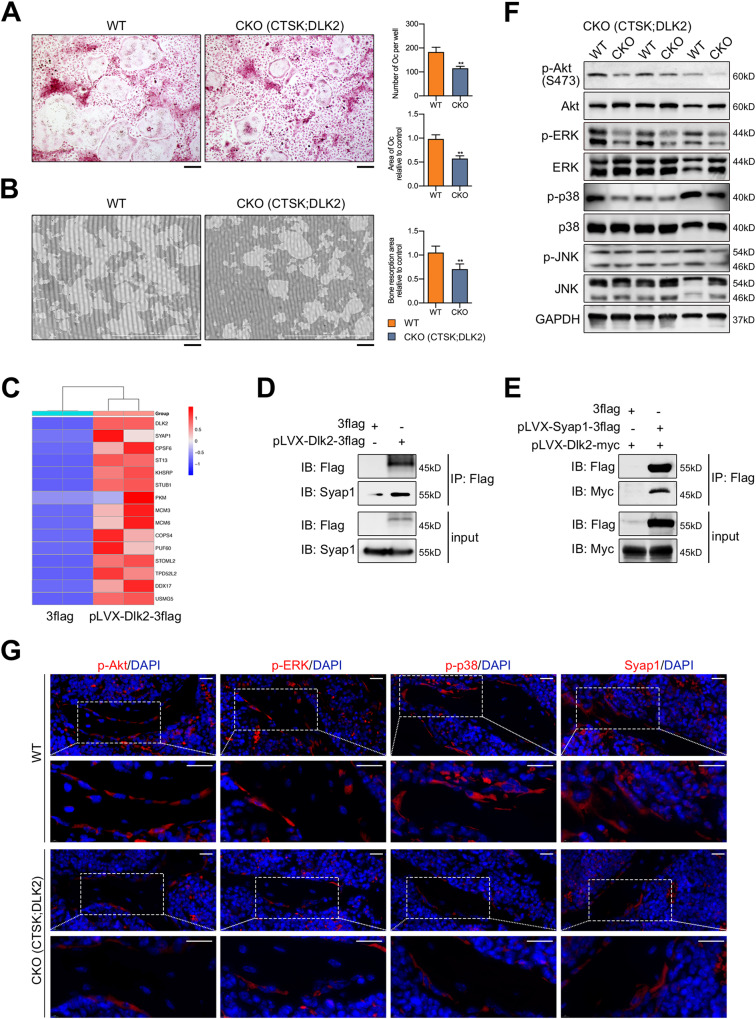
Deletion of Dlk2 inhibits osteoclastogenesis in vitro by inhibiting Akt/ERK/p38 and interacting with Syap1. **A** Representative images of TRAP-stained multinucleated osteoclasts. BMMs from Ctsk-Cre^-^;Dlk2^fl/fl^ (WT) and Ctsk-Cre^+^;Dlk2^fl/fl^ (CKO) male mice were stimulated with 30 ng/ml M-CSF and 50 ng/ml RANKL for 5 days, fixed in 4% paraformaldehyde and stained with TRAP solution. The number and size of the TRAP^+^ multinucleated osteoclasts were quantified (Scale bar, 200 μm, *n* = 3 independent samples). **B** Representative images of osteoclastic bone resorption. BMM-derived osteoclasts were cultured on bone-mimicking plates for 9 days and removed by 2% sodium hypochlorite. The bone resorption area shown was quantified (Scale bar, 200 μm, *n* = 3 independent samples). **C** The proteins that interact with Dlk2 were identified by coimmunoprecipitation (co-IP) combined with liquid chromatography-mass spectrometry (LC-MS/MS) analysis. HEK-293T cells were transfected with 10 μg pLVX-CMV-3flag (control) and pLVX-CMV-Dlk2-3flag (Dlk2-ov) plasmids using polyethyleneimine solution for 48 h. Then, the cells were harvested with IP lysis buffer for 30 min and incubated with anti-Flag M2 magnetic beads at 4 °C for 8 h for coimmunoprecipitation. The immunoprecipitated proteins were identified by LC-MS/MS analysis. The related heatmap shows the top 15 proteins that coimmunoprecipitated with significantly greater abundance in Dlk2-ov group. **D** Interaction of Dlk2 and Syap1 in HEK-293T cells. Cells were transfected with pLVX-CMV-3flag (control) and pLVX-CMV-Dlk2-3flag (Dlk2-ov) plasmids. All the cellular extracts were incubated with anti-Flag beads (IP: Flag) and immunoblotted with an antibody against Syap1 (*n* = 3 independent samples). **E** Interaction of Syap1 and Dlk2 in HEK-293T cells. Cells were cotransfected with pLVX-CMV-3flag and pLVX-CMV-Dlk2-myc (control), and pLVX-CMV-Syap1-3flag and pLVX-CMV-Dlk2-myc plasmids. All the cellular extracts were incubated with anti-Flag beads (IP: Flag) and immunoblotted with an antibody against Myc (*n* = 3 independent samples). **F** Western blot analysis was performed to detect total and phosphorylated forms of Akt, ERK, JNK, and p38 in Ctsk-Cre^-^;Dlk2^fl/fl^ (WT) and Ctsk-Cre^+^;Dlk2^fl/fl^ (CKO) osteoclasts induced by RANKL (*n* = 3 independent samples). **G** Representative images of immunofluorescence-stained femur sections from 8-week-old Ctsk-Cre^-^;Dlk2^fl/fl^ (WT) and Ctsk-Cre^+^;Dlk2^fl/fl^ (CKO) male mice. The sections were permeabilized with 0.1% Triton X-100, blocked with 5% goat serum, incubated with primary antibodies against p-Akt^Ser473^, p-ERK, p-p38 and Syap1 and then incubated with secondary antibodies. Nuclei were counterstained with DAPI (Scale bar, 20 μm, *n* = 3 independent samples). (Mean ± SD, Student’s tests, *p < 0.05, **p < 0.01).

BMMs were isolated from the tibias and femurs of Ctsk-Cre^+^;Dlk2^fl/fl^, LysM-Cre^+^;Dlk2^fl/fl^ and WT male mice for use in osteoclast formation assays. As shown in Fig. 4A, B; Fig. S4A, specific deletion of Dlk2 led to a reduction in mature osteoclast formation and bone resorption pits in Ctsk-Cre^+^;Dlk2^fl/fl^ group. Next, we observed a similar suppressive effect in LysM-Cre^+^;Dlk2^fl/fl^ osteoclasts (Fig. S4B–E). The expression of Dlk2 increased upon RANKL-induced osteoclast formation, and was inhibited in Ctsk-Cre^+^;Dlk2^fl/fl^ and LysM-Cre^+^;Dlk2^fl/fl^ osteoclasts (Fig. S4F, G). Moreover, we isolated BMMs from Dlk2^fl/fl^ and WT mice and transfected them with Cre adenovirus to observe any changes in osteoclastogenesis. As expected, Dlk2^fl/fl^-Cre BMMs showed inhibited osteoclastogenesis and a significant reduction in both the number and size of osteoclasts (Fig. S5). Therefore, Dlk2 deficiency in either early or late stage of osteoclast formation inhibited osteoclastogenic and bone resorptive function. The silencing of Dlk2 in BMMs significantly suppressed osteoclast differentiation and the expression of specific genes required for osteoclastogenesis, which further confirmed the inhibitory effect of Dlk2 deficiency on osteoclast (Fig. S6A–D and Fig. S7). In contrast, we transfected Dlk2-overexpressing (Dlk2-ov) lentivirus in BMMs. The results demonstrated that Dlk2 itself promoted osteoclastogenesis and osteoclastic gene expression (Fig. S8).

Furthermore, BMSCs obtained from Prx1-Cre^+^;Dlk2^fl/fl^ mice exhibited weaker osteogenic differentiation ability and lower expression levels of osteoblast-specific genes than WT group (Fig. S9A–C). According to western blot analysis, we found that the phosphorylation levels of Akt^Ser473^, ERK 1/2 and p38 were significantly inhibited in Dlk2-deficient osteoblasts (Fig. S9D, E). Thus, BMSC-specific deletion of Dlk2 led to attenuated osteogenesis.

### Dlk2 interacts with Syap1 to activate Akt signaling pathway

To identify whether Dlk2 interacts with other proteins or molecules, co-IP combined with LC-MS/MS was applied to predict the proteins physically associated with Dlk2. A total of 492 proteins in the control group and 682 proteins in the Dlk2-ov group were identified. We identified 205 proteins that were significantly differentially expressed between the two groups (with expression level differences greater than 1.5-fold with a *p* value less than 0.05), as determined by quantitative analysis performed with MaxQuant software (MaxQuant, Germany). The related heatmap showed the top 15 proteins that coimmunoprecipitated with significantly greater abundance in the Dlk2-ov group (Fig. 4C). Consistent with a previous study, the results showed that the most abundantly coimmunoprecipitated protein was Dlk2, suggesting that Dlk2 might interact with itself [34]. The second coimmunoprecipitated protein was Syap1, implying that Syap1 might interact with Dlk2 protein. Next, western blot analysis verified that overexpressed Dlk2 protein efficiently coimmunoprecipitated with endogenous Syap1 (Fig. 4D). Reciprocally, we confirmed a significant interaction between Syap1 and Dlk2 (Fig. 4E).

**Fig. 5:**
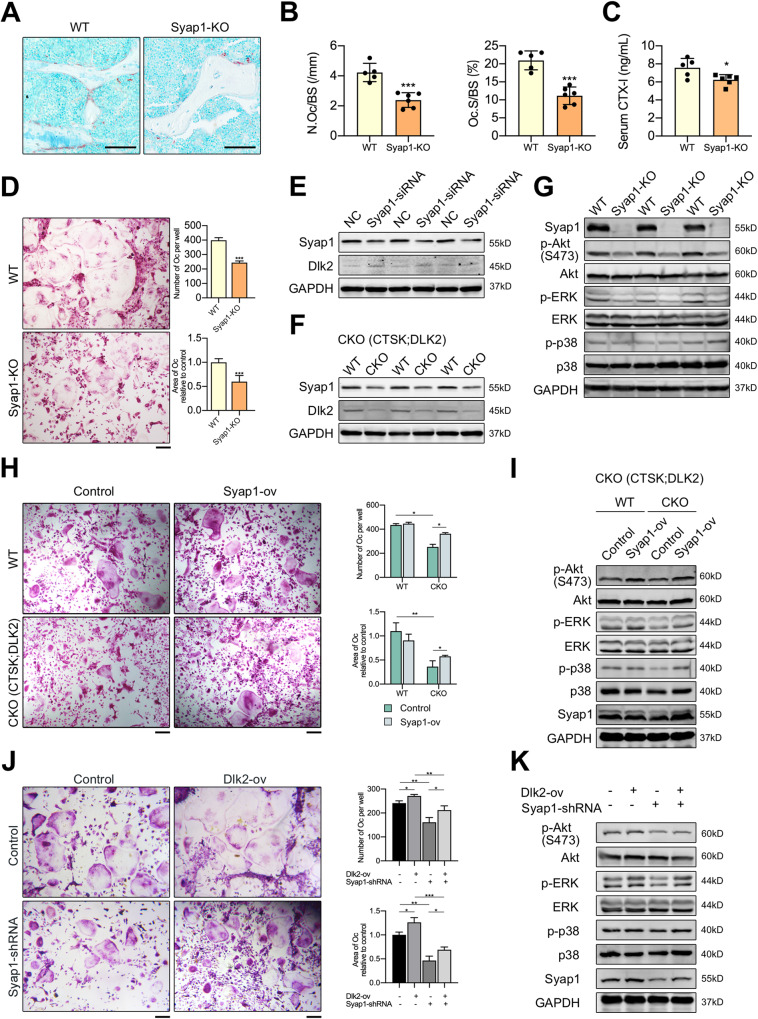
Dlk2 interacts with Syap1 to activate Akt signaling pathway. **A** TRAP staining of undecalcified tibial sections from 12-week-old WT and Syap1-KO male mice (Scale bar, 100 μm). **B** Bone histomorphometric analysis of the TRAP-stained sections shown in (A) (WT: *n* = 5, Syap1-KO: *n* = 6). **C** Serum levels of CTX-I (WT: *n* = 5, Syap1-KO: *n* = 6). **D** Representative images of TRAP-stained multinucleated osteoclasts. BMMs from WT and Syap1-KO male mice were stimulated with 30 ng/ml M-CSF and 50 ng/ml RANKL for 5 days. The number and size of the TRAP^+^ multinucleated osteoclasts were quantified (Scale bar, 200 μm, *n* = 3 independent samples). **E** Western blot analysis was performed to detect Syap1 and Dlk2 in NC and Syap1-siRNA osteoclasts (*n* = 3 independent samples). **F** Western blot analysis was performed to detect Syap1 and Dlk2 in Ctsk-Cre^-^;Dlk2^fl/fl^ (WT) and Ctsk-Cre^+^;Dlk2^fl/fl^ (CKO) osteoclasts (*n* = 3 independent samples). **G** Western blot analysis was performed to detect Syap1 and total and phosphorylated forms of Akt, ERK, and p38 in WT and Syap1-KO osteoclasts (*n* = 3 independent samples). **H** Representative images of TRAP-stained multinucleated osteoclasts. BMMs from Ctsk-Cre^-^;Dlk2^fl/fl^ (WT) and Ctsk-Cre^+^;Dlk2^fl/fl^ (CKO) male mice were transfected with Syap1-overexpressing lentivirus and stimulated with 30 ng/ml M-CSF and 50 ng/ml RANKL for 5 days. The number and size of the TRAP^+^ multinucleated osteoclasts in WT, WT-Syap1-ov, CKO and CKO-Syap1-ov groups were quantified (Scale bar, 200 μm, *n* = 3 independent samples). **I** Western blot analysis was performed to detect total and phosphorylated forms of Akt, ERK and p38 in **H** (*n* = 3 independent samples). **J** Representative images of TRAP-stained multinucleated osteoclasts. BMMs were transfected with NC lentivirus, Dlk2-overexpressing lentivirus, Syap1-shRNA lentivirus or Dlk2-ov + Syap1-shRNA lentivirus for 48 h and then stimulated with 30 ng/ml M-CSF and 50 ng/ml RANKL for 5 days. The number and size of the TRAP^+^ multinucleated osteoclasts were quantified (Scale bar, 200 μm, *n* = 3 independent samples). **K** Western blot analysis was performed to detect Syap1 and total and phosphorylated forms of Akt, ERK and p38. BMMs were transfected with NC lentivirus, Dlk2-overexpressing lentivirus, Syap1-shRNA lentivirus or Dlk2-ov + Syap1-shRNA lentivirus for 48 h. Then, BMMs were stimulated with 50 ng/ml RANKL for 10 min (*n* = 3 independent samples). (Mean ± SD, Student’s tests, *p < 0.05, **p < 0.01, ***p < 0.001).

Syap1 is also known as BSD domain-containing signal transducer and Akt interactor (BSTA) and has been detected in brain, heart, liver, skeletal muscles and adipose tissues. Syap1 interacts with Akt1 and can promote the phosphorylation of Akt^Ser473^ [35]. Syap1 was detected in BMMs and was continuously highly expressed during osteoclast formation (Fig. S1E). We next generated the Syap1-knockout male mice (denoted as Syap1-KO) to investigate the function of Syap1 in osteoclasts. TRAP staining of tibias revealed reduced N.Oc/BS and Oc.S/BS ratios in 12-week-old Syap1-KO male mice (*n* = 5) than in WT mice (*n* = 6) (Fig. 5A, B). The serum CTX-I level of Syap1-KO mice was significantly reduced (Fig. 5C). As shown in Fig. S10A, B, the micro-CT analysis of distal femurs revealed lower BMD of Syap1-KO male mice. The BV/TV ratio exhibited a decreasing trend in Syap1-KO mice with no significance. No difference in Tb.N, Tb.Th, Tb.Sp or SMI was observed. Bone histomorphometric analysis showed a decrease in the Tb. N and BV/TV ratio in Syap1-KO mice (Fig. S10C, D). Interestingly, Syap1-KO mice also showed a lower MAR and serum P1NP level (Fig. S10E–G). The expression of osteoclastic and osteoblastic genes exhibited a decreasing trend in the bone tissues of Syap1-KO mice with no significance (Fig. S10H). The in vitro results revealed that the Syap1-KO group exhibited weaker osteoclastic differentiation ability and osteogenic differentiation ability than that of the WT group (Fig. 5D, Fig. S11A–C).

The effect of Syap1 on osteoclasts was validated by Syap1 silencing in BMMs. Syap1 silencing had an effect on osteoclastogenesis similar to that of Dlk2 silencing, decreasing the number of osteoclasts and significantly reducing marker gene expression (Fig. S12A–C, Fig. S6A–D). Next, Akt^Ser473^ phosphorylation in Syap1-siRNA group was significantly decreased upon RANKL stimulation similar to Dlk2. The phosphorylation of ERK1/2, JNK and p38 was decreased by Syap1 silencing (Fig. S12D, E, Fig. S6E, F).

Furthermore, we found Dlk2 regulated Syap1 expression. The expression of Dlk2 proteins was increased slightly in Syap1-silenced BMMs (Fig. 5E). Dlk2 deletion in osteoclasts led to reduced Syap1 expression (Fig. 5F). Deletion of Dlk2 led to decreased phosphorylation levels of Akt^Ser473^, ERK1/2 and p38 in Dlk2- conditional knockout (Dlk2-CKO) osteoclasts (Fig. 4F), consistent with immunofluorescence staining (Fig. 4G). However, only the phosphorylation levels of Akt^Ser473^ and ERK1/2 were decreased in Syap1-deficient osteoclasts. No significant difference was observed in p38 phosphorylation (Fig. 5G). The changes in ERK1/2 phosphorylation were not as obvious as those in Akt^Ser473^ phosphorylation in either Syap1-knockout or Syap1-silenced osteoclasts (Fig. 5G, Fig. S12D). Therefore, given that Akt was a downstream target of Syap1 in a previous study, we investigated the role of the Dlk2-Syap1-Akt axis in osteoclastogenesis. We demonstrated that overexpression of Dlk2 activated the phosphorylation of Akt^Ser473^, ERK1/2 and p38 upon RANKL stimulation, while there was no significant increase in Syap1 expression (Fig. S13A). The addition of the Akt inhibitor LY294002 (10 μM) significantly reduced the phosphorylation level of Akt^Ser473^ in both control and Dlk2-ov groups, accompanied by decreased osteoclast formation (Fig. S13B, D). Similarly, overexpression of Syap1 activated the phosphorylation of Akt^Ser473^, ERK1/2 and p38 upon RANKL stimulation, while no difference in osteoclasts. LY294002 also inhibited the phosphorylation of Akt^Ser473^ and led to a defect in osteoclast differentiation in both control and Syap1-overexpressing groups (Fig. S13C, E). Importantly, TRAP staining indicated that overexpression of Syap1 in Dlk2-CKO osteoclasts rescued osteoclast differentiation (Fig. 5H, Fig. S14). Syap1 overexpression in Dlk2-CKO osteoclasts also rescued the phosphorylation levels of Akt^Ser473^, ERK1/2 and p38 proteins (Fig. 5I). Similarly, silencing of Syap1 in Dlk2-ov BMMs rescued the phosphorylation levels of Akt^Ser473^ and ERK1/2 proteins and osteoclast differentiation compared with those in Dlk2-ov group, while overexpression of Dlk2 in Syap1-shRNA BMMs rescued Akt^Ser473^ and ERK1/2 phosphorylation and osteoclast formation compared with those in Syap1-shRNA group (Fig. 5J, K).

Notch1 was previously reported as an interacting partner of Dlk2 [34]. Dlk2-deficient osteoclasts exhibited similar expression levels of Notch1, Hes1, Hes5 and Dlk1 proteins and mRNAs as WT controls (Fig. S15A, D). No obvious differences in the expression of Notch1, Hes1, Hes5 and Dlk1 were observed in Dlk2-silenced and negative controls or in Dlk2-ov and negative controls (Fig. S15B–F).

**Fig. 6:**
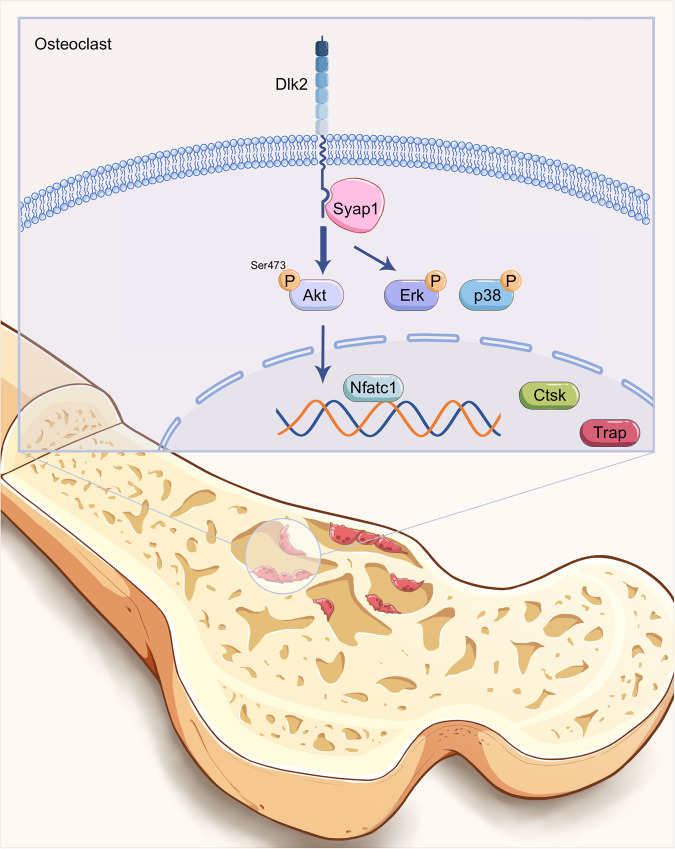
A schematic diagram of this study indicated that Dlk2-Syap1-Akt axis in osteoclastogenesis. Dlk2 deletion in osteoclasts suppressed osteoclastogenesis mainly by inhibiting Syap1 downstream cascades including the Akt^Ser473^, ERK1/2 and p38 phosphorylation.

Overall, our study demonstrates that Dlk2 regulates osteoclastogenesis by interacting with Syap1. Osteoclast-specific deletion of Dlk2 suppresses osteoclastogenesis by inhibiting the expression of Syap1 and activating the Akt^Ser473^, ERK1/2 and p38 signaling cascades (Fig. 6).

## Discussion

Bone disorders are usually caused by disrupted metabolism during bone formation and bone resorption. Our study revealed that Dlk2 exerted important effects on both osteoclastogenesis and osteogenesis. On the one hand, we reported a high-bone-mass phenotype in Ctsk-Cre^+^;Dlk2^fl/fl^ mice and LysM-Cre^+^;Dlk2^fl/fl^ mice with higher bone volume percentage and weakened osteoclast activity. Our in vitro experiments demonstrated that BMMs derived from Ctsk-Cre^+^;Dlk2^fl/fl^ mice and LysM-Cre^+^;Dlk2^fl/fl^ mice had difficulty forming classical mature osteoclasts. Silencing of Dlk2 by siRNA or shRNA lentivirus transfection and deletion of Dlk2 by Cre adenovirus transfection in BMMs also resulted in deficient osteoclast differentiation, while overexpression of Dlk2 promoted osteoclast differentiation and enhanced mRNA expression of osteoclastic marker genes. These results showed that Dlk2 promoted osteoclastogenesis and osteoclastic gene expression. On the other hand, we generated BMSC-specific Dlk2-knockout mice and reported a low-bone-mass phenotype in Prx1^+^;Dlk2^fl/fl^ mice. Our results showed inhibited bone formation and osteogenesis as well as active osteoclast resorption in Prx1^+^;Dlk2^fl/fl^ mice, contributing to the low bone mass. All these results indicate that Dlk2 is involved in both osteoclastogenesis and osteogenesis and that Dlk2 expression has potential effects on the bone remodeling process. Dlk2 deficiency leads to suppressed osteoclastogenesis and osteoclastic bone resorption.

Finally, we found a Dlk2 interactor and proposed that the Dlk2-Syap1-Akt signaling axis is activated during osteoclastogenesis. Dlk2 has been reported to interact extensively with itself to generate Dlk2 homodimers [34]. Dlk2 has also been shown to interact with Dlk1 [34], which is highly homologous to Dlk2, and to interact with Notch1 and inhibit the activation of Notch signaling in different cells [18, 34, 36]. Previous studies reported that Dlk1 positively regulated osteoclastogenesis [37], while Notch1 negatively regulated osteoclastogenesis [38]. Our results showed that changes in Dlk2 in RANKL-simulated osteoclasts had no effect on Dlk1 and Notch1 expression. This result suggested that Dlk2 regulated osteoclast formation is independent of Dlk1 and Notch1. Recently, Dlk2 was reported to physically interact with cMET and activate cMET downstream signaling pathways [15]. In this study, we performed co-IP combined with LC-MS/MS analysis and found an interaction between Dlk2 and Syap1 protein, an interactor of Akt [35]. Syap1 has been widely detected and functions by interacting with Akt1 and promoting the phosphorylation of Akt at Ser473 in adipose cells [35]. It has been established that PI3K/Akt signaling cascades are essential for osteoclast differentiation. Specifically, Akt induces osteoclastogenesis by promoting the phosphorylation of GSK3β, thereby promoting the nuclear translocation of NFATc1 [39, 40]. Many factors can affect this process. For example, conditional knockout of guanine nucleotide-binding protein subunit α13 (Gα13) was reported to enhance Akt/GSK3β/NFATc1 in RANKL-induced osteoclastogenesis and contribute to osteoporosis [41]. RANKL treatment facilitated the phosphorylation of GSK3β and induced its interaction and co-localization with heterogeneous nuclear ribonucleoprotein K (hnRNPK), leading to enhanced NFATc1 expression and osteoclast formation [42, 43]. Inhibition of Akt by PI3K inhibitors results in deficient osteoclast formation and protects mice from osteolytic bone loss [44, 45]. A previous study reported that Syap1 was induced by mTORC2 and facilitated the interaction between Syap1 and Akt1 [35]. The mTORC2 signaling is involved in regulating the cytoskeleton [46–48] through downstream targets, including Akt, PKC-α [49] and SGK1 [50]. It has been shown to regulate bone formation [51] and indirectly promote osteoclastogenesis [52]. In our study, the expression of the mTORC2 downstream targets SGK1 and PKCα was not influenced by Dlk2 expression, suggesting that Syap1-Akt contributed primarily to Dlk2-mediated osteoclastogenesis. Taken together, the results from our study reveal a Dlk2-Syap1-Akt signaling axis in Dlk2-mediated osteoclastogenesis.

Although we showed the inhibitory effects of Dlk2 deletion on osteoclast activities, some limitations to this study need to be acknowledged. The interaction sites between Syap1 and Dlk2 remain unidentified and might be essential for the regulation of the Dlk2-Syap1-Akt axis. Furthermore, we reported a low-bone-mass phenotype in Prx1-Cre^+^;Dlk2^fl/fl^ mice and found that Dlk2 was involved in osteogenesis. Although we found that the phosphorylation levels of Akt^Ser473^, ERK1/2 and p38 were inhibited in osteoblasts derived from Prx1-Cre^+^;Dlk2^fl/fl^ mice, the underlying mechanism of Dlk2-mediated bone formation remains unclear, and further investigation is needed.

In conclusion, our study revealed that Dlk2 deficiency in osteoclasts could suppress osteoclast differentiation and bone resorption both in vivo and in vitro by downregulating the expression of its binding protein Syap1 and inhibiting the phosphorylation-induced activation of Akt^Ser473^/MAPK (ERK1/2 and p38) cascades. A deficient Dlk2 level in osteoclasts effectively increased bone mass in ovariectomized mice, providing new insight into the prevention of osteoclast-related bone disorders.

## Supplementary information

Supplementary information

Original western blots

aj-checklist

## Data Availability

All data needed to evaluate the conclusions in the paper are present in the paper and/or the Supplementary Materials. The data that support the findings of this study are available from the corresponding author upon reasonable request.
