# Supplementary material for: Dlk2 interacts with Syap1 to activate Akt signaling pathway during osteoclast formation
Source: Cell Death Dis. 2023 Sep 5;14(9):589. doi: 10.1038/s41419-023-06107-1 (PMC10480461; doi:10.1038/s41419-023-06107-1)
Supplement: Supplementary file 1 — Supplementary information [file 41419_2023_6107_MOESM1_ESM.docx]

Supplementary information for

**Dlk2 interacts with Syap1 to activate Akt signaling pathway during osteoclast formation**

Xinwei Chen^1,#^, Xuzhuo Chen^1,#^, Rui Chao^1,#^, Yexin Wang^1^, Yi Mao^1^, Baoting Fan^1^, Yaosheng Zhang^2^, Weifeng Xu^1,*^, An Qin^3,*^, Shanyong Zhang^1,*^

1. Department of Oral and Maxillofacial Surgery, Shanghai Ninth People’s Hospital, Shanghai Jiao Tong University School of Medicine; College of Stomatology, Shanghai Jiao Tong University; National Center for Stomatology; National Clinical Research Center for Oral Diseases; Shanghai Key Laboratory of Stomatology, People's Republic of China.

2. Department of Stomatology, Shanghai Sixth People’s Hospital, Shanghai Jiao Tong University School of Medicine, People's Republic of China.

3. Department of Orthopaedics, Shanghai Ninth People’s Hospital, Shanghai Jiao Tong University School of Medicine, Shanghai Key Laboratory of Orthopaedic Implant, Shanghai, People’s Republic of China.

#These authors contributed equally to this work

*Co-corresponding authors

Correspondence to:

Weifeng Xu, E-mail: xwf19891215@163.com

An Qin, E-mail: dr_qinan@163.com

ShanYong Zhang, E-mail: [zhangshanyong@126.com](mailto:zhangshanyong@126.com)

**Table S1. The primary reagents used in this study.**

| **Reagents** | **Source** | **c C****Cat#** |
| --- | --- | --- |
| **Antibodies** | | |
| GAPDH | Cell Signaling Technology | #5714 |
| Dlk2 | Abcam | #ab171037 |
| pERK1/2 | Cell Signaling Technology | #4370 |
| ERK1/2 | Cell Signaling Technology | #4695 |
| p-JNK | Cell Signaling Technology | #4668 |
| JNK | Cell Signaling Technology | #9252 |
| p-p38 | Cell Signaling Technology | #4511 |
| p38 | Cell Signaling Technology | #8690 |
| p-GSK3β | Cell Signaling Technology | #5558 |
| GSK3β | Cell Signaling Technology | #12456 |
| p-Akt^Ser473^ | Cell Signaling Technology | #4060 |
| Akt | Cell Signaling Technology | #4691 |
| p-p65 | Cell Signaling Technology | #3033 |
| p65 | Cell Signaling Technology | #8242 |
| Syap1 | Proteintech | #16272-1-AP |
| Flag-tag (mouse) | Sigma Aldrich | #F3165 |
| Flag-tag (rabbit) | Cell Signaling Technology | #14793 |
| Myc-tag | Cell Signaling Technology | #2278 |
| SGK1 | Abcam | #ab32374 |
| PKC-α | Cell Signaling Technology | #2056 |
| Dlk1 | Proteintech | #10636-1-AP |
| Notch1 | Cell Signaling Technology | #3608 |
| Hes1 | Cell Signaling Technology | #11988 |
| Hes5 | Proteintech | #22666-1-AP |
| anti-rabbit IgG secondary antibody | Cell Signaling Technology | #5151 |
| anti-mouse IgG secondary antibody | LI-COR | #C50113-06 |
| Alexa Fluor 555-conjugated  secondary antibody | Invitrogen | #1937183 |
| **Chemicals** | | |
| Calcein | Sigma Aldrich | C0875 |
| Alizarin red | Sigma Aldrich | A5533 |
| TRAP solution | Sigma Aldrich | 387A |
| MEM-α | HyClone | SH30265.01B |
| High-glucose DMEM | HyClone | SH30022.01 |
| FBS | Nobimpex | A115-500 |
| penicillin/streptomycin | Gibco | 15070063 |
| M-CSF | R&D Systems | 416-ML-500 |
| RANKL | R&D Systems | 462-TEC-MTO |
| β-glycerophosphate | Sigma Aldrich | G9422 |
| ascorbic acid | Sigma Aldrich | A4403 |
| dexamethasone | Sigma Aldrich | D-1756 |
| cetylpyridinium chloride | Sigma Aldrich | C0732 |
| polyethyleneimine | Sigma Aldrich | 764604 |
| IP lysis buffer | Beyotime | P0013 |
| anti-Flag M2 magnetic beads | Sigma Aldrich | M8823 |
| SDS lysis buffer | Beyotime | P0013G |
| protease and phosphatase  inhibitor cocktail | Beyotime | P1045 |
| TRIzol Reagent | Takara Biotechnology | 9019 |
| DAPI | Invitrogen | #62247 |
| Cre adenovirus | GeneChem | GCD0177641 |
| **Kits** | | |
| Mouse CTX-I ELISA kit | Lengton | BPE20044 |
| Mouse P1NP ELISA kit | Lengton | BPE21100 |
| alkaline phosphatase staining kit | Beyotime | C3026 |
| PrimeScript RT reagent kit | Takara Biotechnology | R045Q |
| TB Green Premix Ex Taq kit | Takara Biotechnology | RR420A |

**Table S2. The sequences of quantitative real-time PCR primers used in this study.**

| **Name** | **Primer sequence** |
| --- | --- |
| Gapdh | Forward: 5’-GGTGAAGGTCGGTGTGAACG-3’ |
|  | Reverse: 5’-CTCGCTCCTGGAAGATGGTG-3’ |
| Dlk2 | Forward: 5’-GAACTTGTCCTACCTGCTCCA-3’ |
|  | Reverse: 5’-CTCCGTACCACCTCCTTCACT-3’ |
| Dlk2-null | Forward: 5'-GGCAGGAAAGTTCTGTGACAAAG-3'  Reverse: 5'-CAGGCACACACAGTGGTACTCA-3' |
| Trap | Forward: 5’-CAAAGAGATCGCCAGAACCG-3’ |
|  | Reverse: 5’-GAGACGTTGCCAAGGTGATC-3’ |
| Ctsk | Forward: 5’-CTTCCAATACGTGCAGCAGA-3’ |
|  | Reverse: 5’-TCTTCAGGGCTTTCTCGTTC-3’ |
| c-fos | Forward: 5’-CCAGTCAAGAGCATCAGCAA-3’ |
|  | Reverse: 5’-AAGTAGTGCAGCCCGGAGTA-3’ |
| Nfatc1 | Forward: 5’-CCGTTGCTTCCAGAAAATAACA-3’ |
|  | Reverse: 5’-TGTGGGATGTGAACTCGGAA-3’ |
| Atp6v0d2 | Forward: 5’-AAGCCTTTGTTTGACGCTGT-3’ |
|  | Reverse: 5’-TTCGATGCCTCTGTGAGATG-3’ |
| Ctr | Forward: 5’-TGCAGACAACTCTTGGTTGG-3’ |
|  | Reverse: 5’-TCGGTTTCTTCTCCTCTGGA-3’ |
| Dc-stamp | Forward:5ʹ‐TTGAACCGAGCTGCATTCCT‐3ʹ  Reverse: 5ʹ‐GTTTCCCGTCAGCCTCTCTC‐3' |
| Alp | Forward: 5’-AAATTCCCCTTTGTGGCCCT-3’ |
|  | Reverse: 5’-TAGTCACAATGCCCACGGAC-3’ |
| Runx2 | Forward: 5’-CCTCAGTGATTTAGGGCGCA-3’ |
|  | Reverse: 5’-GTGGTGGAGTGGATGGATGG-3’ |
| Col1a1 | Forward: 5’-CGACCTCAAGATGTGCCACT-3’ |
|  | Reverse: 5’-CCATCGGTCATGCTCTCTCC-3’ |
| Dmp1 | Forward: 5’-ACGGGTGATTTGGCTGGGTC-3’ |
|  | Reverse: 5’-GGGTATCTTGGGCACTGTTTTCT-3’ |
| Sost | Forward: 5’-TGCCTCATCTGCCTACTTGTG-3’ |
|  | Reverse: 5’-CGCCCGGTTCATGGTCT-3’ |
| Syap1 | Forward: 5'-GTGATGCCTTCGATACGTGC-3' |
|  | Reverse: 5'-TCTCGCTTCTCAGACTCTGC-3' |
| Notch1 | Forward: 5'-ATGGTGCTCTGATGGACGAC-3' |
|  | Reverse: 5'-ACTACTGGCTCCTCAAACCG-3' |
| Hes1 | Forward: 5'-TCTACACCAGCAACAGTGGG-3' |
|  | Reverse: 5'-AGTCCGAAGTGAGCGAGGA-3' |
| Hes5 | Forward: 5'-TGCTCAGTCCCAAGGAGAAA-3' |
|  | Reverse: 5'-CGAAGGCTTTGCTGTGTTT-3' |
| Dlk1 | Forward: 5'-CCTGGCTGTGTCAATGGAGT-3' |
|  | Reverse: 5'-GGAGCATTCGTACTGGCCTT-3' |
| Rankl | Forward: 5’-GCAGATTTGCAGGACTCGACT-3’ |
|  | Reverse: 5’-CCCCACAATGTGTTGCAGTT-3’ |
| Opg | Forward: 5’-ACCCAGAAACTGGTCATCAGC-3’ |
|  | Reverse: 5’-CTGCAATACACACACTCATCACT-3’ |

**Table S3. The siRNA and shRNA sequences used in this study.**

| **Name** | **siRNA** |
| --- | --- |
| siRNA-NC | 5'-TTCTCCGAACGTGTCACGT-3' |
| siRNA-Dlk2 | 5'-GCACATCTGTACCTCACAA-3' |
| siRNA-Syap1 | 5'-GCAACAGAGATGATAACTT-3' |
| NC-shRNA-F | 5'-gatctGTTCTCCGAACGTGTCACGTTTCAAGA  GAACGTGACACGTTCGGAGAATTTTTTc-3' |
| NC-shRNA-R | 5'-aattgAAAAAATTCTCCGAACGTGTCACGTTC  TCTTGAAACGTGACACGTTCGGAGAACa-3' |
| Syap1-shRNA-F | 5'-GATCCGCCCAGGAGTTTCTGAGTTTGCTCGAG  CAAACTCAGAAACTCCTGGGCTTTTTT-3' |
| Syap1-shRNA-R | 5'-AATTAAAAAAGCCCAGGAGTTTCTGAGTTTG  CTCGAGCAAACTCAGAAACTCCTGGGCG-3' |
| Dlk2-shRNA-F | 5'-CCGGAGTCTGGGCTTGGTGAATCTACTCGAG  TAGATTCACCAAGCCCAGACTTTTTTG-3' |
| Dlk2-shRNA-R | 5'-AATTCAAAAAAGTCTGGGCTTGGTGAATCTA  CTCGAGTAGATTCACCAAGCCCAGACT-3' |

**Table S4. The plasmids used in this study.**

| **Name** | **Plasmid information** | | |
| --- | --- | --- | --- |
| pLVX-CMV-Dlk2-3flag | 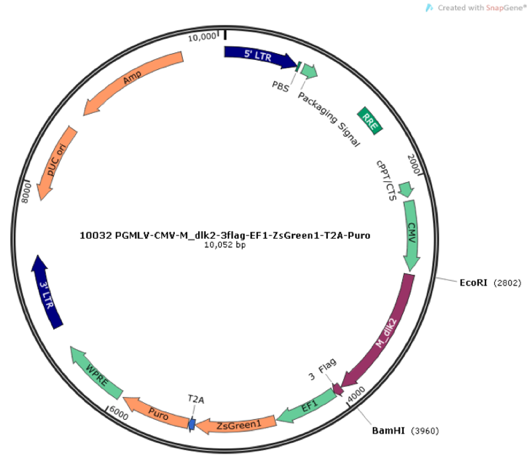 |  |  |
| pLVX-CMV-Dlk2-myc | 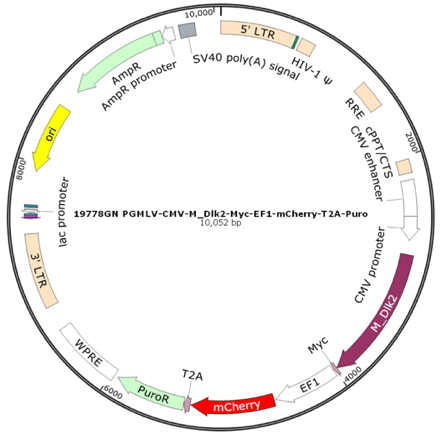 |  |  |
| pLVX-CMV-Syap1-3flag | **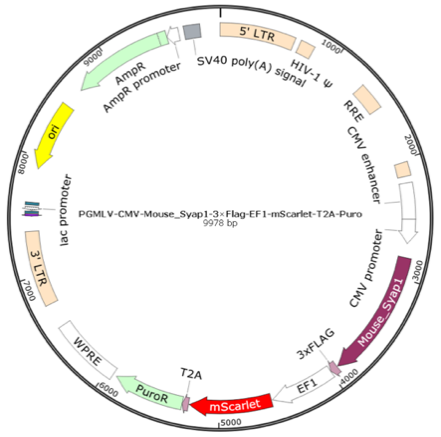** | | |
| pLVX-CMV-3flag | **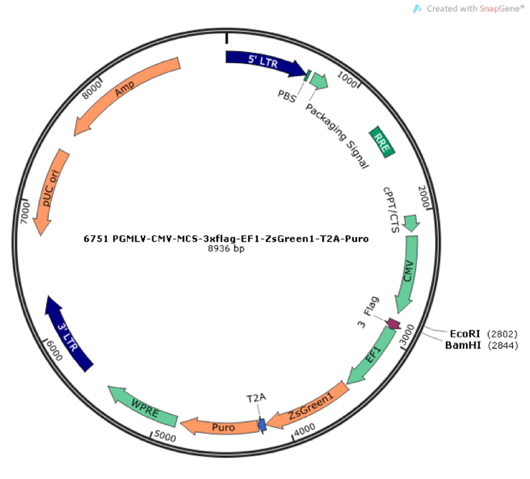** | | |


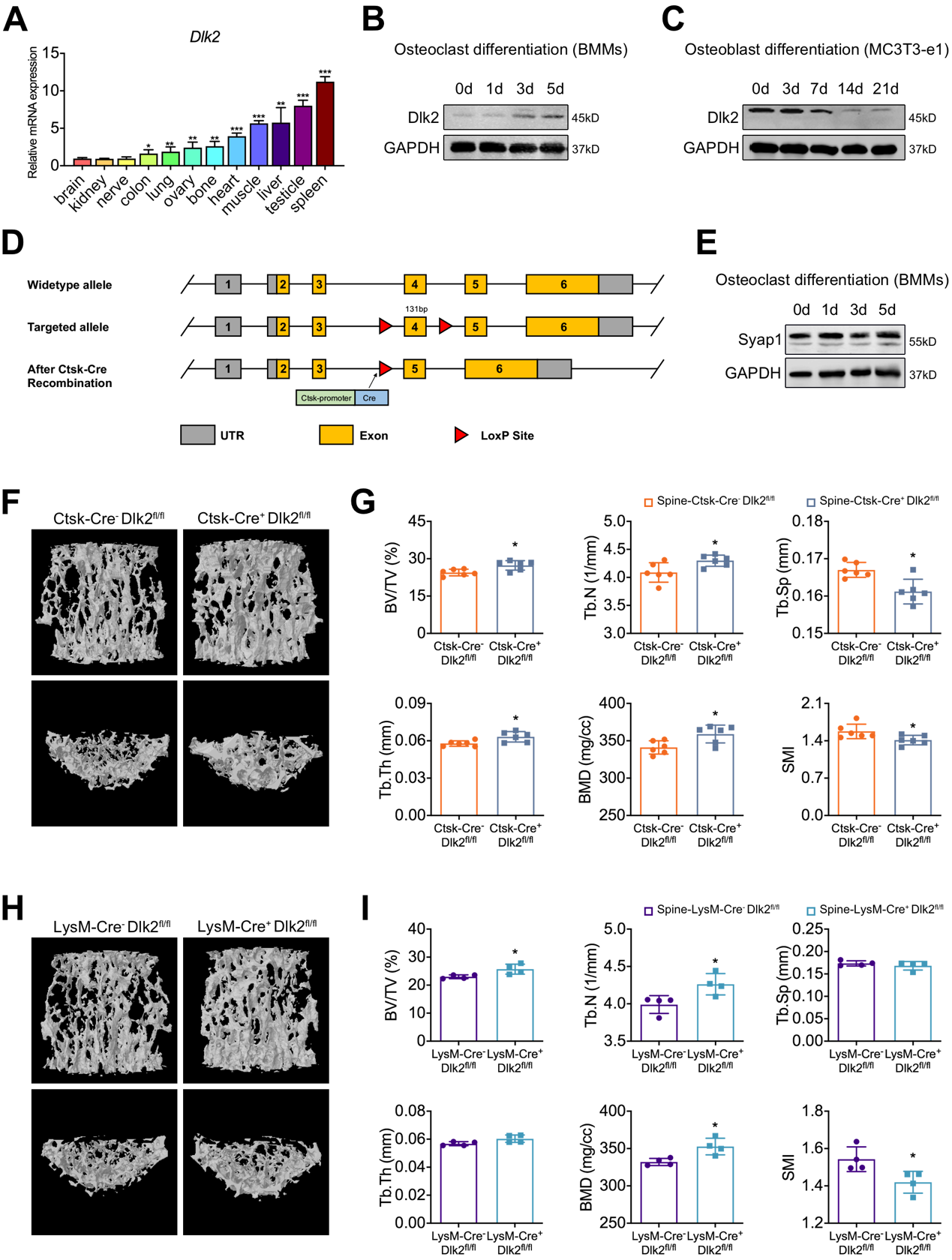


**Fig S1. The expression of Dlk2 and Syap1 and micro-CT analysis of mice spine.**

(A) Quantitative real-time PCR analysis was performed to detect the expression of Dlk2 in different mouse tissues. The results were normalized to the expression of Dlk2 in the brain (n=3 independent samples).

(B) Western blot analysis was performed to detect the Dlk2 protein levels in BMMs during RANKL-induced osteoclastogenesis (n=3 independent samples).

(C) Western blot analysis was performed to detect the Dlk2 protein levels in MC3T3-e1 cells during osteogenesis (n=3 independent samples).

(D) Strategy for generating Dlk2 conditional knockout mice.

(E) Western blot analysis was performed to detect Syap1 protein levels in BMMs during RANKL-induced osteoclastogenesis (n=3 independent samples).

(F) Representative 3D micro-CT reconstruction images of lumber 3 vertebrae from 8-week-old Ctsk-Cre^-^;Dlk2^fl/fl^ (WT) and Ctsk-Cre^+^;Dlk2^fl/fl^ male mice.

(G) Quantitative micro-CT analysis of the images shown in (F); BT/TV, bone volume percentage/tissue volume ratio; Tb.N, trabecular bone number; Tb.Sp, trabecular bone separation; Tb.Th, trabecular bone thickness; BMD, bone mineral density; SMI, structure model index (n=6 for each genotype).

(H) Representative 3D micro-CT reconstruction images of lumber 3 vertebrae from 8-week-old LysM-Cre^-^;Dlk2^fl/fl^ (WT) and LysM-Cre^+^;Dlk2^fl/fl^ male mice.

(I) Quantitative micro-CT analysis of the images shown in (H) (n=4 for each genotype).

(Mean±SD, Student's tests, * *p* < 0.05, ** *p* < 0.01, *** *p* <0.001)

**
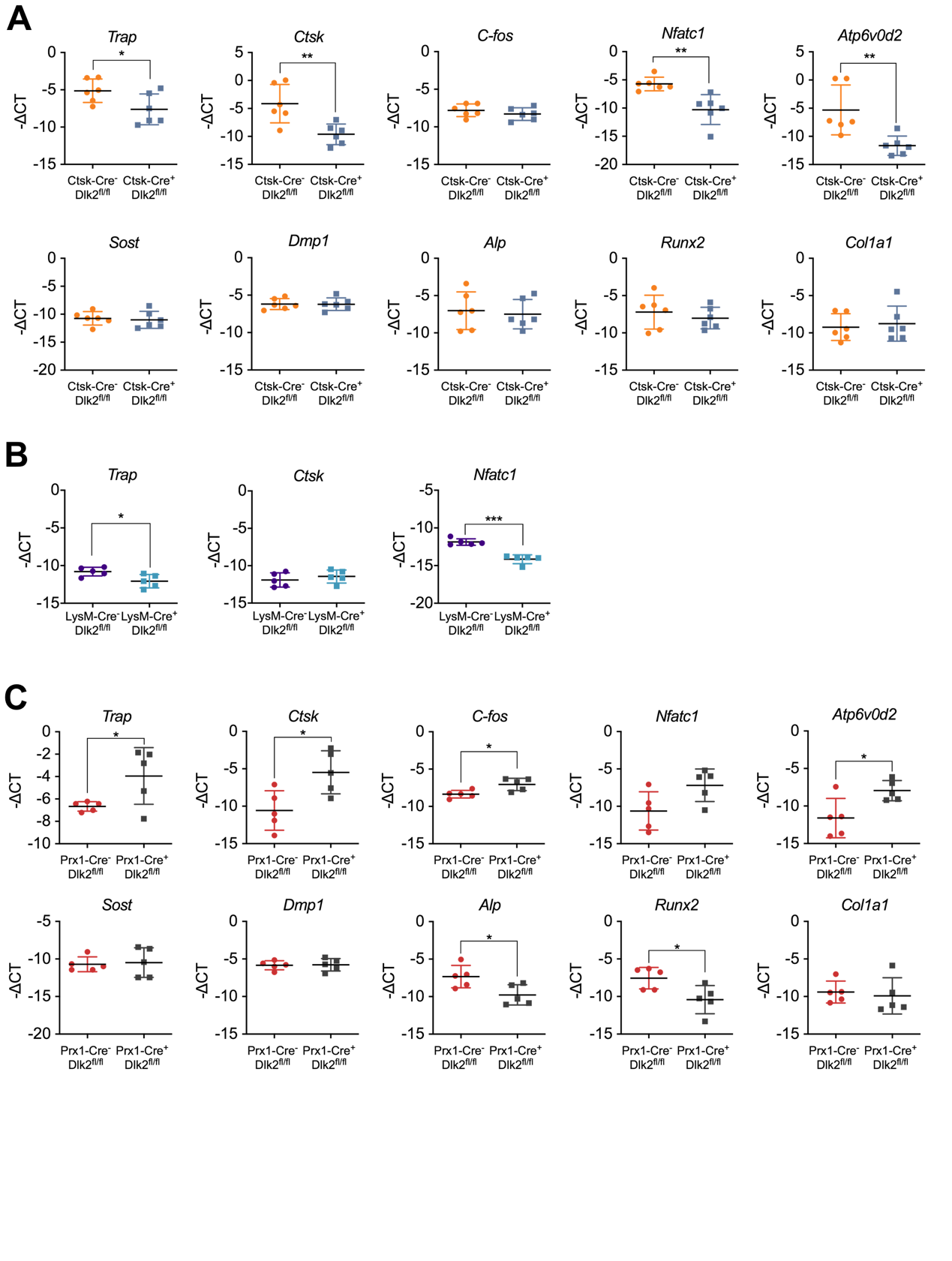
**

**Fig S2. The expression of marker genes in bone tissues of different mice.**

(A) Quantitative real-time PCR analysis was performed to detect the expression of osteoclast marker genes (*Trap*, *Ctsk*, *c-fos*, *Nfatc1* and *Atp6v0d2*), osteocyte marker genes (*Sost* and *Dmp1)*, and osteoblast marker genes (*Alp*, *Runx2* and *Col1a1*) in bone tissues of 8-week-old Ctsk-Cre^-^;Dlk2^fl/fl^ and Ctsk-Cre^+^;Dlk2^fl/fl^ male mice (n=6 for each genotype).

(B) Quantitative real-time PCR analysis was performed to detect the expression of *Trap*, *Ctsk* and *Nfatc1* in bone tissues of 8-week-old LysM-Cre^-^;Dlk2^fl/fl^ and LysM-Cre^+^;Dlk2^fl/fl^ male mice (n=5 for each genotype).

(C) Quantitative real-time PCR analysis was performed to detect the expression of *Trap*, *Ctsk*, *c-fos*, *Nfatc1*, *Atp6v0d2*, *Sost*, *Dmp1*, *Alp*, *Runx2* and *Col1a1* in bone tissues of 8-week-old Prx1-Cre^-^;Dlk2^fl/fl^ and Prx1-Cre^+^;Dlk2^fl/fl^ male mice. The results are presented as -△CT and normalized to the expression of the housekeeping gene GAPDH (n=5 for each genotype).

(Mean±SD, Student's tests, * *p* < 0.05, ** *p* < 0.01, *** *p* <0.001)

**
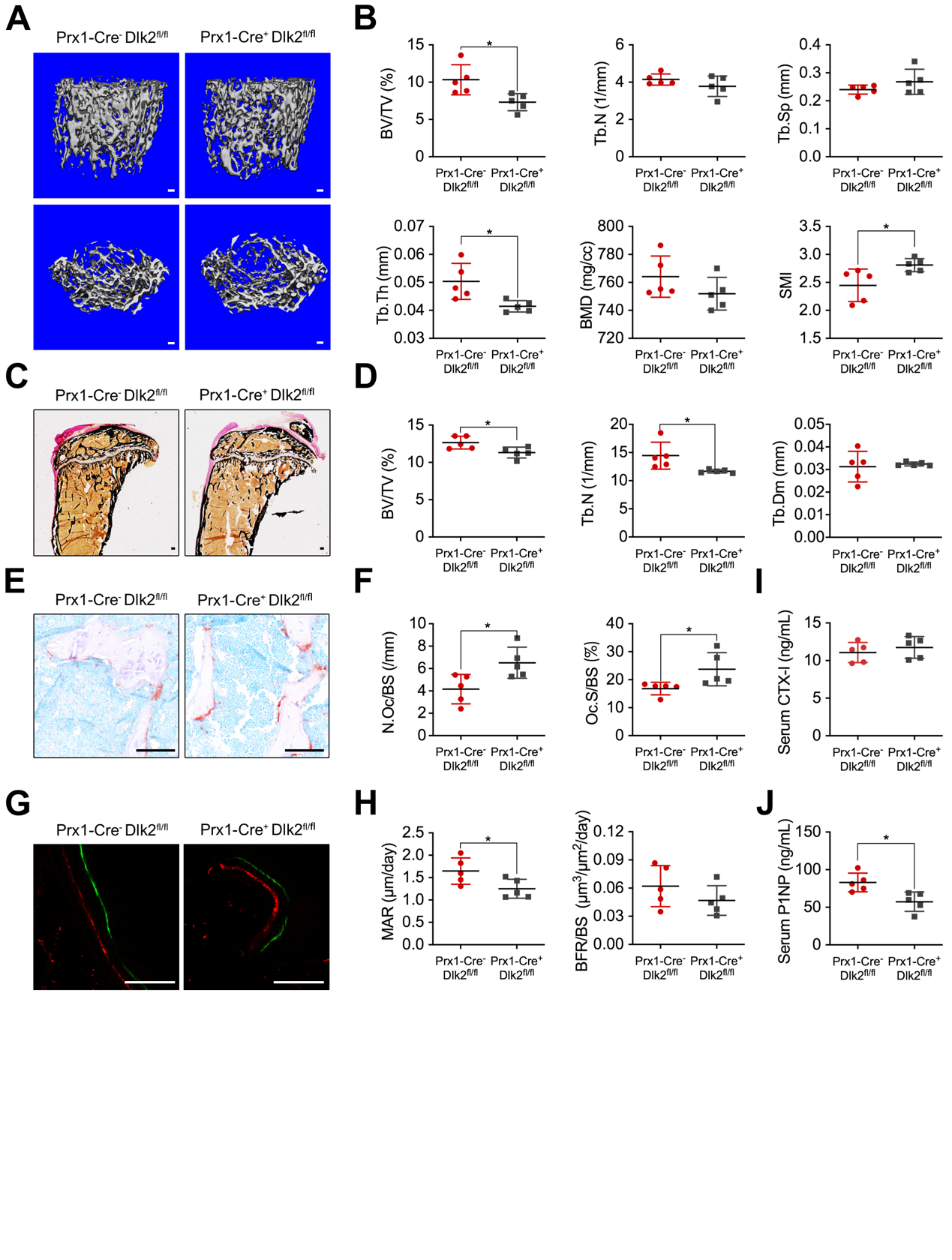
**

**Fig S3. Prx1-Cre^+^;Dlk2^fl/fl^ mice acquire a low-bone-mass phenotype.**

(A) Representative 3D micro-CT reconstruction images of distal femurs from 8-week-old Prx1-Cre^-^;Dlk2^fl/fl^ (WT) and Prx1-Cre^+^;Dlk2^fl/fl^ male mice.

(B) Quantitative micro-CT analysis of the images shown in (A).

(C) Von Kossa staining of undecalcified tibial sections from 8-week-old WT and Prx1-Cre^+^;Dlk2^fl/fl^ male mice.

(D) Bone histomorphometric analysis of the Von Kossa-stained sections shown in (C).

(E) TRAP staining of undecalcified tibial sections from 8-week-old WT and Prx1-Cre^+^;Dlk2^fl/fl^ male mice.

(F) Bone histomorphometric analysis of the TRAP-stained sections shown in (E).

(G) Double label staining with calcein and alizarin red of undecalcified tibial sections from 8-week-old WT and Prx1-Cre^+^;Dlk2^fl/fl^ male mice.

(H) Bone histomorphometric analysis of the double label stained sections shown in (G).

(I) Serum levels of CTX-I.

(J) Serum levels of P1NP.

(Scale bar, 100 μm, mean±SD, Student's tests, * *p* < 0.05, ** *p* < 0.01, n=5 for each genotype)

**
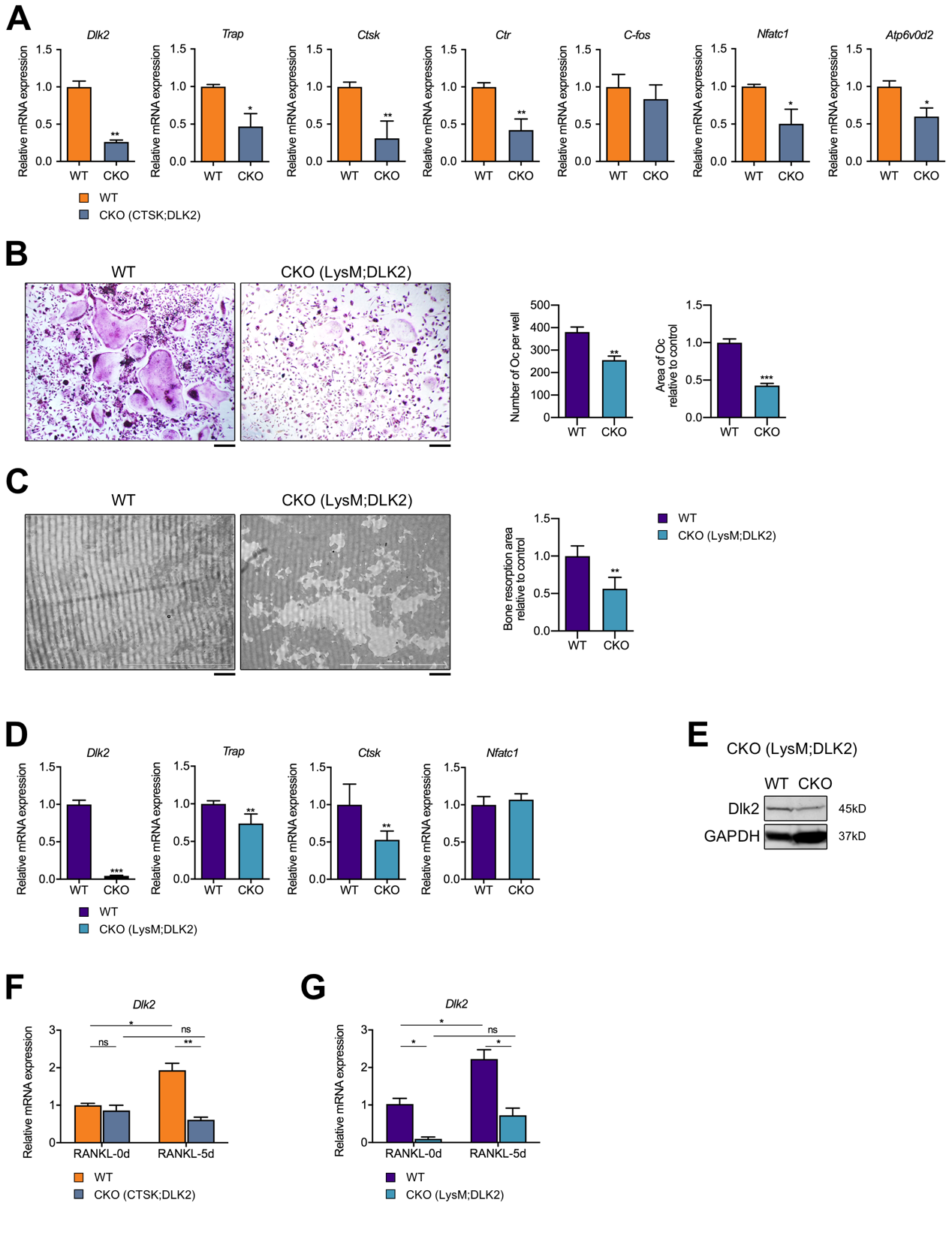
**

**Fig S4. Deletion of Dlk2 inhibits osteoclastogenesis *in vitr*o.**

(A) Quantitative real-time PCR analysis was performed to detect the expression of *Dlk2*, *Trap*, *Ctsk*, *Ctr*, *c-fos*, *Nfatc1* and *Atp6v0d2* in Ctsk-Cre^-^;Dlk2^fl/fl^ (WT) and Ctsk-Cre^+^;Dlk2^fl/fl^ (CKO) osteoclasts induced by RANKL.

(B) Representative images of TRAP-stained multinucleated osteoclasts from LysM-Cre^-^;Dlk2^fl/fl^ (WT) and LysM-Cre^+^;Dlk2^fl/fl^ (CKO) male mice. The number and size of the TRAP^+^ multinucleated osteoclasts were quantified.

(C) Representative images of osteoclastic bone resorption from LysM-Cre^-^;Dlk2^fl/fl^ (WT) and LysM-Cre^+^;Dlk2^fl/fl^ (CKO) male mice. The bone resorption area shown was quantified.

(D) Quantitative real-time PCR analysis was performed to detect the expression of *Dlk2*, *Trap*, *Ctsk*, and *Nfatc1* in LysM-Cre^-^;Dlk2^fl/fl^ (WT) and LysM-Cre^+^;Dlk2^fl/fl^ (CKO) osteoclasts induced by RANKL. The results normalized to the expression of the housekeeping gene GAPDH.

(E) Western blot analysis was performed to detect Dlk2 in LysM-Cre^-^;Dlk2^fl/fl^ (WT) and LysM-Cre^+^;Dlk2^fl/fl^ (CKO) osteoclasts.

(F) Quantitative real-time PCR analysis was performed to detect the expression of *Dlk2* in Ctsk-Cre^-^;Dlk2^fl/fl^ (WT) and Ctsk-Cre^+^;Dlk2^fl/fl^ (CKO) osteoclasts during RANKL-induced osteoclastogenesis.

(G) Quantitative real-time PCR analysis was performed to detect the expression of *Dlk2* in LysM-Cre^-^;Dlk2^fl/fl^ (WT) and LysM-Cre^+^;Dlk2^fl/fl^ (CKO) osteoclasts during RANKL-induced osteoclastogenesis.

(Scale bar, 200 μm, mean±SD, Student's tests, * *p* < 0.05, ** *p* < 0.01, *** *p* <0.001, n=3 independent samples. NS, not significant)

**
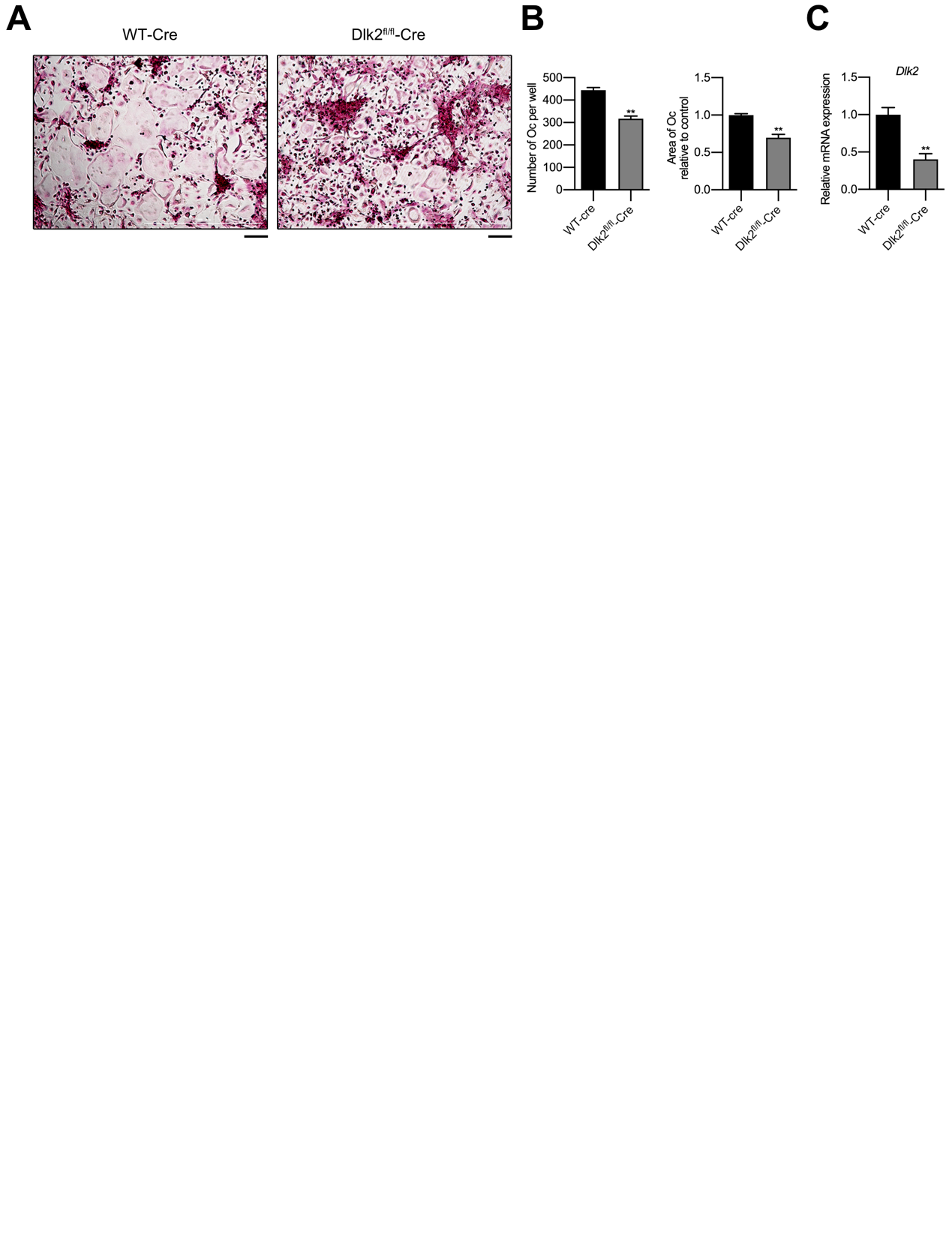
**

**Fig S5. Deletion of Dlk2 inhibits osteoclastogenesis *in vitr*o.**

(A) Representative images of TRAP-stained multinucleated osteoclasts. BMMs from WT and Dlk2^fl/f^ mice were transfected with Cre adenovirus for 48 hrs, and stimulated with 30 ng/ml M-CSF and 50 ng/ml RANKL for 5 days.

(B) The number and size of the TRAP^+^ multinucleated osteoclasts shown in (A) were quantified.

(C) Quantitative real-time PCR analysis was performed to detect the expression of *Dlk2* in WT-Cre and Dlk2^fl/fl^-Cre groups. The results are normalized to the expression of the housekeeping gene GAPDH.

(Scale bar, 200 μm, mean±SD, Student's tests, * *p* < 0.05, ** *p* < 0.01, n=3 independent samples)

**
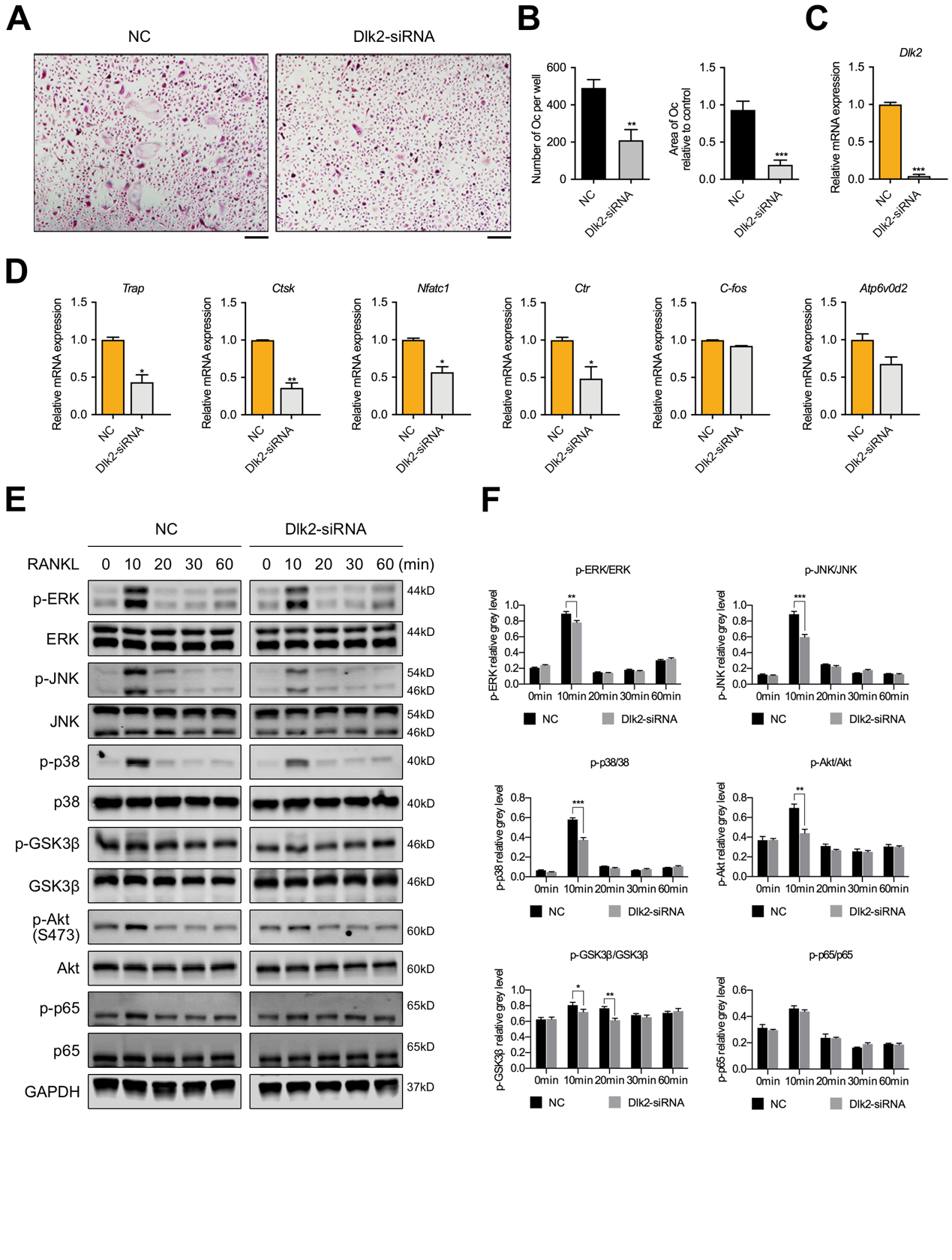
**

**Fig S6. Silencing of Dlk2 inhibits RANKL-induced osteoclastogenesis by suppressing PI3K/Akt and MAPK signaling pathways.**

(A) Representative images of TRAP-stained multinucleated osteoclasts. BMMs were transfected with NC-siRNA and Dlk2-siRNA for 48 hrs, and stimulated with 30 ng/ml M-CSF and 50 ng/ml RANKL for 5 days.

(B) The number and size of the TRAP^+^ multinucleated osteoclasts shown in (A) were quantified.

(C) Quantitative real-time PCR analysis was performed to detect the expression of *Dlk2* in NC and Dlk2-siRNA groups.

(D) Quantitative real-time PCR analysis was performed to detect the expression of *Trap*, *Ctsk*, *Nfatc1*, *Ctr*, *c-fos* and *Atp6v0d2* in NC and Dlk2-siRNA groups. The results were normalized to the expression of the housekeeping gene GAPDH.

(E) Western blot analysis was performed to detect total and phosphorylated forms of ERK, JNK, p38, Akt, GSK3β and p65. BMMs were transfected with NC-siRNA and Dlk2-siRNA for 48 hrs followed by stimulation with 50 ng/ml RANKL for 10, 20, 30, and 60 mins.

(F) Quantitative densitometric analysis of ERK, JNK, p38, Akt, GSK3β and p65 phosphorylation normalized to total protein expression.

(Scale bar, 200 μm, mean±SD, Student's tests, * *p* < 0.05, ** *p* < 0.01, *** *p* <0.001, n=3 independent samples)

**
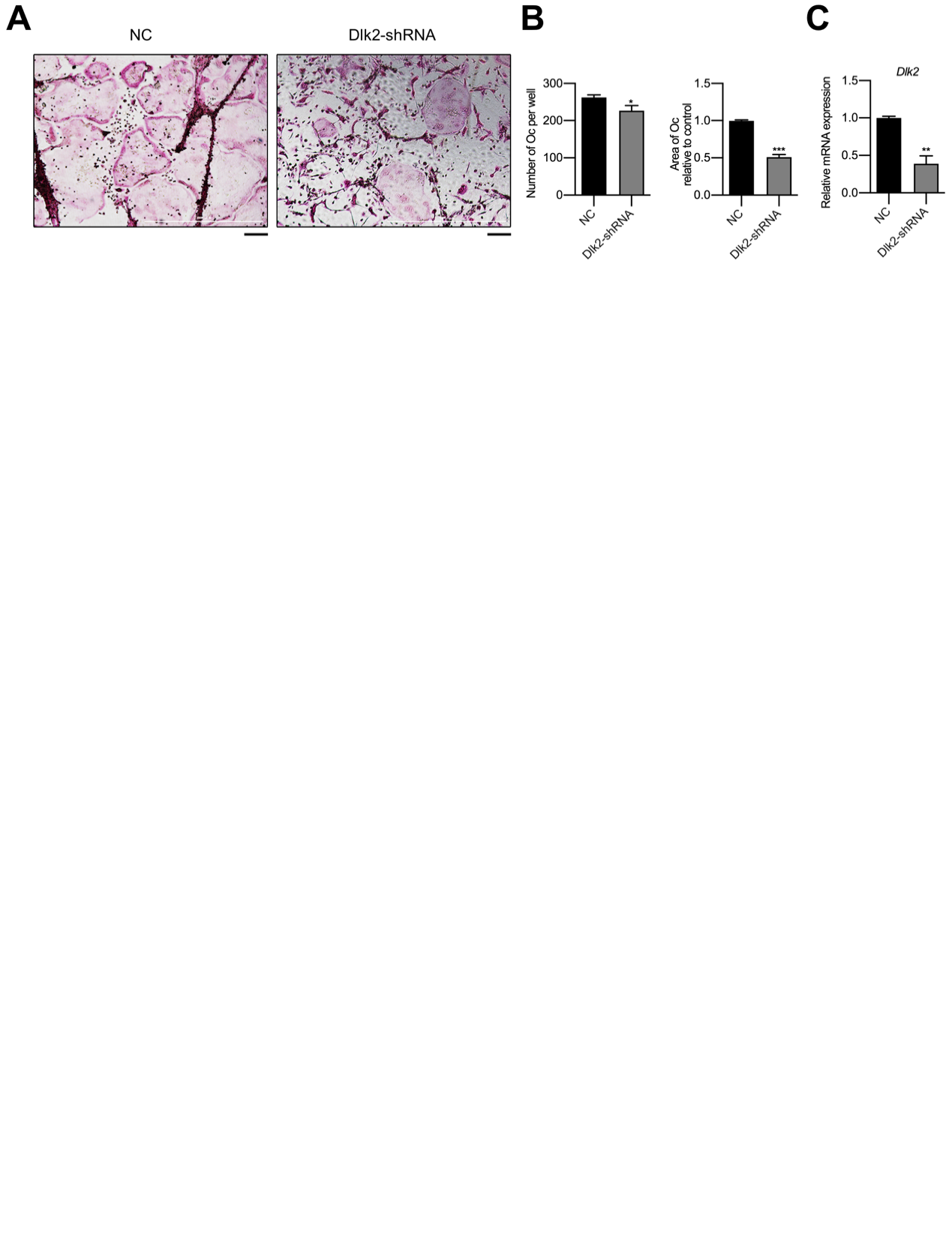
**

**Fig S7. Silencing of Dlk2 inhibits osteoclastogenesis.**

(A) Representative images of TRAP-stained multinucleated osteoclasts. BMMs were transfected with NC lentivirus and Dlk2-shRNA lentivirus for 48 hrs, and stimulated with 30 ng/ml M-CSF and 50 ng/ml RANKL for 5 days.

(B) The number and size of the TRAP^+^ multinucleated osteoclasts shown in (A) were quantified.

(C) Quantitative real-time PCR analysis was performed to detect the expression of *Dlk2* in NC and Dlk2-shRNA groups. The results are normalized to the expression of the housekeeping gene GAPDH.

(Scale bar, 200 μm, mean±SD, Student's tests, * *p* < 0.05, ** *p* < 0.01, *** *p* <0.001, n=3 independent samples)

**
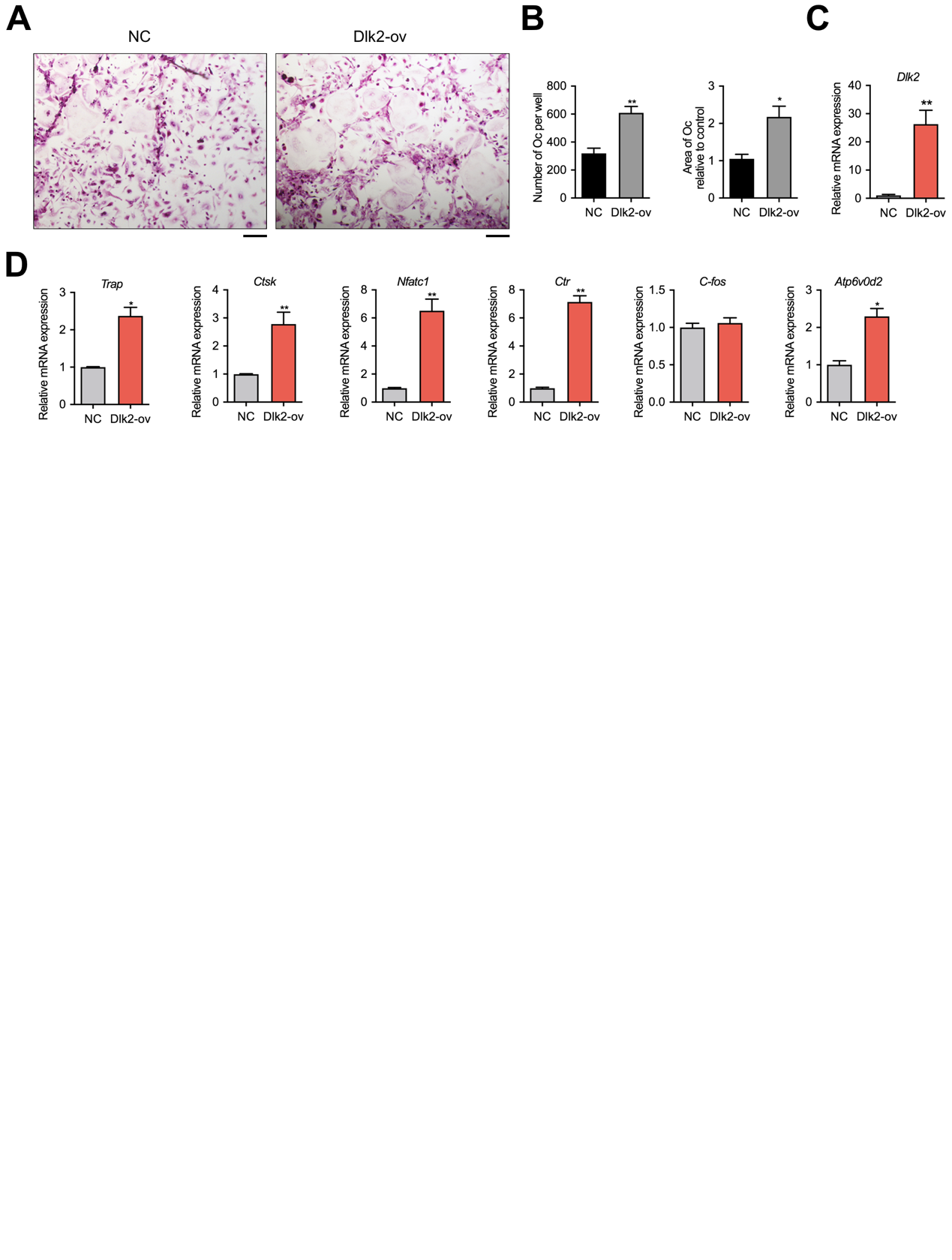
**

**Fig S8. Overexpression of Dlk2 promoted osteoclastogenesis.**

(A) Representative images of TRAP-stained multinucleated osteoclasts. BMMs were transfected with NC lentivirus and Dlk2-overexpressing lentivirus for 48 hrs, and stimulated with 30 ng/ml M-CSF and 50 ng/ml RANKL for 5 days.

(B) The number and size of the TRAP^+^ multinucleated osteoclasts shown in (A) were quantified.

(C) Quantitative real-time PCR analysis was performed to detect Dlk2 expression in NC and Dlk2-ov groups.

(D) Quantitative real-time PCR analysis was performed to detect the expression of *Trap*, *Ctsk*, *Nfatc1*, *Ctr*, *c-fos* and *Atp6v0d2* in NC and Dlk2-ov groups. The results were normalized to the expression of the housekeeping gene GAPDH.

(Scale bar, 200 μm, mean±SD, Student's tests, * *p* < 0.05, ** *p* < 0.01, n=3 independent samples)

**
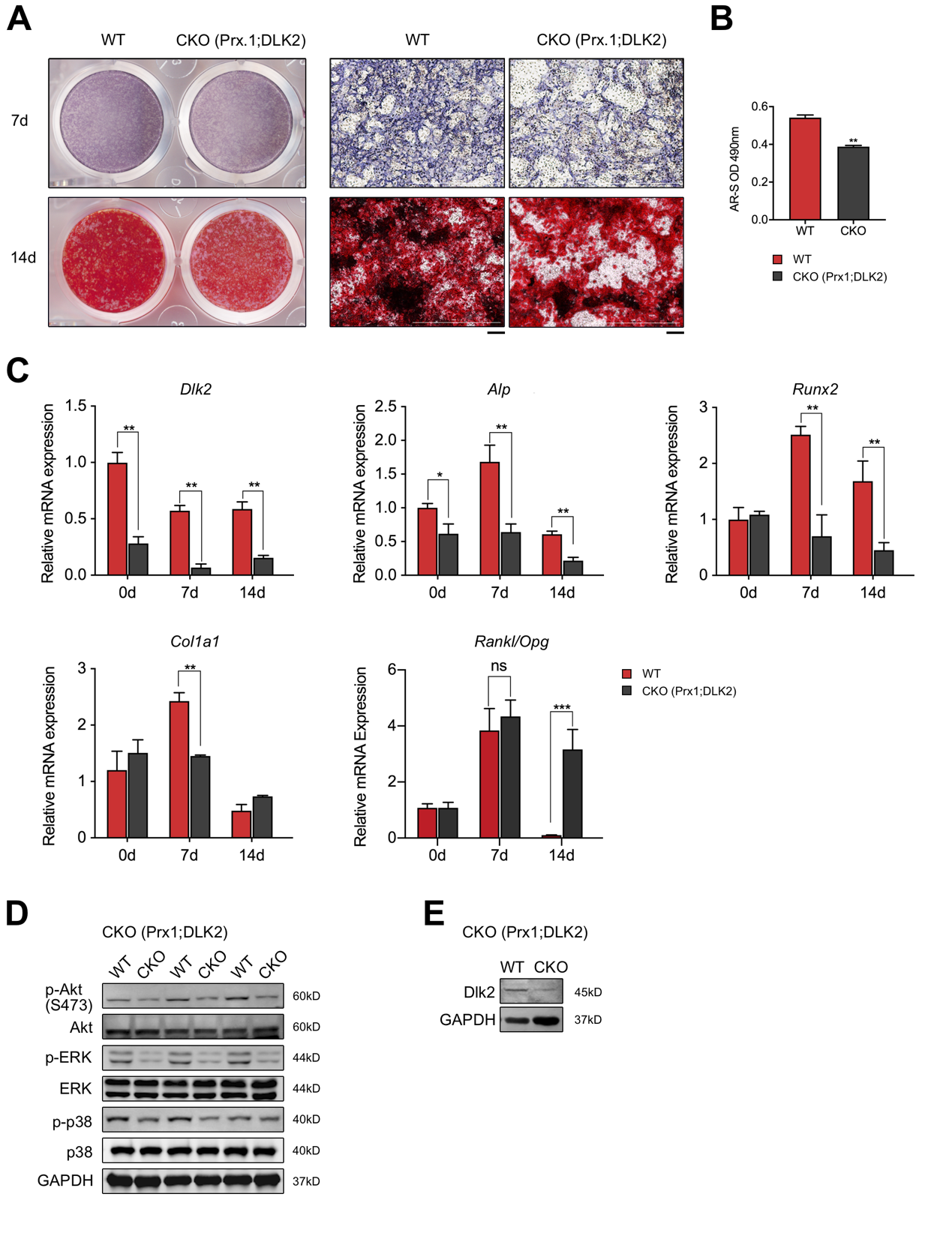
**

**Fig S9. Deletion of Dlk2 inhibits osteogenesis *in vitro*.**

(A) Representative images of alkaline phosphatase and alizarin red staining. BMSCs obtained from Prx1-Cre^-^;Dlk2^fl/fl^ (WT) and Prx1-Cre^+^;Dlk2^fl/fl^ (CKO) male mice were stimulated with 10 mM β-glycerophosphate, 10^-7^ mM dexamethasone and 50 μg/ml ascorbic acid for 14 days. The cells were fixed in 4% paraformaldehyde, stained with ALP solution on day 7, and stained with alizarin red solution on day 14.

(B) Quantitative analysis of the alizarin red-stained cells in (A).

(C) Quantitative real-time PCR analysis was performed to detect the expression of *Dlk2*, *Alp*, *Runx2*, and *Col1a1* and the *Rankl/Opg* ratio in 8-week-old Prx1-Cre^-^;Dlk2^fl/fl^ (WT) and Prx1-Cre^+^;Dlk2^fl/fl^ (CKO) osteoblasts. The results are normalized to the expression of the housekeeping gene GAPDH.

(D) Western blot analysis was performed to detect total and phosphorylated forms of Akt, ERK and p38 in osteoblasts derived from 8-week-old Prx1-Cre^-^;Dlk2^fl/fl^ (WT) and Prx1-Cre^+^;Dlk2^fl/fl^ (CKO) male mice.

(E) Western blot analysis was performed to detect Dlk2 in Prx1-Cre^-^;Dlk2^fl/fl^ (WT) and Prx1-Cre^+^;Dlk2^fl/fl^ (CKO) osteoblasts.

(Scale bar, 200 μm, mean±SD, Student's tests, * *p* < 0.05, ** *p* < 0.01, *** *p* <0.001, n=3 independent samples)

**
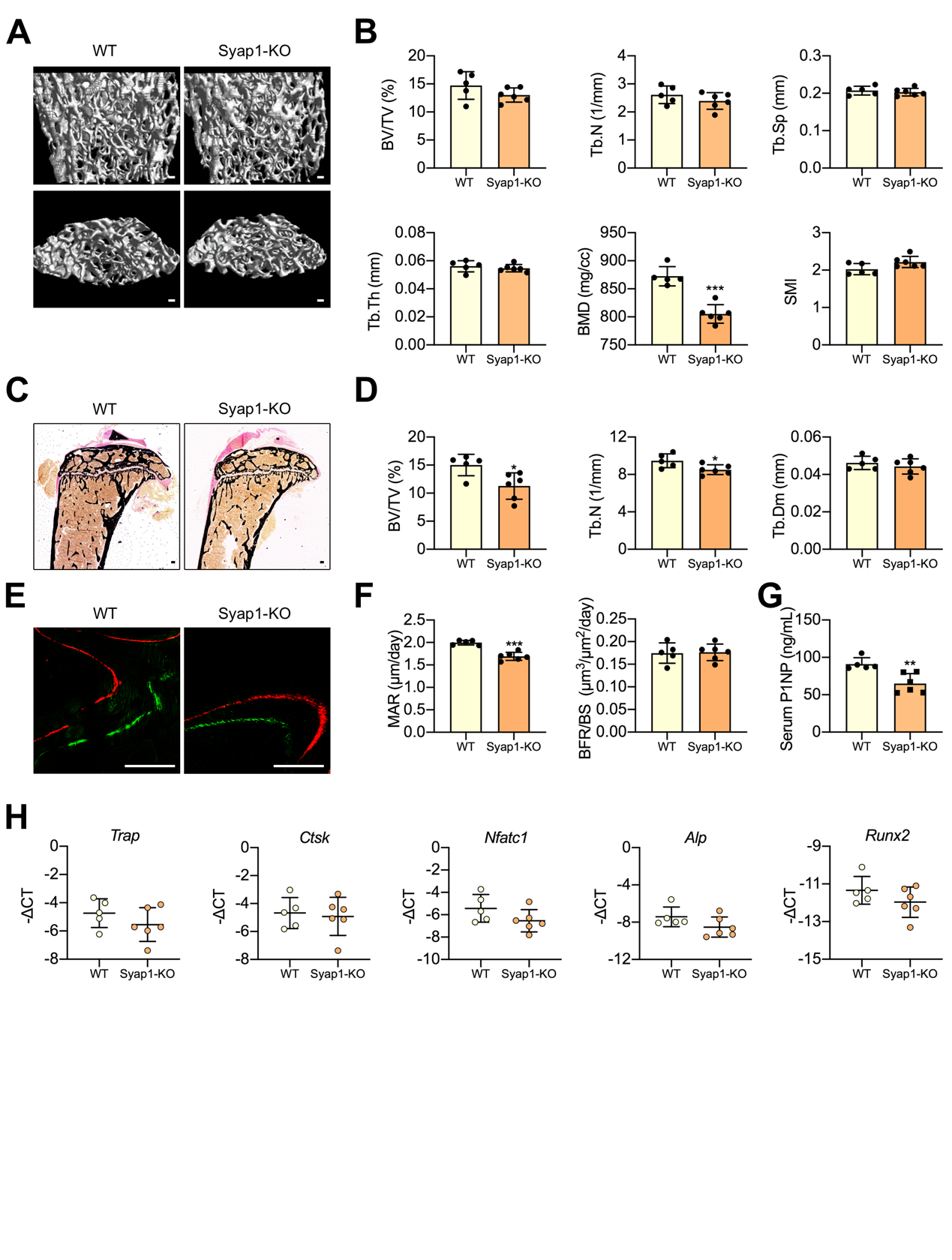
**

**Fig S10. The bone mass phenotype of Syap1-KO mice.**

(A) Representative 3D micro-CT reconstruction images of distal femurs from 12-week-old WT and Syap1-KO male mice.

(B) Quantitative micro-CT analysis of the images shown in (A).

(C) Von Kossa staining of undecalcified tibial sections from 12-week-old WT and Syap1-KO male mice.

(D) Bone histomorphometric analysis of the Von Kossa-stained sections shown in (C).

(E) Double label staining with calcein and alizarin red of undecalcified tibial sections from 12-week-old WT and Syap1-KO male mice.

(F) Bone histomorphometric analysis of double label stained sections shown in (G).

(G) Serum levels of P1NP.

(H) Quantitative real-time PCR analysis was performed to detect the expression of *Trap*, *Ctsk*, *Nfatc1*, *Alp* and *Runx2* in bone tissues of 12-week-old WT and Syap1-KO male mice. The results are presented as -△CT and normalized to the expression of the housekeeping gene GAPDH.

(Scale bar, 100 μm, mean±SD, Student's tests, * *p* < 0.05, ** *p* < 0.01, *** *p* <0.001, WT: n=5, Syap1-KO: n=6)

**
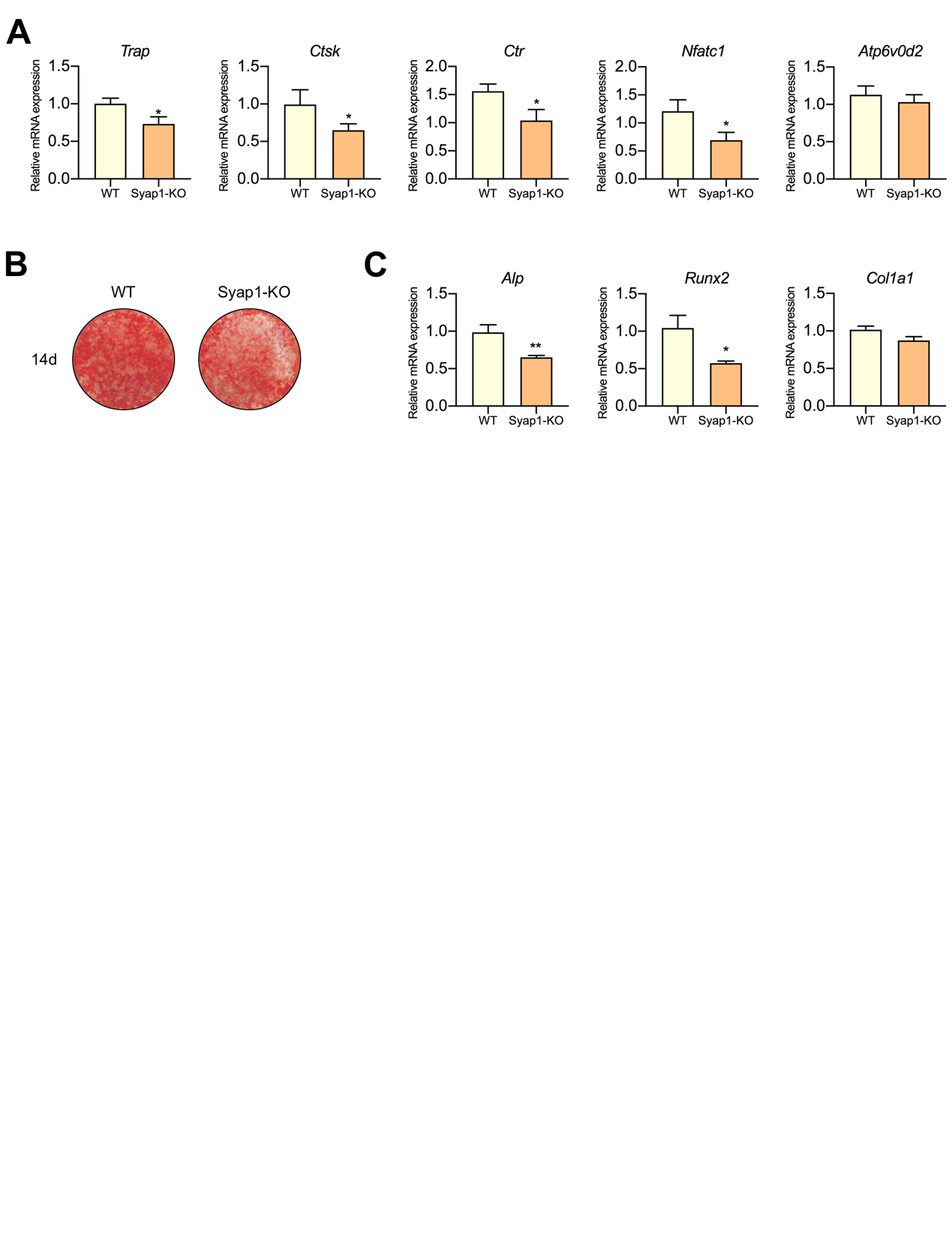
**

**Fig S11. Knockout of Syap1 inhibits both osteoclastogenesis and osteogenesis *in vitro.***

(A) Quantitative real-time PCR analysis was performed to detect the expression of *Trap*, *Ctsk*, *Ctr*, *Nfatc1* and *Atp6v0d2* in WT and Syap1-KO osteoclasts.

(B) Representative images of alizarin red staining. BMSCs obtained from WT and Syap1-KO male mice were stimulated with 10 mM β-glycerophosphate, 10^-7^ mM dexamethasone and 50 μg/ml ascorbic acid for 14 days and stained with alizarin red solution on day 14.

(C) Quantitative real-time PCR analysis was performed to detect the expression of *Alp*, *Runx2* and *Col1a1* in WT and Syap1-KO osteoblasts in (B). The results are normalized to the expression of the housekeeping gene GAPDH.

(Mean±SD, Student's tests, * *p* < 0.05, ** *p* < 0.01, n=3 independent samples)

**
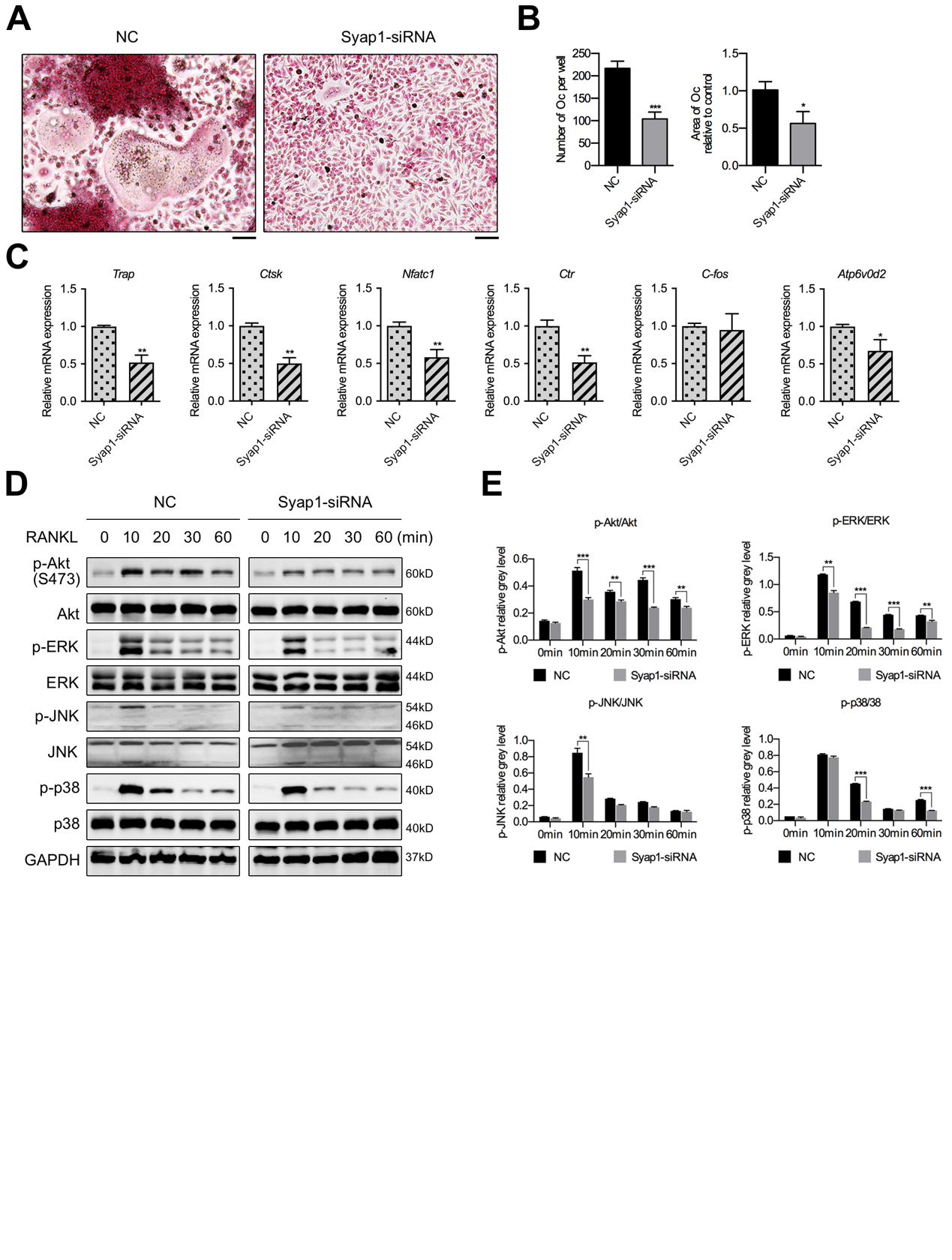
**

**Fig S12. Silencing of Syap1 inhibits RANKL-induced osteoclastogenesis by suppressing PI3K/Akt signaling pathway.**

(A) Representative images of TRAP-stained multinucleated osteoclasts. BMMs were transfected with negative control (NC-siRNA) and Syap1-siRNA for 48 hrs and stimulated with 30 ng/ml M-CSF and 50 ng/ml RANKL for 5 days.

(B) The number and size of the TRAP^+^ multinucleated osteoclasts in (A) were quantified.

(C) Quantitative real-time PCR analysis was performed to detect the expression of *Trap*, *Ctsk*, *Nfatc1*, *Ctr*, *c-fos* and *Atp6v0d2* in NC and Syap1-siRNA groups. The results were normalized to the expression of the housekeeping gene GAPDH.

(D) Western blot analysis was performed to detect total and phosphorylated forms of Akt, ERK, JNK and p38. BMMs were transfected with NC-siRNA and Syap1-siRNA for 48 hrs followed by stimulation with 50 ng/ml RANKL for 10, 20, 30, and 60 mins.

(E) Quantitative densitometric analysis of Akt, ERK, JNK and p38 phosphorylation normalized to total protein expression.

(Scale bar, 200 μm, mean±SD, Student's tests, * *p* < 0.05, ** *p* < 0.01, *** *p* <0.001, n=3 independent samples)

**
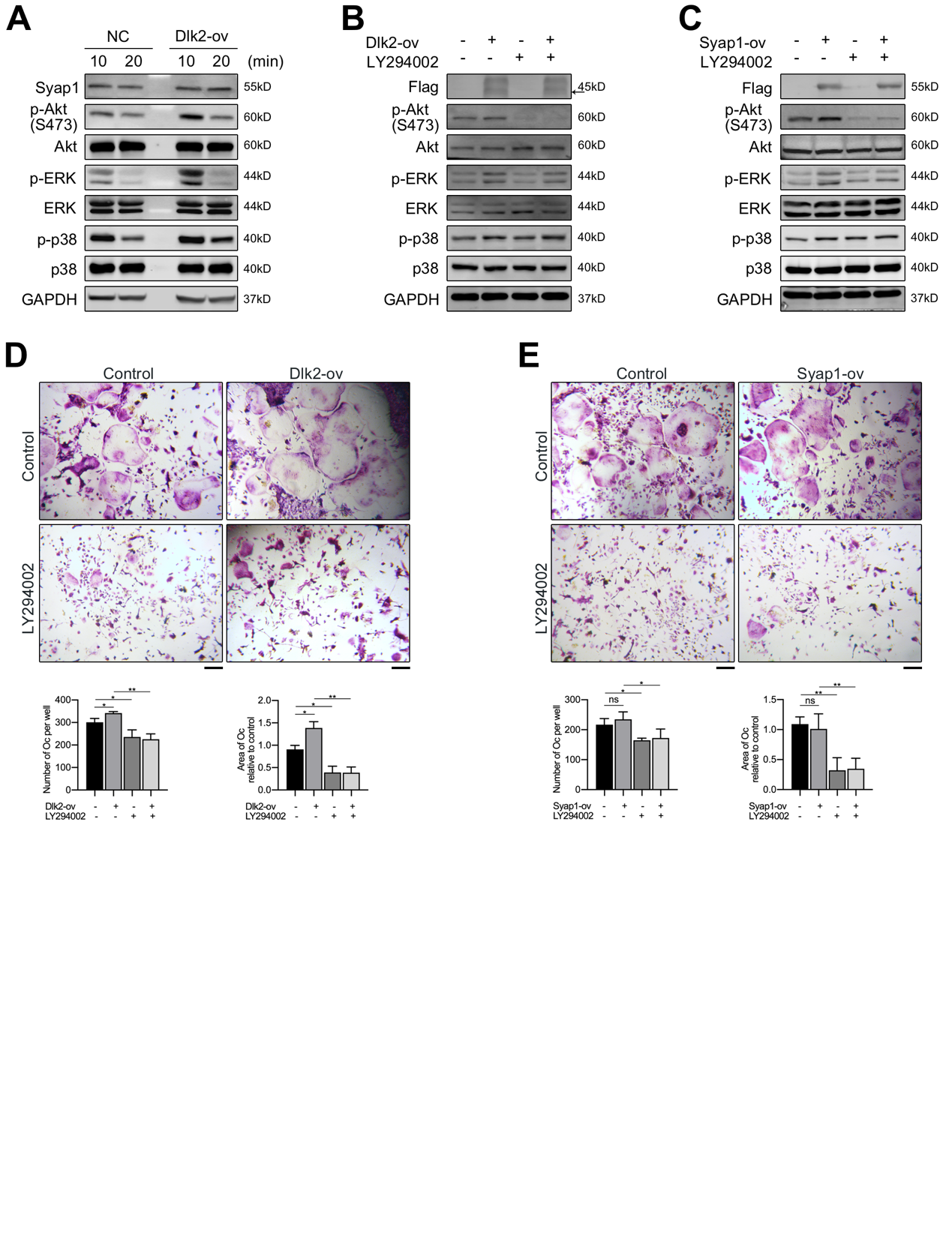
**

**Fig S13. The role of the Dlk2-Syap1-Akt axis in osteoclastogenesis.**

(A) Western blot analysis was performed to detect Syap1 and total and phosphorylated forms of Akt, ERK and p38. BMMs were transfected with NC lentivirus and Dlk2-overexpressing lentivirus for 48 hrs followed by stimulation with 50 ng/ml RANKL for 10 and 20 mins.

(B) Western blot analysis was performed to detect Flag and total and phosphorylated forms of Akt, ERK and p38. BMMs were transfected with NC lentivirus or Dlk2-overexpressing lentivirus for 48 hrs. Then, BMMs were treated with or without 10 μM LY294002 for 2 hrs, followed by stimulation with 50 ng/ml RANKL for 10 mins.

(C) Western blot analysis was performed to detect Flag and total and phosphorylated forms of Akt, ERK and p38. BMMs were transfected with NC lentivirus or Syap1-overexpressing lentivirus for 48 hrs. Then, BMMs were treated with or without 10 μM LY294002 for 2 hrs, followed by stimulation with 50 ng/ml RANKL for 10 mins.

(D) Representative images of TRAP-stained multinucleated osteoclasts. BMMs were transfected with NC lentivirus or Dlk2-overexpressing lentivirus for 48 hrs and then stimulated with 30 ng/ml M-CSF and 50 ng/ml RANKL with or without 10 μM LY294002 for 5 days. The number and size of the TRAP^+^ multinucleated osteoclasts were quantified.

(E) Representative images of TRAP-stained multinucleated osteoclasts. BMMs were transfected with NC lentivirus or Syap1-overexpressing lentivirus for 48 hrs and then stimulated with 30 ng/ml M-CSF and 50 ng/ml RANKL with or without 10 μM LY294002 for 5 days. The number and size of the TRAP^+^ multinucleated osteoclasts were quantified.

(Scale bar, 200 μm, mean±SD, Student's tests, * *p* < 0.05, ** *p* < 0.01, n=3 independent samples)

**
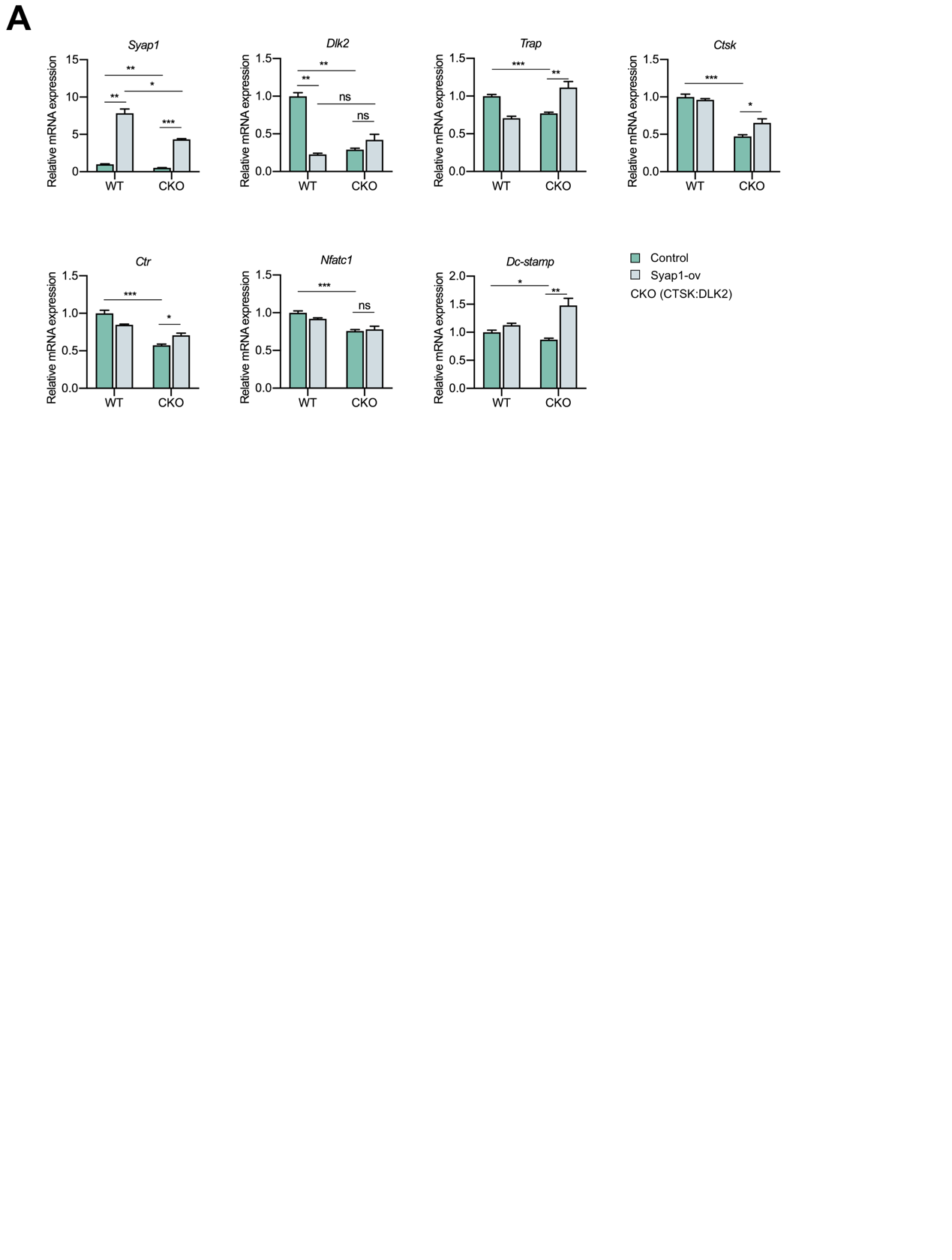
**

**Fig S14.** **Overexpression of Syap1 in Dlk2-deficient osteoclasts rescued the expression level of osteoclastic marker genes.**

(A) Quantitative real-time PCR analysis was performed to detect the expression of *Syap1*, *Dlk2*, *Trap*, *Ctsk*, *Ctr*, *Nfatc1* and *Dc-stamp* in WT, WT-Syap1-ov, CKO (Ctsk-Cre^+^;Dlk2^fl/fl^) and CKO-Syap1-ov groups. The results were normalized to the expression of the housekeeping gene GAPDH.

(Mean±SD, Student's tests, * *p* < 0.05, ** *p* < 0.01, *** *p* <0.001, n=3 independent samples. NS, not significant)

**
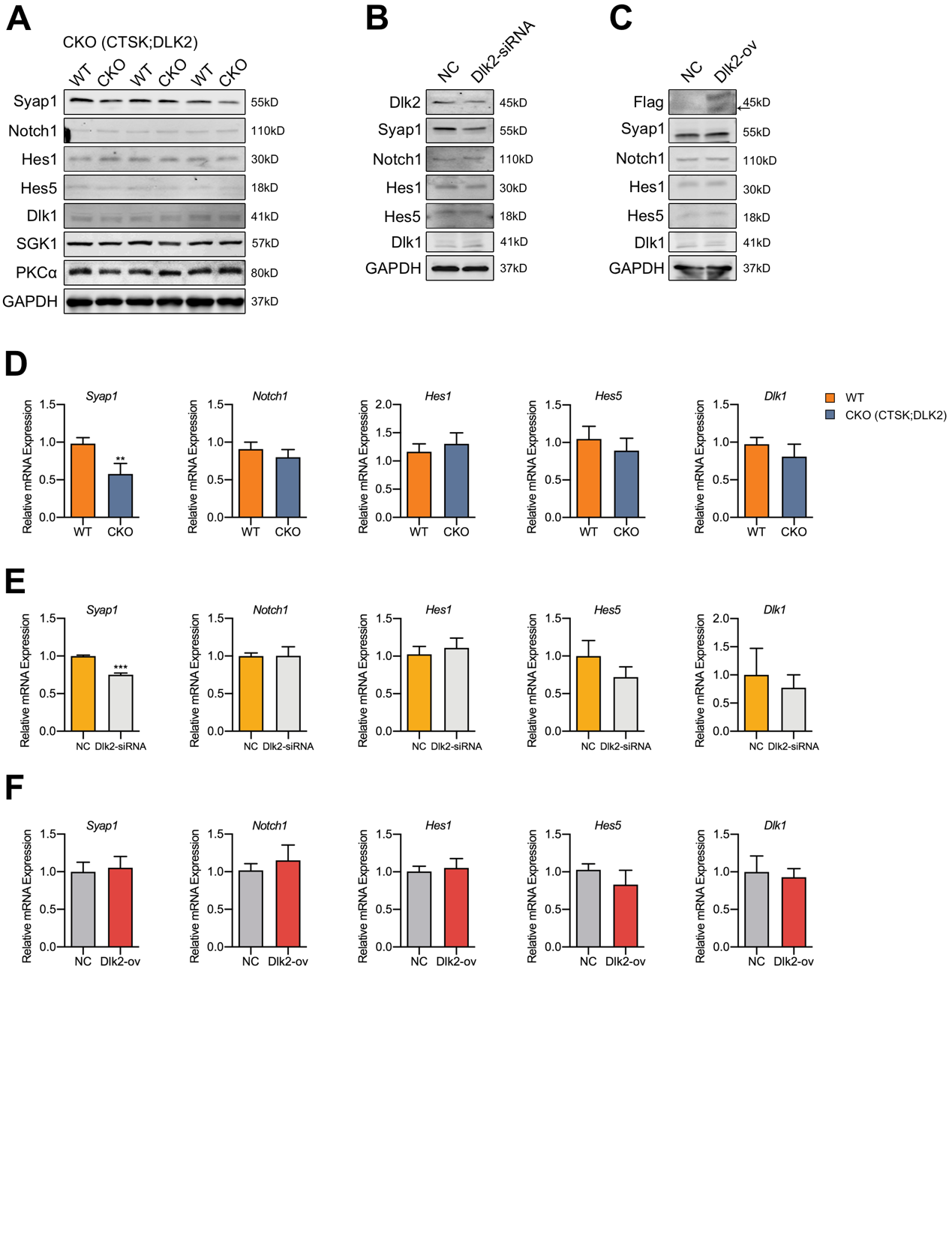
**

**Fig S15. The involvement of Notch1 signaling and Dlk1 expression in Dlk2-deficient osteoclasts and Dlk2-ov osteoclasts.**

(A) Western blot analysis was performed to detect Syap1, Notch1, Hes1 and Hes5 in Ctsk-Cre^-^;Dlk2^fl/fl^ (WT) and Ctsk-Cre^+^;Dlk2^fl/fl^ (CKO) osteoclasts.

(B) Western blot analysis was performed to detect Dlk2, Syap1, Notch1, Hes1, Hes5 and Dlk1 in NC and Dlk2-siRNA osteoclasts.

(C) Western blot analysis was performed to detect Flag, Syap1, Notch1, Hes1, Hes5 and Dlk1 in NC and Dlk2-ov osteoclasts.

(D) Quantitative real-time PCR analysis was performed to detect the expression of *Syap1*, *Notch1*, *Hes1*, *Hes5* and *Dlk1* in WT and CKO groups.

(E) Quantitative real-time PCR analysis was performed to detect the expression of *Syap1*, *Notch1*, *Hes1*, *Hes5* and *Dlk1* in NC and Dlk2-siRNA groups.

(F) Quantitative real-time PCR analysis was performed to detect the expression of *Syap1*, *Notch1*, *Hes1*, *Hes5* and *Dlk1* in NC and Dlk2-ov groups. The results were normalized to the expression of the housekeeping gene GAPDH.

(Mean±SD, Student's tests, * *p* < 0.05, n=3 independent samples)
