# Supplementary material for: Dlk2 interacts with Syap1 to activate Akt signaling pathway during osteoclast formation
Source: Cell Death Dis. 2023 Sep 5;14(9):589. doi: 10.1038/s41419-023-06107-1 (PMC10480461; doi:10.1038/s41419-023-06107-1)
Supplement: Supplementary file 2 — Original western blots [file 41419_2023_6107_MOESM2_ESM.pdf]

Supplemental Material

Original western blots

Fig 4D

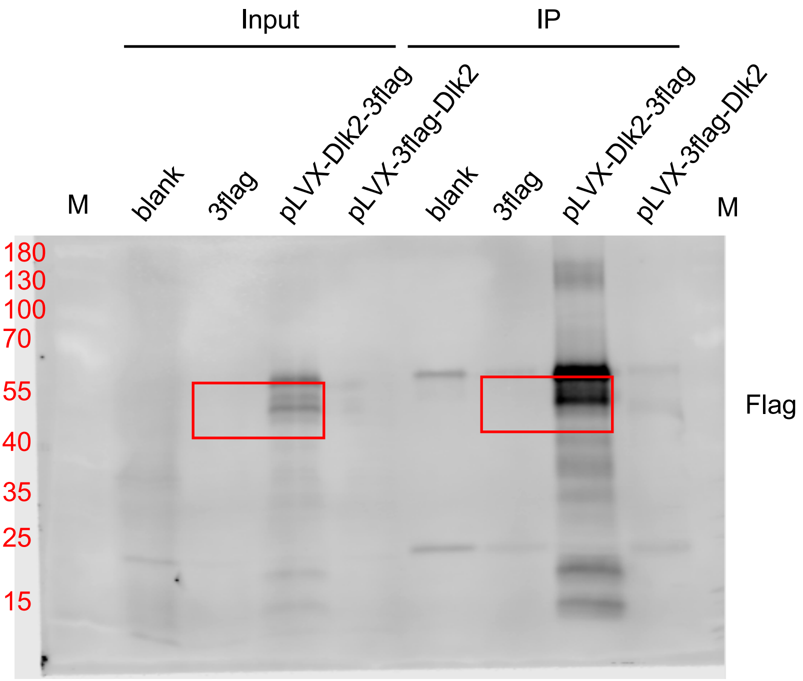
Input
IP
M
blank
3flag
pLVX-Dlk2-3flag
pLVX-3flag-Dlk2
blank
3flag
pLVX-Dlk2-3flag
pLVX-3flag-Dlk2
M
180
130
100
70
55
40
35
25
15
Flag

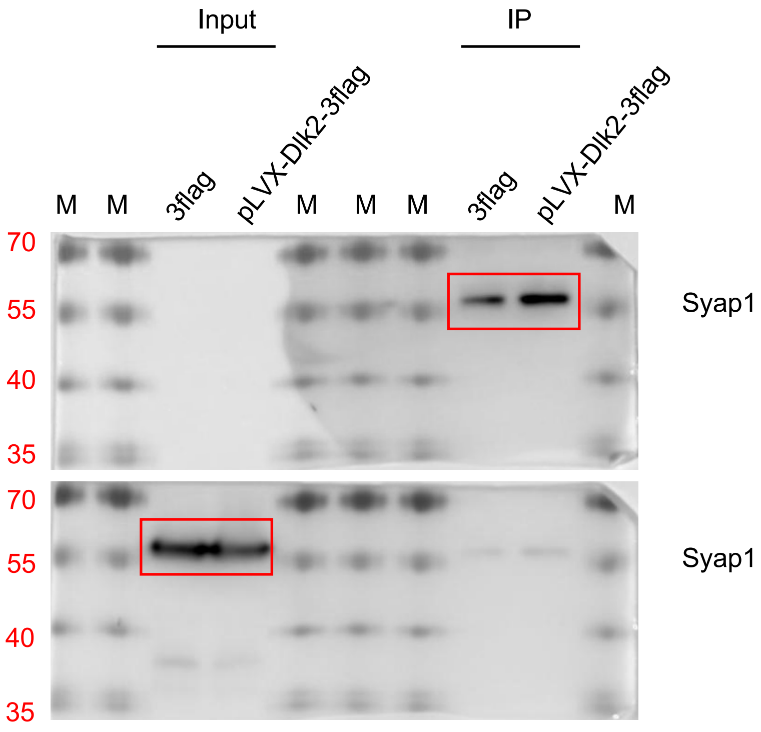
Input
IP
M
M
3flag
pLVX-Dlk2-3flag
M
M
M
3flag
pLVX-Dlk2-3flag
M
70
55
40
35
Syap1
70
55
40
35
Syap1

Fig 4E

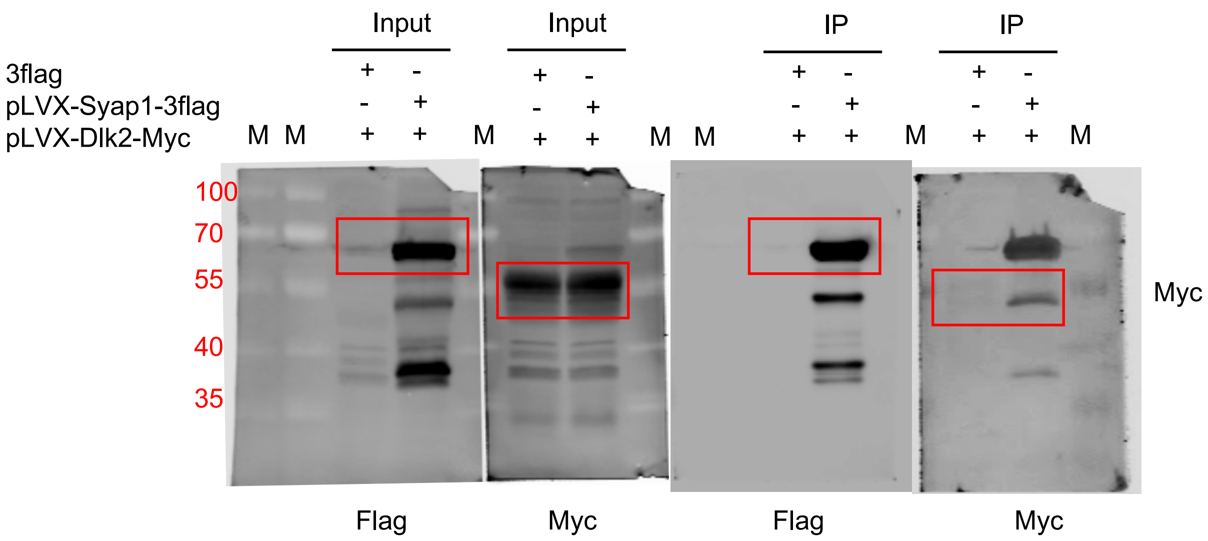

Input
Input
IP
IP
3flag
pLVX-Syap1-3flag
pLVX-Dlk2-Myc
M M + + M + + M M + + M + + M
100
70
55
40
35
Myc
Flag
Myc
Flag
Myc

Fig 4F

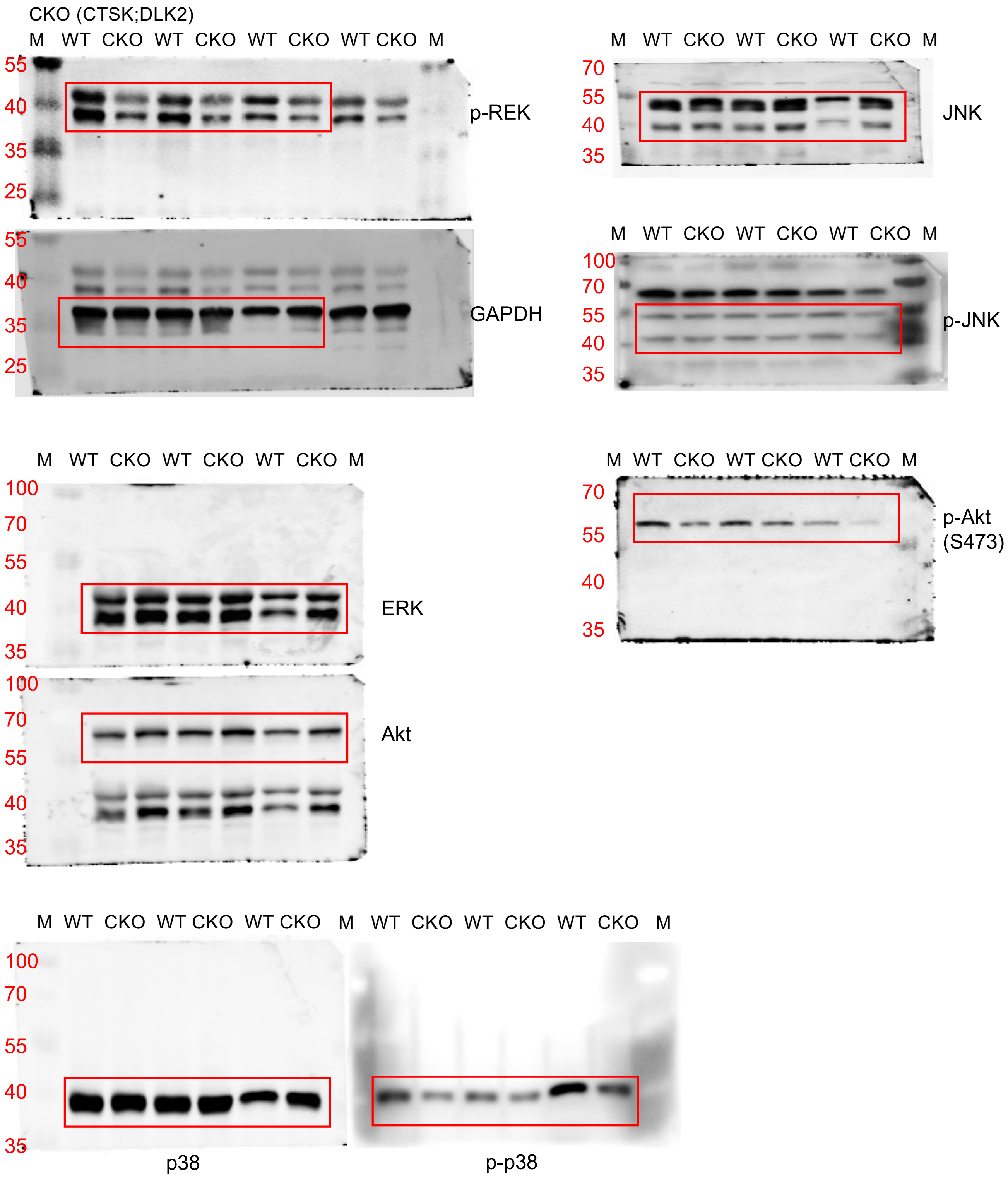

CKO (CTSK;DLK2)
M WT CKO WT CKO WT CKO WT CKO M
55
40
35
25
p-REK
55
40
35
25
GAPDH
M WT CKO WT CKO WT CKO M
70
55
40
35
JNK
M WT CKO WT CKO WT CKO M
100
70
55
40
35
p-JNK
M WT CKO WT CKO WT CKO M
100
70
55
40
35
ERK
100
70
55
40
35
Akt
M WT CKO WT CKO WT CKO M
70
55
40
35
p-Akt
(S473)
M WT CKO WT CKO WT CKO M WT CKO WT CKO WT CKO M
100
70
55
40
35
p38
p-p38

Fig 5E

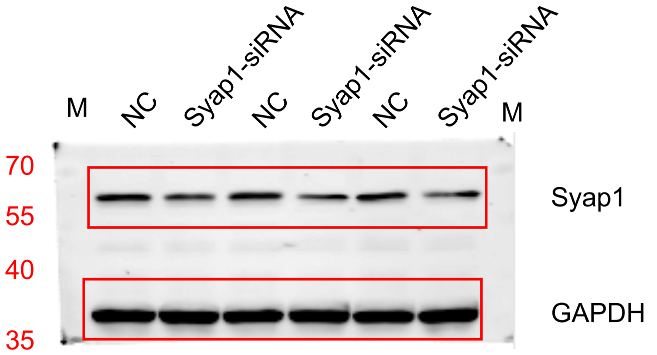
M
NC
Syap1-siRNA
NC
Syap1-siRNA
NC
Syap1-siRNA
M
70
55
40
35
Syap1
GAPDH

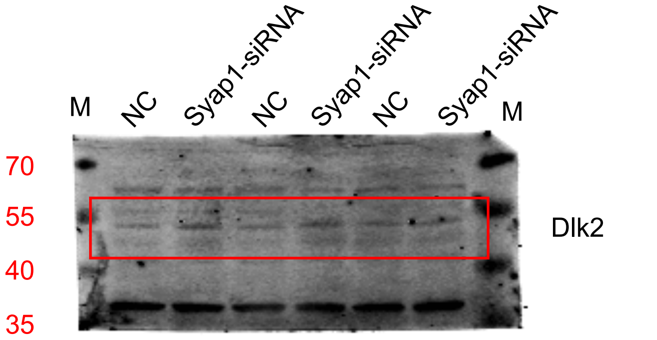
M
NC
Syap1-siRNA
NC
Syap1-siRNA
NC
Syap1-siRNA
M
70
55
40
35
Dlk2

Fig 5F

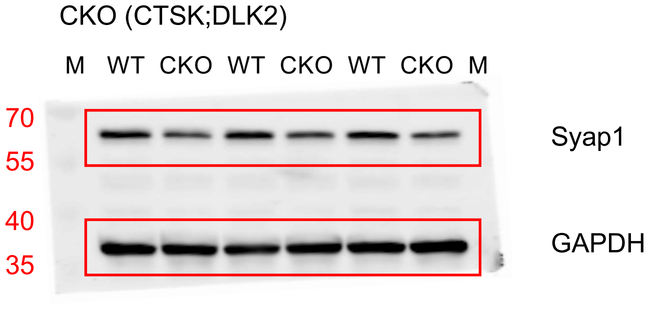
CKO (CTSK;DLK2)
M WT CKO WT CKO WT CKO M
70
55
40
35
Syap1
GAPDH

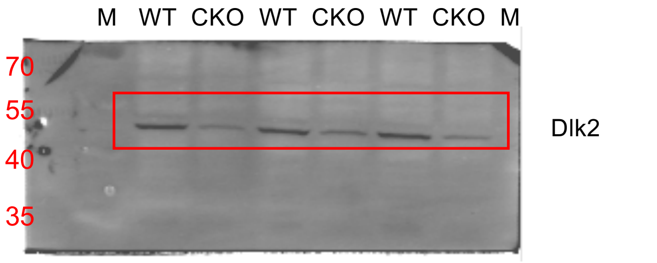
M WT CKO WT CKO WT CKO M
70
55
40
35
Dlk2

Fig 5G

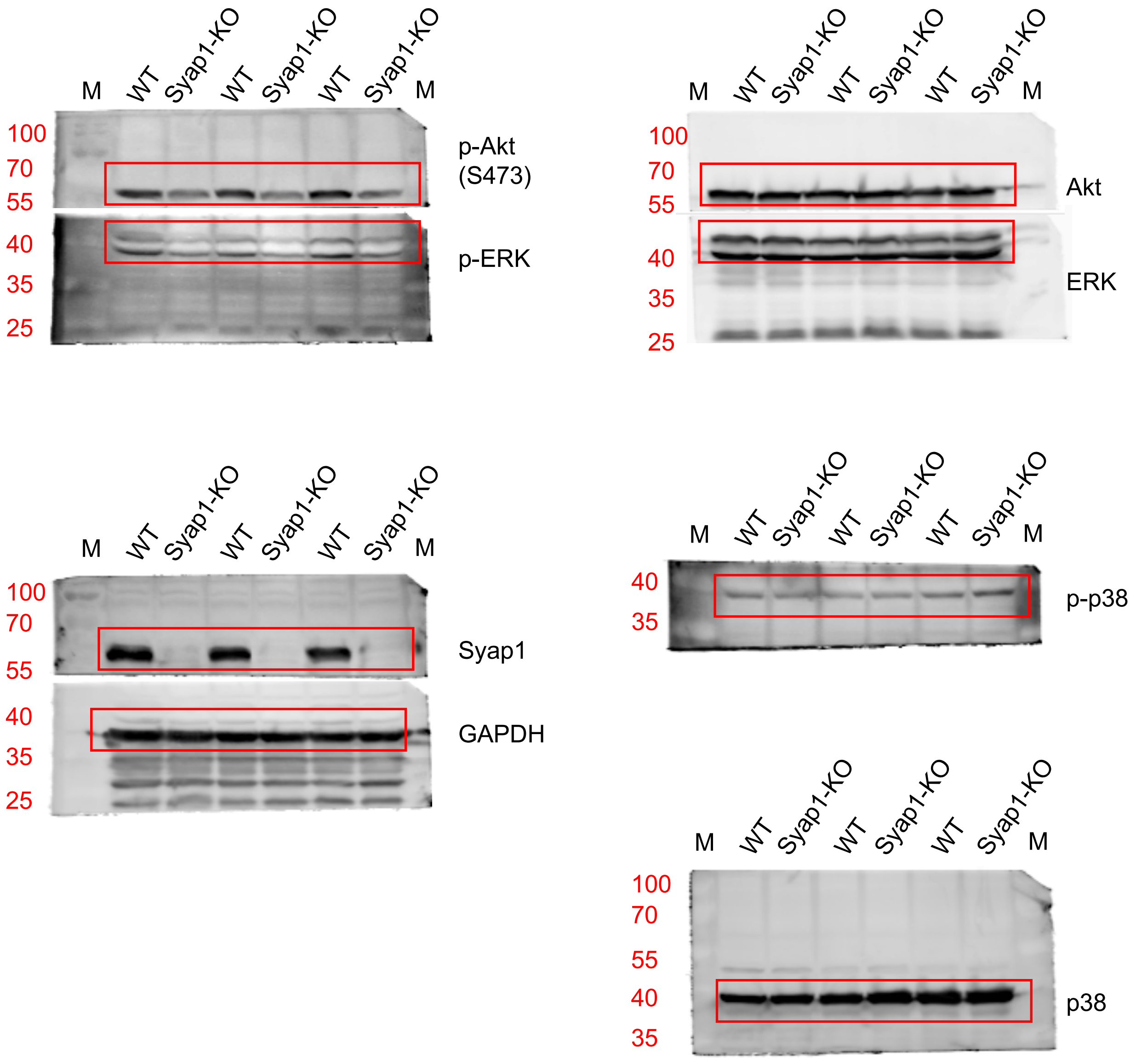
M
WT
Syap1-KO
WT
Syap1-KO
WT
Syap1-KO
M
100
70
55
40
35
25
p-Akt (S473)
p-ERK
Akt
ERK
Syap1
GAPDH
p-p38
p38

Fig 5I

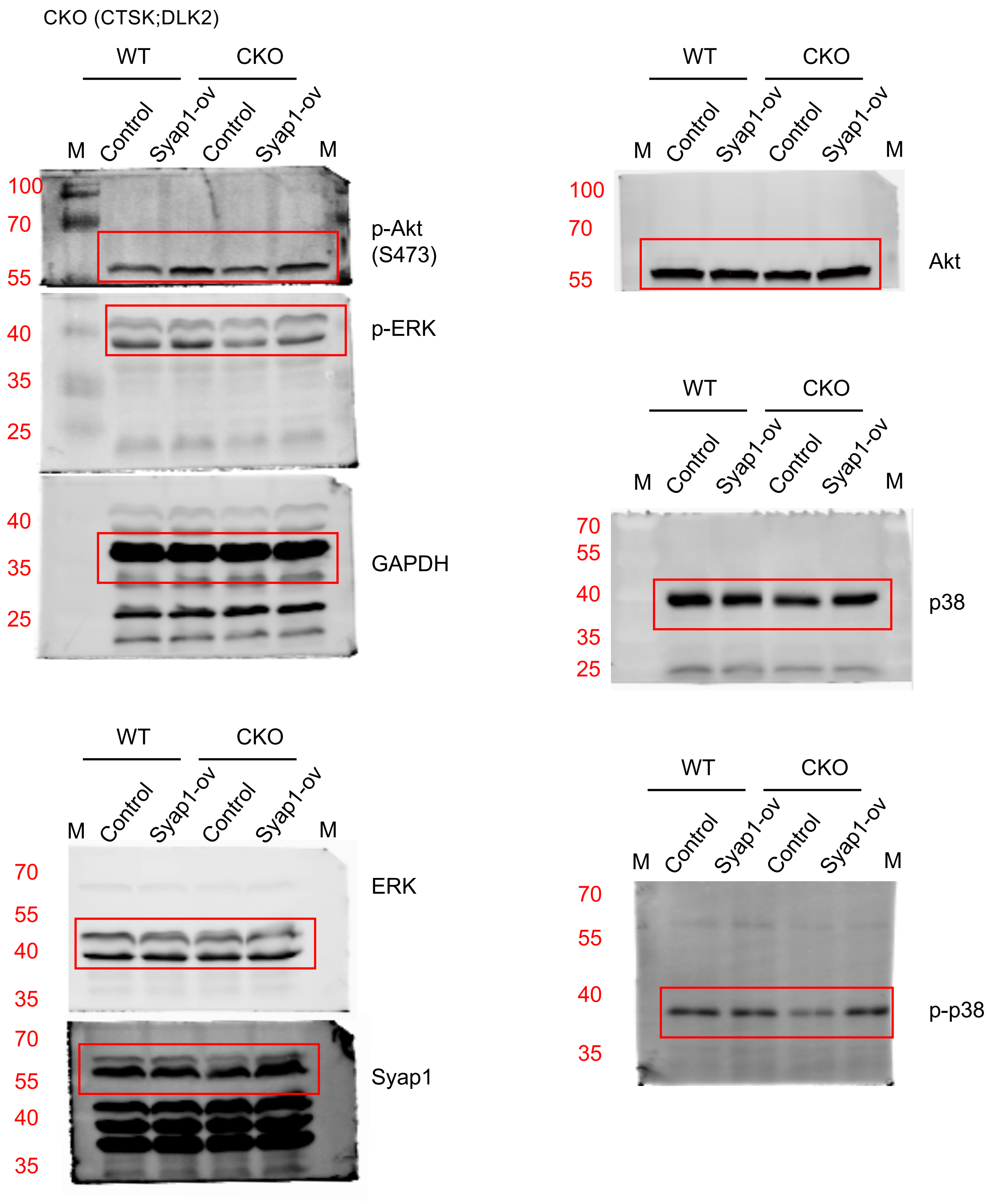
CKO (CTSK;DLK2)
WT
CKO
M
Control
Syap1-ov
Control
Syap1-ov
M
100
70
55
p-Akt (S473)
40
35
25
p-ERK
40
35
25
GAPDH
Akt
p38
70
55
40
35
ERK
70
55
40
35
Syap1
p-p38

Fig 5K

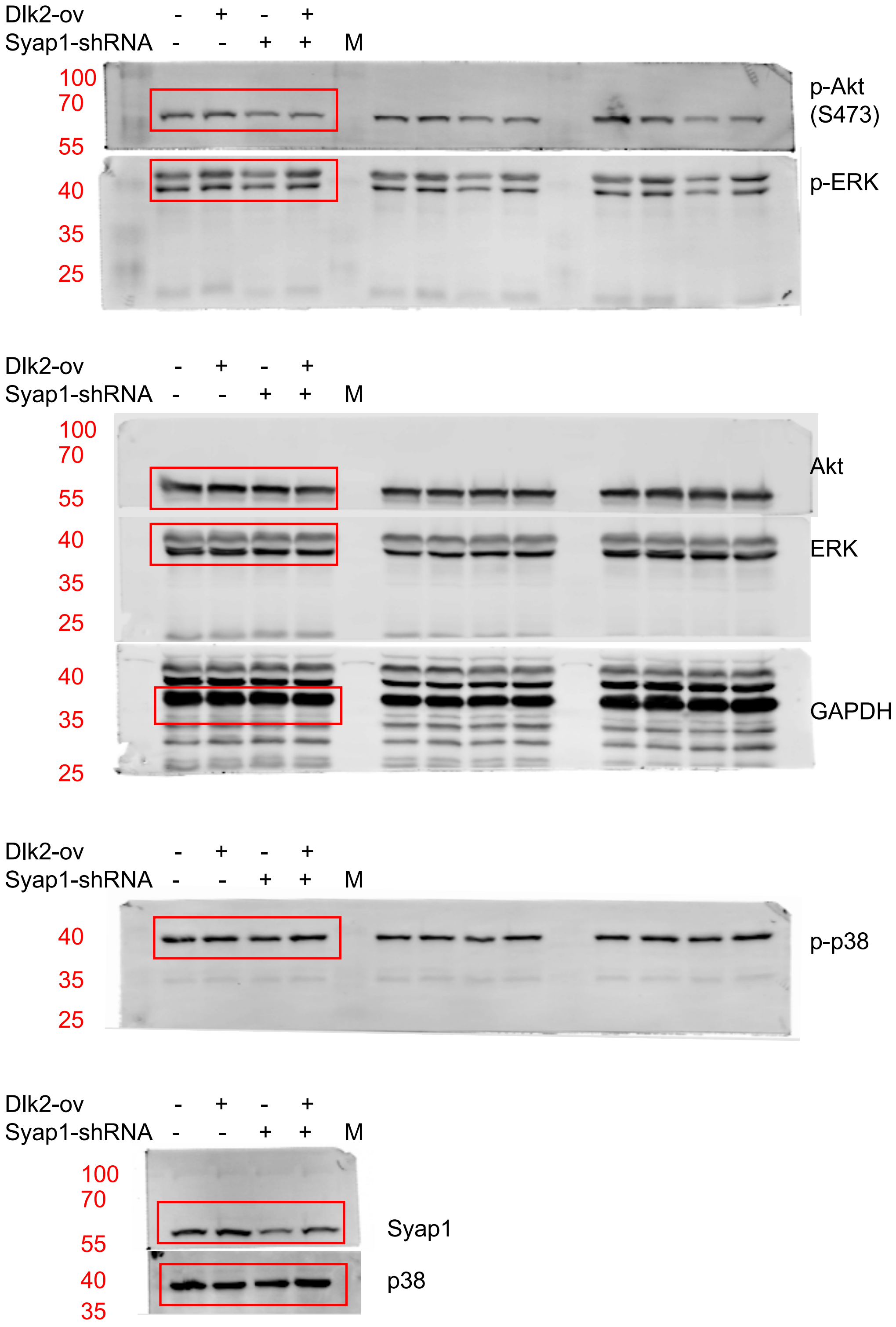
Dlk2-ov - + - +
Syap1-shRNA - - + + M
100
70
55
40
35
25
p-Akt (S473)
p-ERK
Dlk2-ov - + - +
Syap1-shRNA - - + + M
100
70
55
40
35
25
40
35
25
Akt
ERK
GAPDH
Dlk2-ov - + - +
Syap1-shRNA - - + + M
40
35
25
p-p38
Dlk2-ov - + - +
Syap1-shRNA - - + + M
100
70
55
40
35
Syap1
p38

Fig S1B

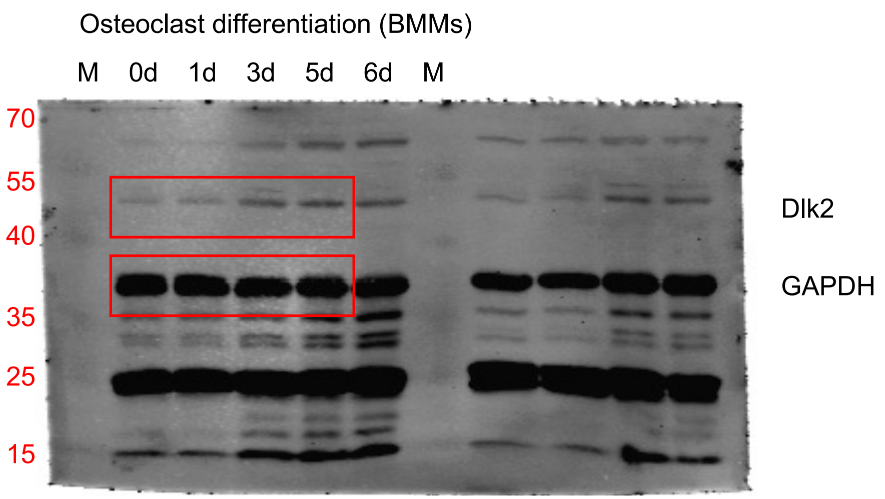

Fig S1C

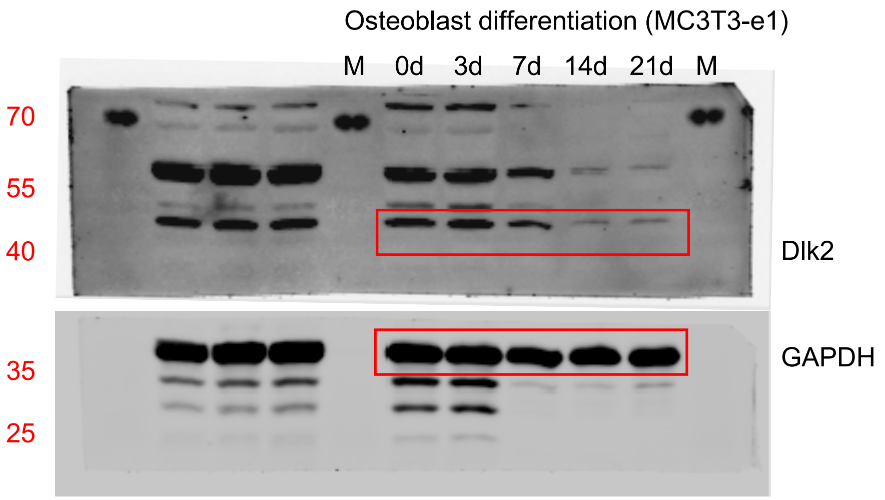

Fig S1E

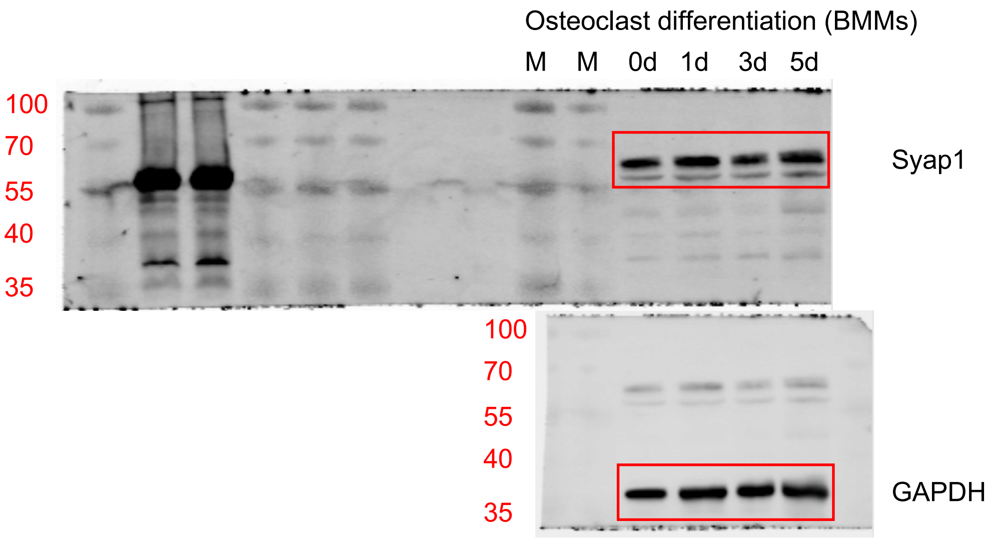

Fig S4E

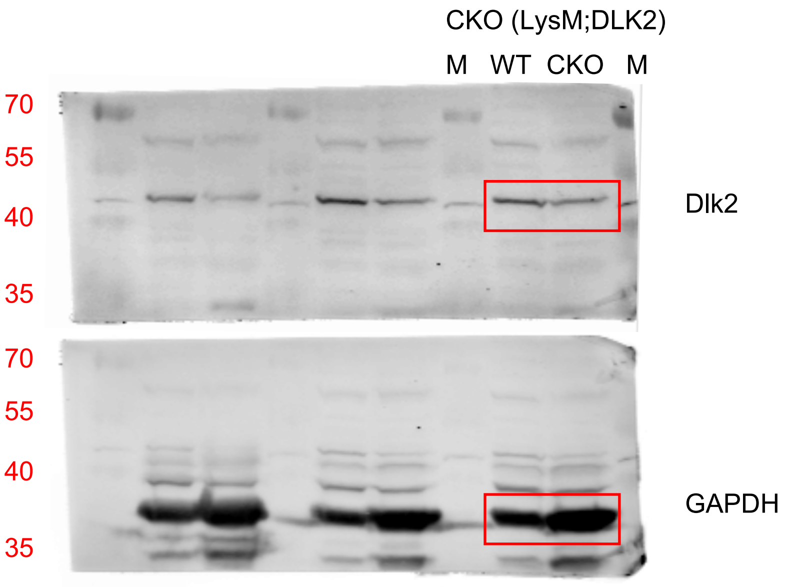

Fig S6E

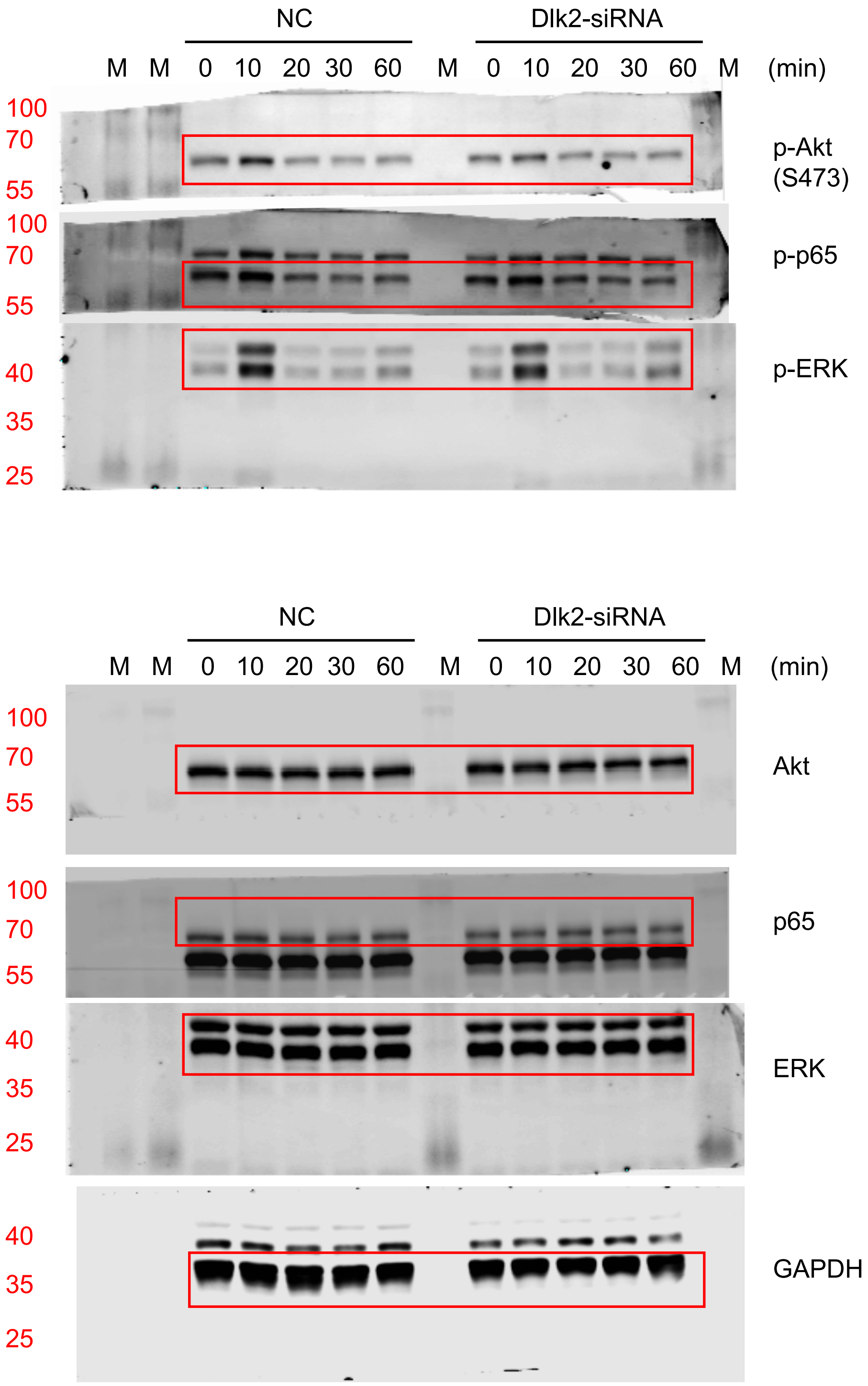
NC
Dlk2-siRNA
M M 0 10 20 30 60 M 0 10 20 30 60 M (min)
100
70
55
100
70
55
40
35
25
p-Akt (S473)
p-p65
p-ERK
NC
Dlk2-siRNA
M M 0 10 20 30 60 M 0 10 20 30 60 M (min)
100
70
55
100
70
55
40
35
25
40
35
25
Akt
p65
ERK
GAPDH

Fig S6E

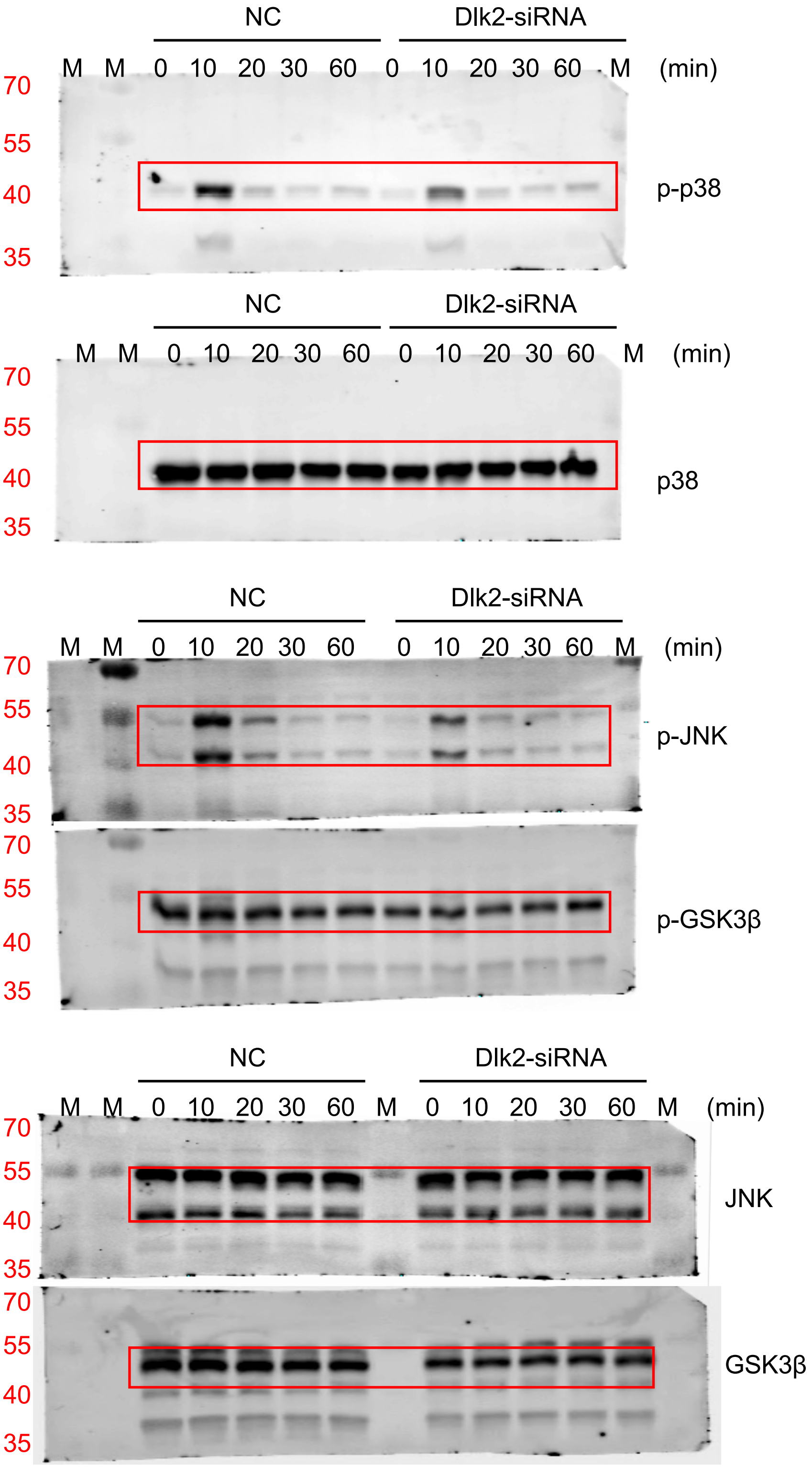
NC
Dlk2-siRNA
M M 0 10 20 30 60 0 10 20 30 60 M (min)
70
55
40
35
p-p38
NC
Dlk2-siRNA
M M 0 10 20 30 60 0 10 20 30 60 M (min)
70
55
40
35
p38
NC
Dlk2-siRNA
M M 0 10 20 30 60 0 10 20 30 60 M (min)
70
55
40
35
p-JNK
70
55
40
35
p-GSK3β
NC
Dlk2-siRNA
M M 0 10 20 30 60 M 0 10 20 30 60 M (min)
70
55
40
35
JNK
70
55
40
35
GSK3β

Fig S9D

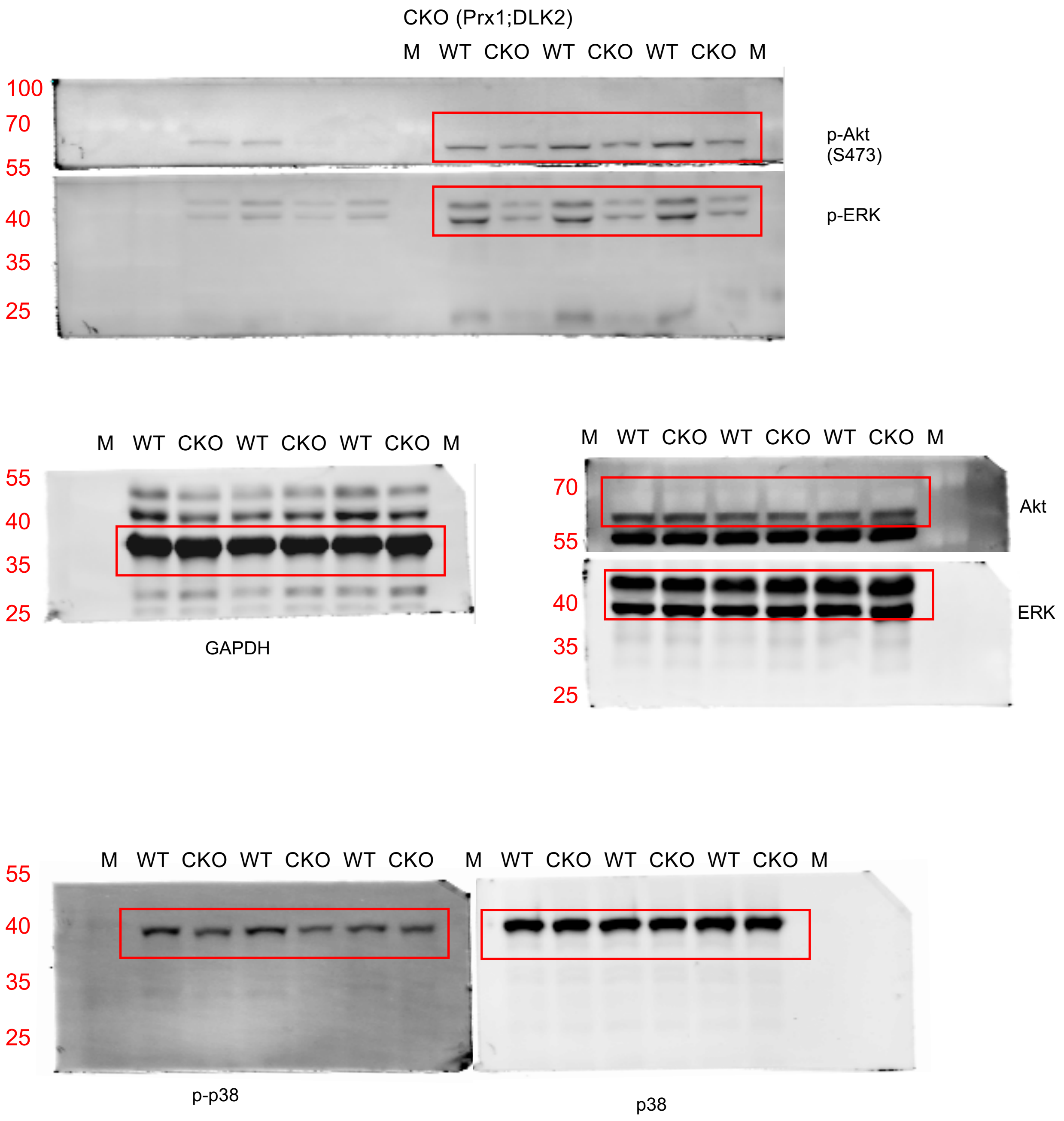
CKO (Prx1;DLK2)
M WT CKO WT CKO WT CKO M
100
70
55
40
35
25
p-Akt (S473)
p-ERK
M WT CKO WT CKO WT CKO M
55
40
35
25
GAPDH
M WT CKO WT CKO WT CKO M
70
55
40
35
25
Akt
ERK
M WT CKO WT CKO WT CKO
M WT CKO WT CKO WT CKO M
55
40
35
25
p-p38
p38

Fig S9E

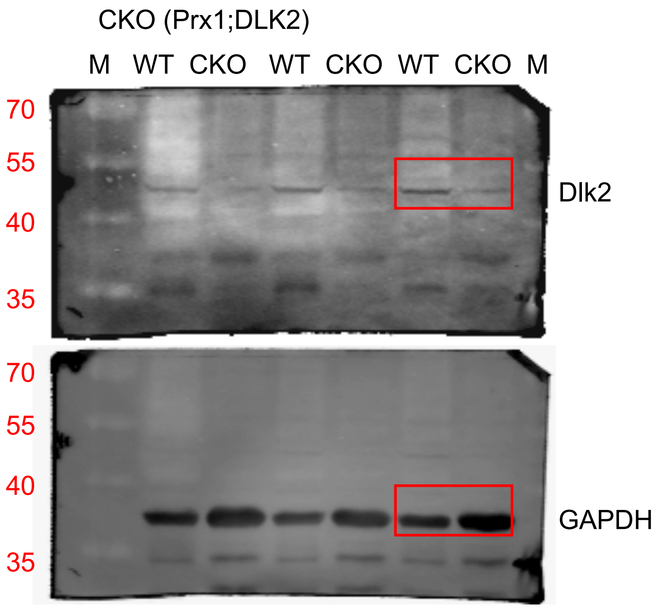

CKO (Prx1;DLK2)
M WT CKO WT CKO WT CKO M
70
55
40
35
Dlk2
70
55
40
35
GAPDH

Fig S12D

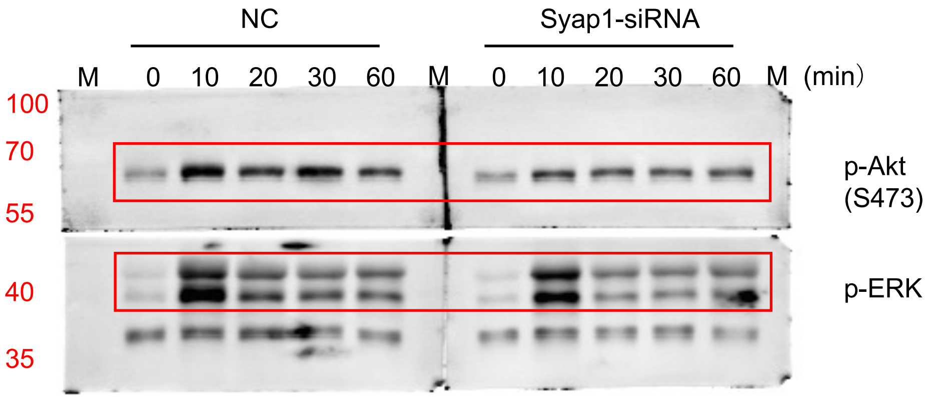
NC
Syap1-siRNA
M 0 10 20 30 60 M 0 10 20 30 60 M (min)
100
70
55
40
35
p-Akt (S473)
p-ERK

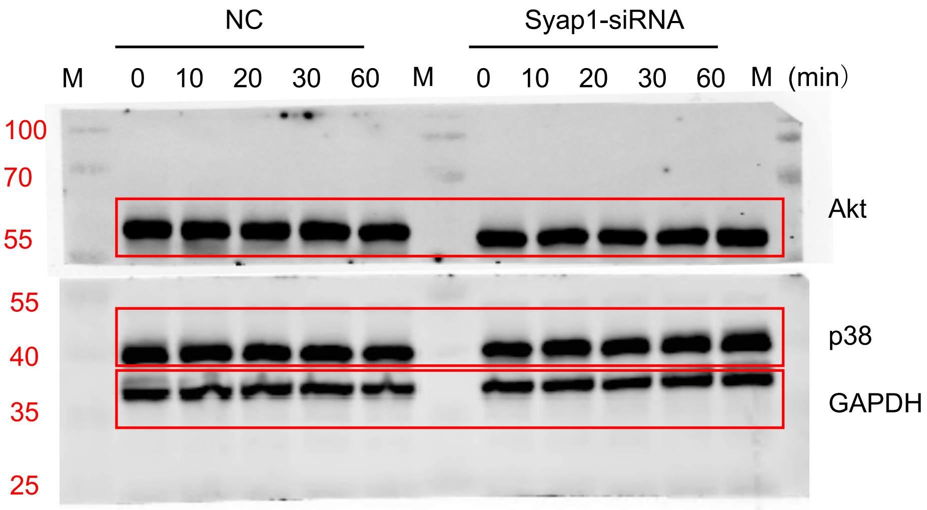
NC
Syap1-siRNA
M 0 10 20 30 60 M 0 10 20 30 60 M (min)
100
70
55
55
40
35
25
Akt
p38
GAPDH

Fig S12D

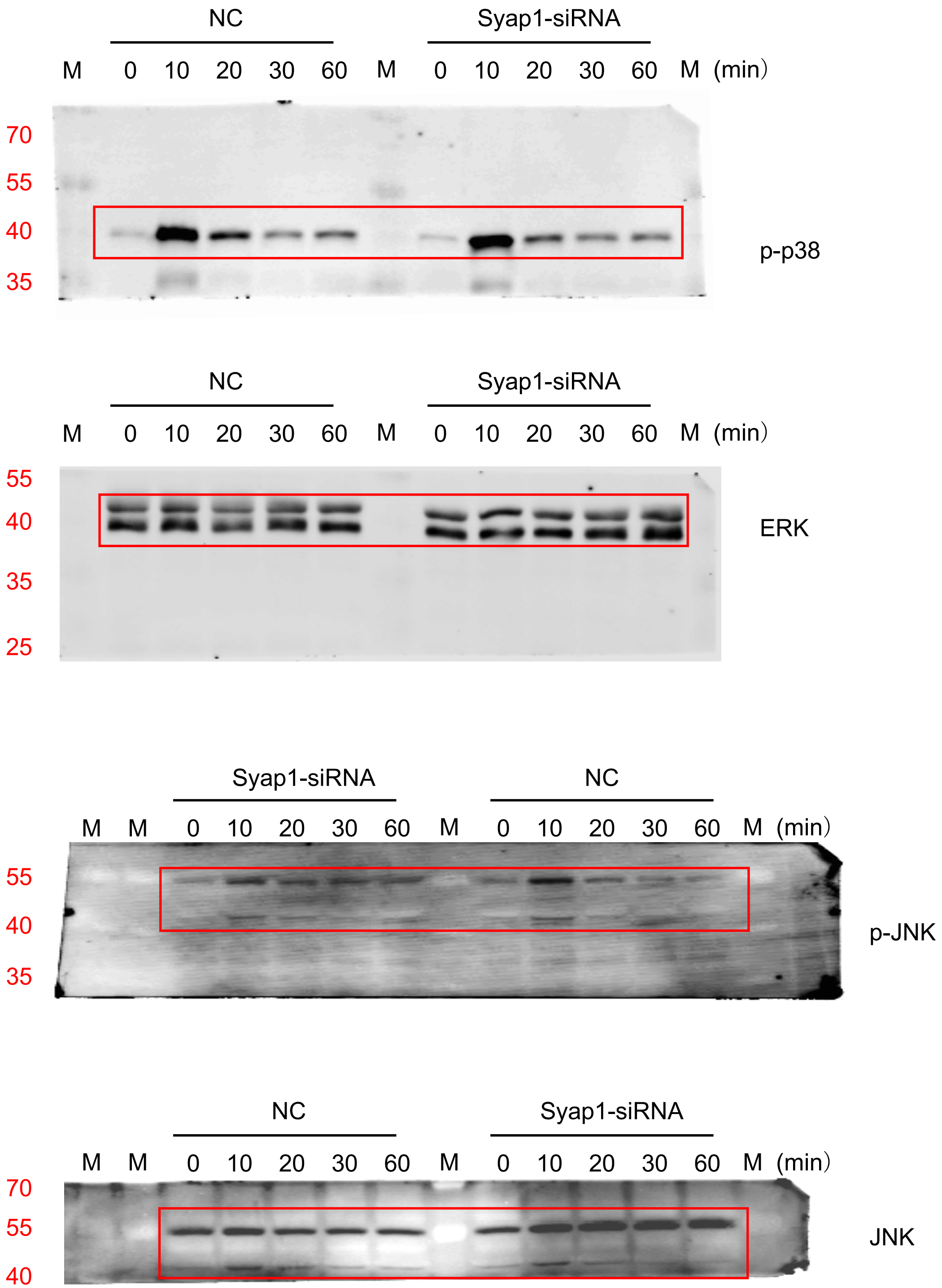
NC
Syap1-siRNA
M 0 10 20 30 60 M 0 10 20 30 60 M (min)
70
55
40
35
p-p38
NC
Syap1-siRNA
M 0 10 20 30 60 M 0 10 20 30 60 M (min)
55
40
35
25
ERK
Syap1-siRNA
NC
M M 0 10 20 30 60 M 0 10 20 30 60 M (min)
55
40
35
p-JNK
NC
Syap1-siRNA
M M 0 10 20 30 60 M 0 10 20 30 60 M (min)
70
55
40
JNK

Fig S13A

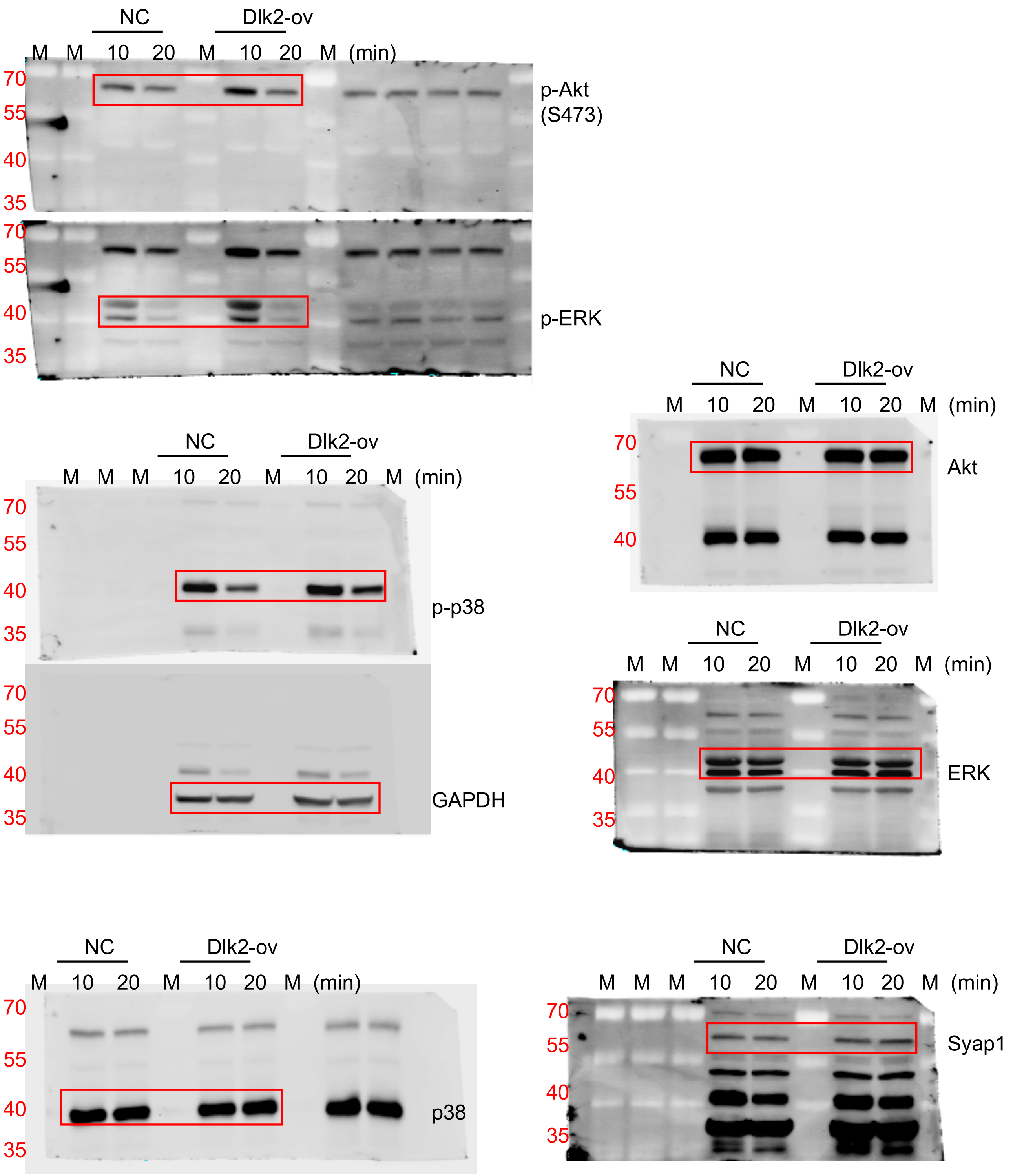

NC
Dlk2-ov
M M 10 20 M 10 20 M (min)
70
55
40
35
p-Akt (S473)
p-ERK
M 10 20 M 10 20 M (min)
Akt
M M M 10 20 M 10 20 M (min)
p-p38
GAPDH
M M 10 20 M 10 20 M (min)
ERK
M 10 20 M 10 20 M (min)
p38
M M M 10 20 M 10 20 M (min)
Syap1

Fig S13B

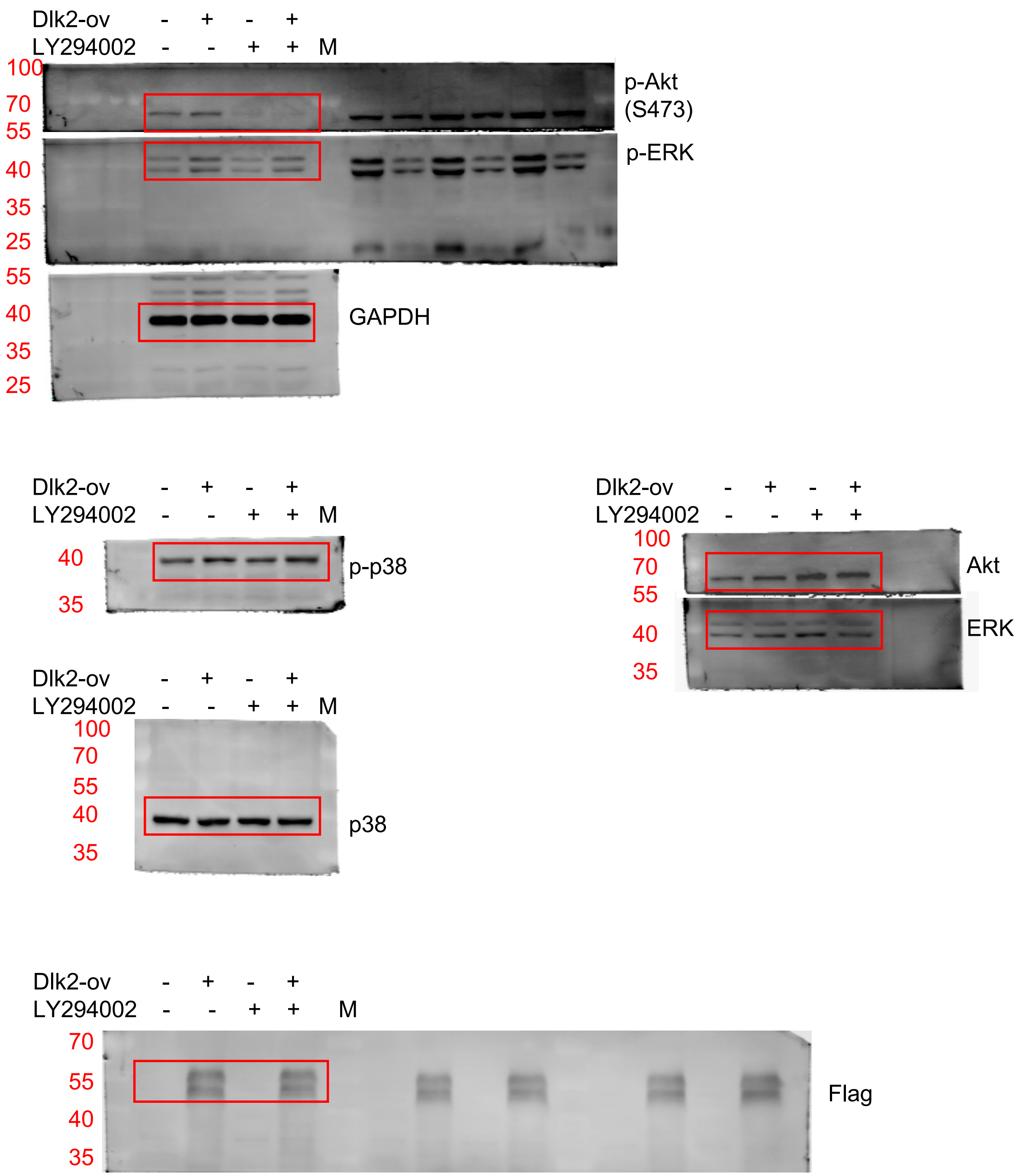
Dlk2-ov - + - +
LY294002 - - + + M
100
70
55
p-Akt (S473)
p-ERK
40
35
25
55
40
35
25
GAPDH
Dlk2-ov - + - +
LY294002 - - + + M
40
35
p-p38
Dlk2-ov - + - +
LY294002 - - + +
100
70
55
Akt
40
35
ERK
Dlk2-ov - + - +
LY294002 - - + + M
100
70
55
40
35
p38
Dlk2-ov - + - +
LY294002 - - + + M
70
55
40
35
Flag

Fig S13C

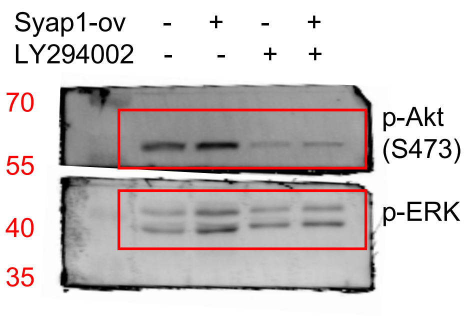

Syap1-ov - + - +
LY294002 - - + +
70
55
40
35
p-Akt (S473)
p-ERK

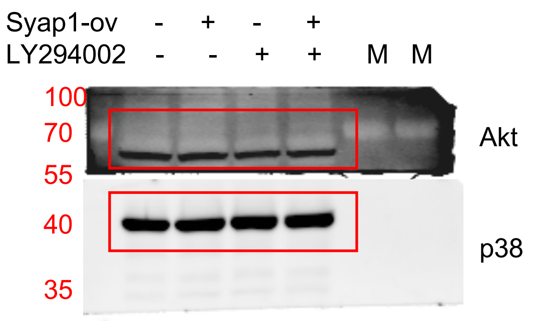

Syap1-ov - + - +
LY294002 - - + + M M
100
70
55
40
35
Akt
p38

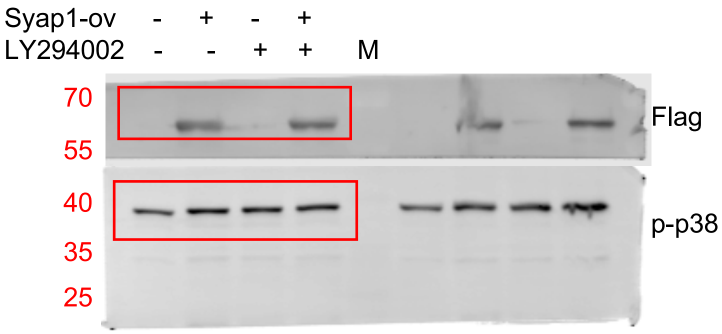

Syap1-ov - + - +
LY294002 - - + + M
70
55
40
35
25
Flag
p-p38

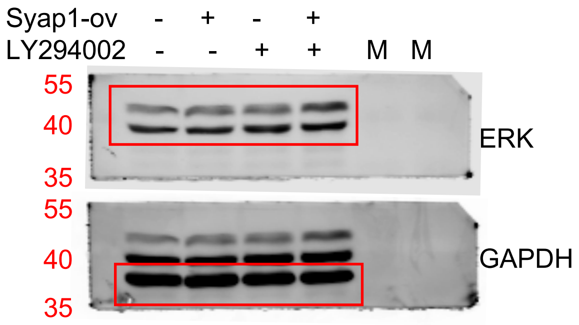

Syap1-ov - + - +
LY294002 - - + + M M
55
40
35
55
40
35
ERK
GAPDH

Fig S15A

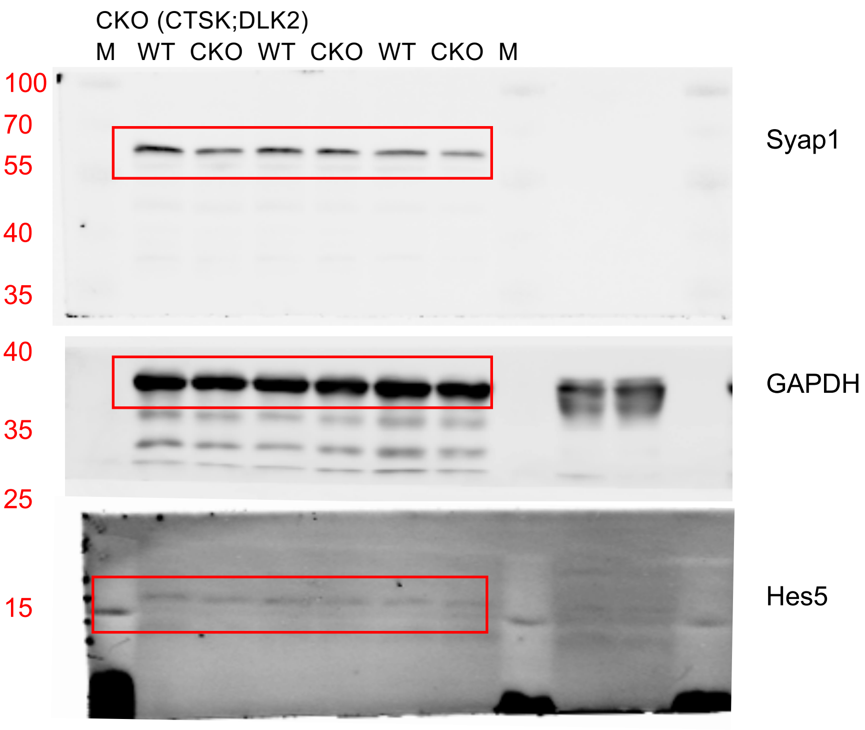
CKO (CTSK;DLK2)
M WT CKO WT CKO WT CKO M
100
70
55
40
35
Syap1
40
35
GAPDH
25
15
Hes5

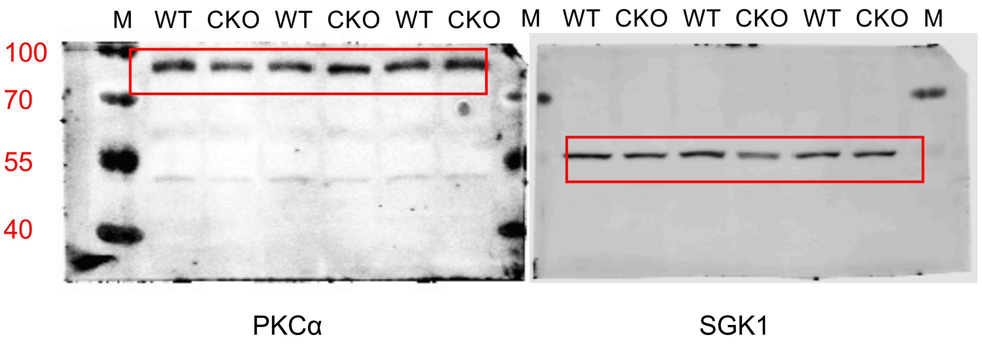
M WT CKO WT CKO WT CKO M WT CKO WT CKO WT CKO M
100
70
55
40
PKCα
SGK1

Fig S15A

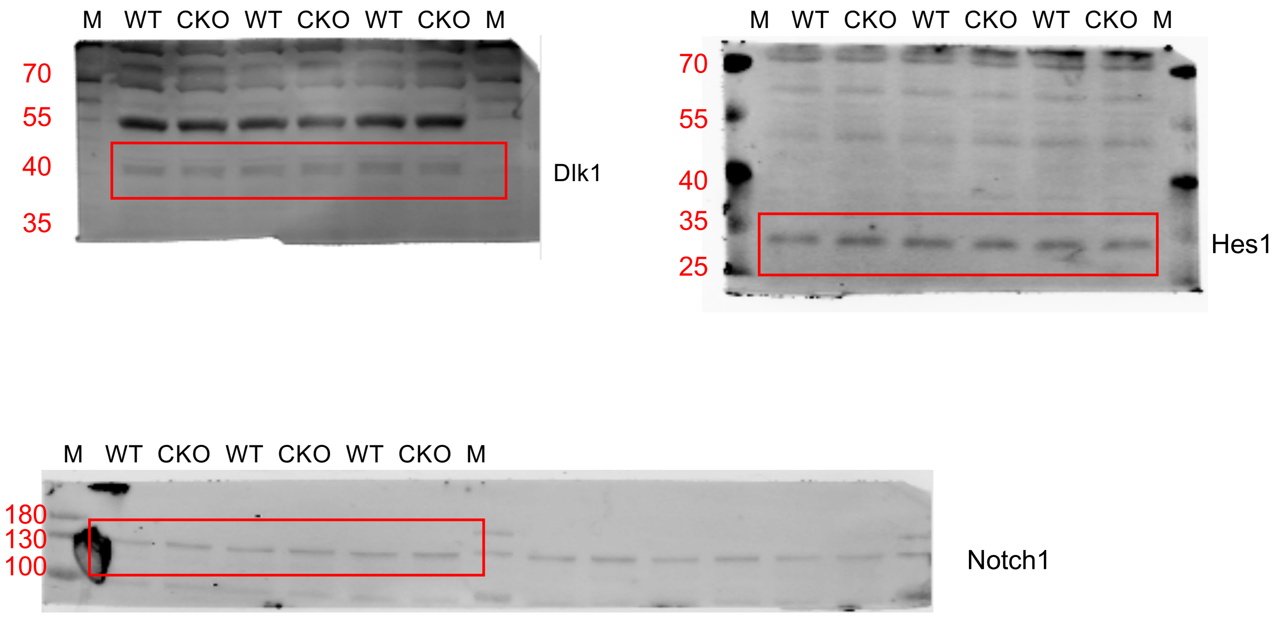
M WT CKO WT CKO WT CKO M
70
55
40
35
Dlk1
M WT CKO WT CKO WT CKO M
70
55
40
35
25
Hes1
M WT CKO WT CKO WT CKO M
180
130
100
Notch1

Fig S15B

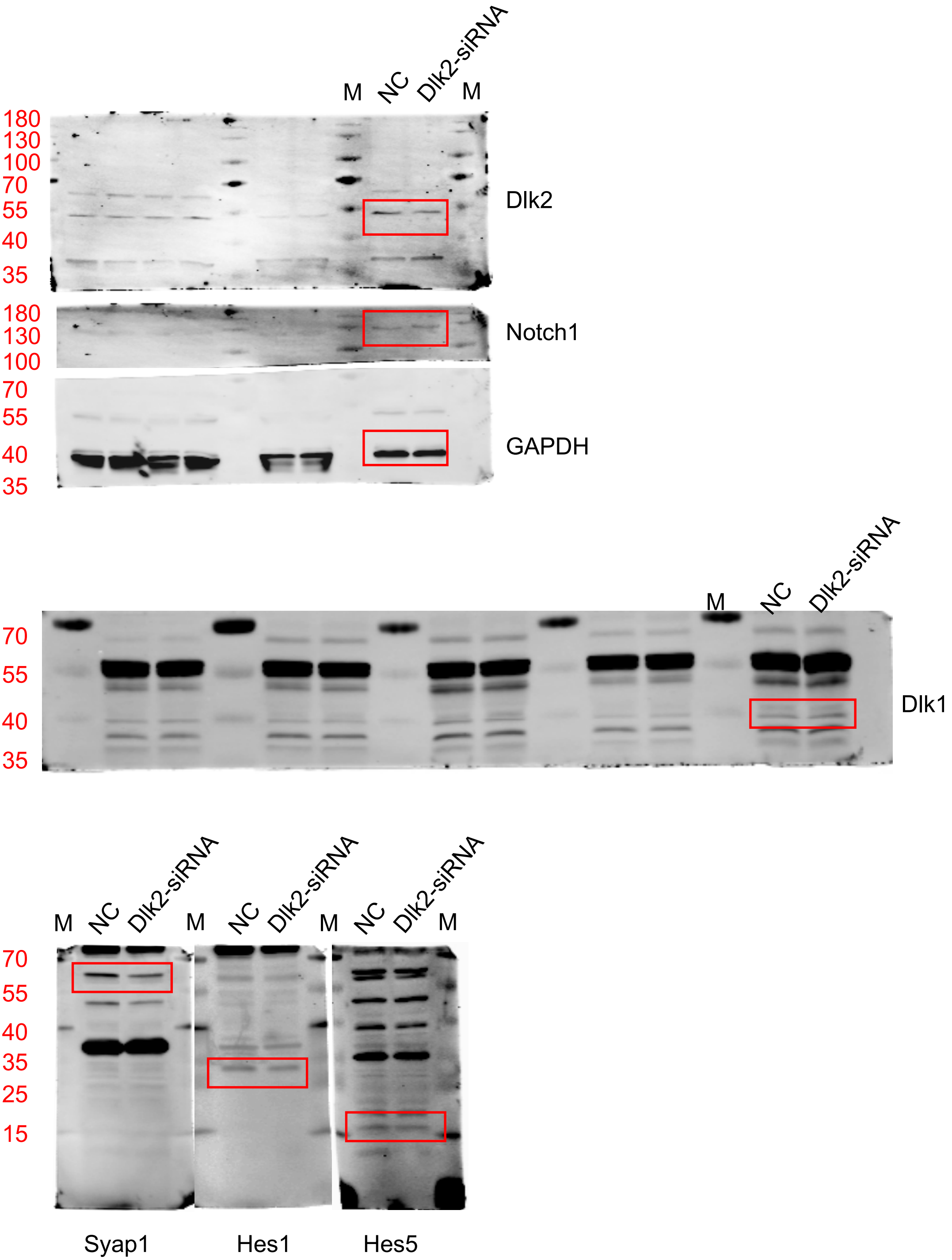
M
NC
Dlk2-siRNA
M
180
130
100
70
55
40
35
Dlk2
180
130
100
Notch1
70
55
40
35
GAPDH
M
NC
Dlk2-siRNA
70
55
40
35
Dlk1
M
NC
Dlk2-siRNA
M
NC
Dlk2-siRNA
M
NC
Dlk2-siRNA
M
70
55
40
35
25
15
Syap1
Hes1
Hes5

Fig S15C

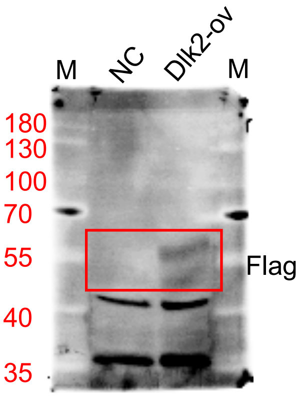
M
NC
Dlk2-ov
M
180
130
100
70
55
40
35
Flag

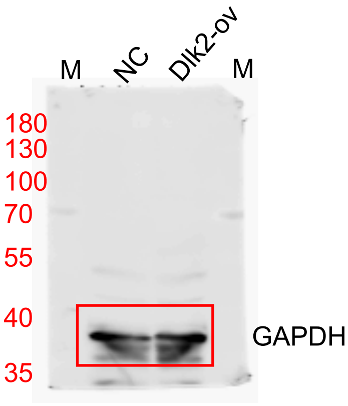
M
NC
Dlk2-ov
M
180
130
100
70
55
40
35
GAPDH

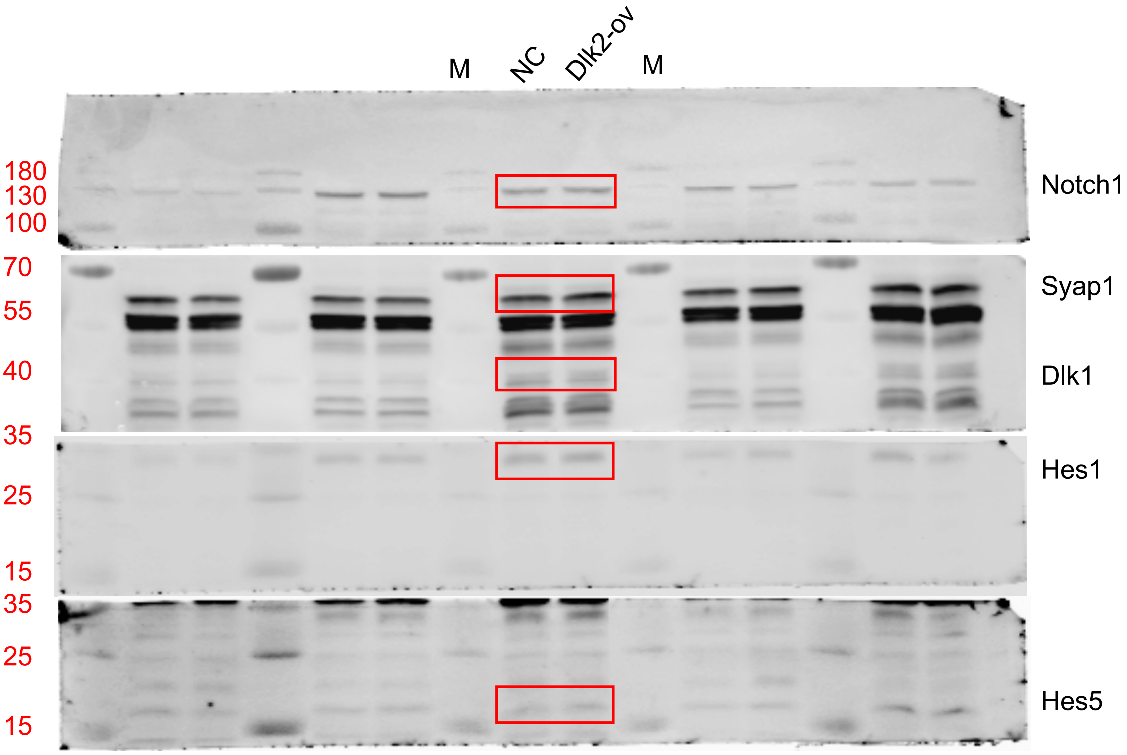
M
NC
Dlk2-ov
M
180
130
100
70
55
40
35
25
15
35
25
15
Notch1
Syap1
Dlk1
Hes1
Hes5
